# DCBLD2 Mediates Epithelial-Mesenchymal Transition-Induced Metastasis by Cisplatin in Lung Adenocarcinoma

**DOI:** 10.3390/cancers13061403

**Published:** 2021-03-19

**Authors:** Xiaosu Chen, Yajing Lv, Kejia Xu, Xiaoshuang Wang, Yujia Zhao, Jia Li, Xuan Qin, Yi Shi, Longlong Wang, Antao Chang, Chongbiao Huang, Rong Xiang

**Affiliations:** 1 The School of Medicine, Nankai University, Tianjin 300071, China; chenxs@mail.nankai.edu.cn (X.C.); 1120170508@mail.nankai.edu.cn (Y.L.); 2120191284@mail.nankai.edu.cn (K.X.); xswang@mail.nankai.edu.cn (X.W.); 1120170513@mail.nankai.edu.cn (Y.Z.); 1120170512@mail.nankai.edu.cn (J.L.); 1120170509@mail.nankai.edu.cn (X.Q.); yishi@nankai.edu.cn (Y.S.); wangl@nankai.edu.cn (L.W.); changantao@nankai.edu.cn (A.C.); 2 Department of Thoracic Cancer, Tianjin Medical University Cancer Institute and Hospital, National Clinical Research Center for Cancer, Key Laboratory of Cancer Prevention and Therapy, Tianjin 300060, China; 3 The International Collaborative Laboratory for Biological Medicine of the Ministry of Education, The School of Medicine, Nankai University, Tianjin 300071, China

**Keywords:** DCBLD2, cisplatin, epithelial-mesenchymal transition (EMT), metastasis, lung adenocarcinoma (LUAD)

## Abstract

**Simple Summary:**

Chemotherapeutic agents including cisplatin promote tumor metastasis while inhibiting tumor growth, which still represents a major obstacle for some patients in clinical practices. This study demonstrated that cisplatin induced epithelial-mesenchymal transition and tumor metastasis in lung adenocarcinoma. Further bioinformatic analysis showed that DCBLD2 may play a key role in metastasis after platinum chemotherapy. In terms of mechanism, DCBLD2 stabilized β-catenin through phosphorylation and inactivation of GSK3β, leading to the disintegration of the destruction complex of β-catenin. The accumulated β-catenin transported to the nucleus and promoted the expression of metastasis-related genes. In addition, cisplatin markedly enhanced DCBLD2 (Discoidin, CUB and LCCL domain containing 2) expression via ERK/AP-1 axis. Importantly, DCBLD2-specific siRNAs encapsulated by nanoparticles strikingly inhibited cisplatin-induced metastasis in tumor-bearing mice. Taken together, DCBLD2 mediates cisplatin-induced metastasis and DCBLD2 inhibition is a promising treatment option for preventing chemotherapy-induced metastasis.

**Abstract:**

Growing evidence suggests that cisplatin and other chemotherapeutic agents promote tumor metastasis while inhibiting tumor growth, which is a critical issue for certain patients in clinical practices. However, the role of chemotherapeutics in promoting tumor metastasis and the molecular mechanism involved are unclear. Here, we investigated the roles of cisplatin in promoting tumor metastasis in lung adenocarcinoma (LUAD). We demonstrated that cisplatin promoted epithelial-mesenchymal transition (EMT), cell motility, and metastasis in vitro and in vivo. The bioinformatic analysis and molecular biology approaches also indicated that DCBLD2 (Discoidin, CUB and LCCL domain containing 2) is a key gene that mediates cisplatin-induced metastasis. DCBLD2 stabilizes β-catenin by phosphorylating GSK3β and transporting accumulated β-catenin to the nucleus to promote the expression of EMT-related transcriptional factors (TFs), ultimately resulting in tumor metastasis. We also identified that cisplatin enhanced DCBLD2 expression by phosphorylating ERK and hence the AP-1-driven transcription of DCBLD2. Furthermore, DCBLD2-specific siRNAs encapsulated by nanocarriers prominently inhibit cisplatin-induced metastasis in vivo. Therefore, DCBLD2 plays a key role in cisplatin-induced metastasis in LUAD and is a potential target for preventing chemotherapy-induced metastasis in vivo.

## 1. Introduction

Lung cancer is the most frequently diagnosed cancer and the leading cause of cancer-related mortality worldwide [1,2]. Lung adenocarcinoma (LUAD) is the most diagnosed histological subtype, accounting for approximately 40% of all lung cancers and is currently on the rise [3]. Despite improvements in our understanding of the pathogenesis of LUAD and the development of treatments [4], tumor metastasis still represents a major obstacle for the effectiveness of conventional chemotherapies and is consequently one of the most serious threats to patient survival.

Platinum-based chemotherapy regimens comprising two or three agents are the standard first-line treatment for patients with LUAD; these regimens include the combination of cisplatin or carboplatin with pemetrexed or paclitaxel and with or without bevacizumab [5,6]. Cisplatin was the first platinum compound approved for cancer therapy worldwide in 1978 [7]. Although postoperative and preoperative neoadjuvant chemotherapeutics are effective in suppressing tumors and prolonging patient survival, a large number of preclinical and clinical reports indicate that chemotherapy probably increases the incidence of tumor metastasis [8,9]. Early studies showed that cyclophosphamide enhanced the formation of pulmonary metastatic nodules after intravenous tumor cell injection [10,11]. Other evidence indicated that short-term treatment of nonmetastatic breast carcinoma cells with chemotherapeutic agents, such as adriamycin and 5-fluoro-2′-deoxyuridine (FUdR), induced a metastatic phenotype in vitro and caused spontaneous lung metastasis within 10 weeks in vivo [12]. In clinical research, circulating tumor cell counts increased in blood samples of patients with breast cancer undergoing neoadjuvant chemotherapy and correlated with distant-metastasis-free survival [13,14]. These studies suggest that chemotherapeutic agents can either select more aggressive cells or enhance the metastatic potential of surviving cells. However, the molecular mechanism by which chemotherapeutics promote tumor metastasis is still unclear.

Discoidin, CUB and LCCL domain containing 2 (DCBLD2, also known as ESDN or CLCP1) was first identified from human coronary arterial cells by a signal sequence trap method and is mapped to human chromosome 3q12.1;3 [15]. DCBLD2, containing a CUB domain, an LCCL domain, and a coagulation factor V/VIII homology domain, is reported to localize on the plasma membrane and in the cytosol. DCBLD2 regulates proliferation, migration, and invasion in various tumors, including glioma, hypopharyngeal squamous cell carcinoma, and myxofibrosarcoma [16,17,18]. In glioblastoma and head and neck cancer, phosphorylated DCBLD2 recruits tumor necrosis factor receptor-associated protein 6 (TRAF6), which leads to the increased E3 ubiquitin ligase activity of TRAF6 and ubiquitin-mediated AKT activation, thereby enhancing EGFR-driven tumorigenesis [19]. In addition, DCBLD2 promotes VEGF-induced proliferation of endothelial cells and angiogenesis by preventing VEGFR-2 from forming complexes with the negative regulators protein tyrosine phosphatase PTP1B, TC-PTP, and VE-cadherin [20]. It was also reported that DCBLD2 was related to respiratory diseases in asthma patients, such as aspirin-exacerbated respiratory disease (AERD) and nasal polyps [21,22].

In this study, we focused on the cisplatin-induced metastasis in LUAD. The bioinformatic analysis indicated that after cisplatin treatment DCBLD2 expression was significantly higher in the LUAD patients diagnosed with distant metastasis than in those without metastasis. We further identified the crucial role and the mechanism of DCBLD2 in cisplatin-induced EMT and tumor metastasis.

## 2. Materials and Methods

### 2.1. Cell Lines and Clinical Samples

The human LUAD cell line A549 and cisplatin-resistant cell line A549/cis were purchased from the National Infrastructure of Cell Line Resources (Beijing, China). The human LUAD cell line Pc9 and cisplatin-resistant cell line Pc9/cis were obtained from the Type Culture Collection Committee of the Chinese Academy of Sciences (Shanghai, China). Cells were cultured in McCoy’s 5A or RPMI 1640 medium supplemented with 10% fetal bovine serum (FBS) and 1% penicillin-streptomycin at 37 °C in a humidified atmosphere containing 95% air and 5% CO_2_.

A total of 125 formalin-fixed and paraffin-embedded LUAD tissues and 17 adjacent nontumor lung tissues were collected from patients at Tianjin Medical University Cancer Institute and Hospital (China).

### 2.2. Bioinformatics Analyses

Data from The Cancer Genome Atlas (TCGA) comprising 515 patients with LUAD were downloaded from the TCGA website (http://cancergenome.nih.gov/, accessed on 2 December 2017). The normalized RNA expression levels of DCBLD2, TWIST1, SNAI1, SNAI2, and ZEB1; the clinicopathological parameters (including sex, age, pathologic type, and TNM stage.); chemotherapy response, and follow-up prognosis data were extracted and analyzed. In the setting of bioinformatics analysis and screening conditions, a *p* value less than 0.05 is set as statistically significant.

### 2.3. Plasmid Construction and Establishment of Stable Cell Lines 

The complete coding sequence of the human DCBLD2 gene (NM_080927.4) was cloned into pLV-EF1-MCS-IRES-Bsd vectors (Biosettia, San Diego, CA, USA). The lentiviruses were produced in 293T cells according to the manufacturer’s instructions. Then, the cells were infected with lentivirus for 24 h and cultured for 48 h, followed by selection using 2 μg/mL blasticidin. For the cell lines with stable knockdown, shRNA sequences were designed with BLOCK-iT™ RNAi Designer (https://rnaidesigner.thermofisher.com/rnaiexpress/, accessed on 27 March 2018). Five recommended sequences for DCBLD2 were synthesized and cloned into pLV-H1-EF1α-puro vectors (Biosettia, San Diego, CA, USA). The cells infected with RNAi lentiviruses were collected for western blot analysis and RT-PCR. Of the five stable cell lines created, two cell strains with the highest RNAi efficacy were used for subsequent assays. shDCBLD2-1#, 5′-GCATCAAATTTGGTGACTTTG-3′; and shDCBLD2-2#, 5′-GCAAGAGAACAGTTGGAAACC-3′.

### 2.4. RNA Isolation and RT-PCR

Total RNA from cells and tissues was isolated by TRIzol Reagent (Invitrogen, Waltham, MA, USA), and then 2 μg of RNA was reverse transcribed to cDNA with a reverse transcription PCR (RT-PCR) system (TaKaRa, San Jose, CA, USA). SYBR Green qRT-PCR was performed to analyze the cDNA levels. The relative expression levels of genes were calculated using the 2^−ΔΔCt^ method, and gene expression was normalized to that of β-actin (ACTB). The primers were as follows: DCBLD2 forward, 5′-CTCCTCGGAACAGCAATGACC-3′; DCBLD2 reverse, 5′-ATTCATTGCTACTGCGAGGTT-3′; TWIST1 forward, 5′-CCCACGCTGCCCTCGGACA-3′; TWIST1 reverse, 5′-CCATCCTCCAGACCGAGAAGGCGTA-3′; SNAI1 forward, 5′-CCTTCGCTGACCGCTCCAACCTG-3′; SNAI1 reverse, 5′-ACATCCTGAGCAGCCGGACT-3′; SNAI2 forward, 5′-CCCCTCCTCCATCTGACACC-3′; SNAI2 reverse, 5′-AAAGATTTTCTAGACTGGGCATCG-3′; ZEB1 forward, 5′-TCTGATTCTACACCGCCCAA-3′; ZEB1 reverse, 5′-CCATCCTCCAGACCGAGAAGGCGTA-3′; ACTB forward, 5′-CTACCTTCAACTCCATCATGAAGTG-3′; and ACTB reverse, 5′-CATTTGTCACATTGATAGGGCTT-3′. 

### 2.5. Western Blot

Whole cell extracts were prepared by lysing cells with radioimmunoprecipitation assay (RIPA) lysis buffer supplemented with a proteinase inhibitor cocktail (Sigma-Aldrich, St Louis, MO, USA). Protein concentrations were quantified by a BCA protein assay kit (Thermo Fisher Scientific, Waltham, MA, USA). A total of 20 μg of protein lysate was separated by SDS–PAGE and transferred onto polyvinylidene fluoride (PVDF) membranes, which were submerged in 5% skim milk in TBST for 1 h at room temperature to block nonspecific interactions before they were incubated with primary antibodies against DCBLD2 (PA5-28547, Thermo Fisher Scientific, Waltham, MA, USA), E-cadherin (610182, BD Biosciences, San Diego, CA, USA), ZO-1 (#5406, Cell Signaling Technology, Danvers, MA, USA), Vimentin (#5741, Cell Signaling Technology, Danvers, MA, USA), N-cadherin (22018-1-AP, Proteintech, Wuhan, China), Zeb1 (ab203829, Abcam), Snail (#3879, Cell Signaling Technology, Danvers, MA, USA), β-catenin (#8480, Cell Signaling Technology, Danvers, MA, USA), GSK3β (#9832, Cell Signaling Technology, Danvers, MA, USA), p-GSK3β (Ser9) (#9336, Cell Signaling Technology, Danvers, MA, USA), ERK (sc-514302, Santa Cruz Biotechnology, Dallas, TX, USA), p-ERK (Thr202/Tyr204) (bs-3016R, Bioss, Edinburgh, UK), c-Fos (ab222699, Abcam, Cambridge, UK), c-Jun (ab31419, Abcam, Cambridge, UK), α-Tubulin (#2144, Cell Signaling Technology, Danvers, MA, USA), Lamin A/C (#4777, Cell Signaling Technology, Danvers, MA, USA) and β-actin (SC-47778, Santa Cruz Biotechnology, Dallas, TX, USA) overnight at 4 °C. Membranes were further incubated with corresponding horseradish peroxidase (HRP)-conjugated secondary antibodies for 1 h at room temperature, and protein bands were visualized using ECL (Millipore, Burlington, MA, USA). The uncropped western blot images are shown in Figure S7.

### 2.6. Immunofluorescence (IF) Staining and Microscopy

IF staining was performed on the LUAD cell lines. After fixed with 4% fresh paraformaldehyde and permeabilized with 0.2% Triton X-100, cells were incubated with 5% goat serum for 1 h to block nonspecific interactions. Then the cells were incubated with primary antibodies against E-cadherin, Vimentin, or β-catenin (1:200) at 4 °C overnight and followed by fluorescent-dye-labeled secondary antibodies (1:200) (Thermo Fisher Scientific, Waltham, MA, USA) for 1 h and again incubated with DAPI (1:1000) (Sigma) for 3 min. Extensive washes with PBS were performed between each step. Finally, the coverslips were mounted with Fluoromount media, and the images were captured with a confocal fluorescence microscope (Olympus, Tokyo, Japan).

### 2.7. Immunohistochemistry (IHC) Staining

After deparaffinization and antigen retrieval, the serial tissue sections were incubated with primary antibodies against DCBLD2, E-cadherin, and Vimentin (1:200) overnight at 4 °C. Then, the cells were treated with a peroxidase-conjugated secondary antibody at 37 °C for 1 h before the DAB Substrate Kit was used to reveal bound secondary antibody. The semiquantitative H score was used to estimate the immunoreactivity according to the following criteria: staining intensity was scored as 0 (negative), 1 (low), 2 (medium), or 3 (high); and staining extent was scored as 1 (0–15% stained), 2 (16–50% stained), 3 (51–80% stained), or 4 (81–100% stained). The H score was calculated by multiplying the intensity and extent scores and ranged from 0 to 12; the scores were assigned the following categories 0, negative (–); 1–2, low staining (+); 3–6, medium staining (++); and >6, high staining (+++).

### 2.8. Chromatin Immunoprecipitation Assay (ChIP)

Chromatin immunoprecipitation (ChIP) assays were performed using a commercial kit (Millipore, Burlington, MA, USA) according to the manufacturer’s instructions. The DCBLD2 primers were forward, 5′-GAGTTGAAACATACGACCTCC-3′, and reverse, 5′-TAACAGGCAGATGGATATTTTAGTCGAA-3′. In brief, A549/cis cells were cultured in the presence or absence of 2 μg/mL cisplatin for 24 h before they were immunoprecipitated with anti-c-Fos and anti-c-Jun antibodies. The immunoprecipitated products were detected by PCR and RT-PCR analysis.

### 2.9. Cisplatin Resistance Assays

The cells’ sensitivity to cisplatin was assessed by a CCK-8 kit (Dojindo Molecular Technologies, Rockville, MD, USA) according to the manufacturer’s manual. Briefly, 5000 cells in 100 μL were seeded per well of a 96-well plate and cultured with various concentrations of cisplatin (CAS No. 15663-27-1) for 24 h. Then, 10 μL of CCK-8 solution was added to each well, and the plate incubated for 1 h at 37 °C. The absorbance at 450 nm was detected by a microplate reader (Promega, Madison, WI, USA).

### 2.10. Cell Migration Assays

A high-content cell imaging and analysis system (HCS) is a quantitative analysis technology based on live-cell imaging that can automatically obtain cell images and analyze the moved distance, speed, and unidirectional displacement of single cells without a fluorescent label. A total of 4000 cells were seeded in a 24-well plate and pretreated with 2 μg/mL cisplatin or vehicle control for 24 h. Then, cell migration was automatically tracked every 30 min for a total of 16 h by high-content screening. The images and movies of single-cell motion tracks were generated by the RMS cell tracking migration analysis module in Harmony software. The migration was represented by a displacement dot plot and expressed as the average speed (nm/s) per track.

Transwell assays were performed with an 8-μm pore Transwell chamber (Millipore, Burlington, MA, USA) in 24-well plates. In all, 1 × 10^5^ cells resuspended in 200 μL of McCoy’s 5A or RPMI 1640 medium supplemented with 1% FBS were seeded in the upper chamber of the Transwell unit. The lower chamber contained 10% FBS as a chemoattractant. After the cells incubated for 8 h, nonmigrating cells on the polycarbonate membrane surface in the upper chamber were removed while cells that migrating to the membrane surface adjacent to the lower chamber were stained with 0.1% crystal violet staining solution and counted. Four random fields were analyzed per chamber. All experiments were repeated independently at least three times. 

A wound healing assay was performed to detect cell migration in vitro. Cells were grown to 100% confluency in a 6-well plate before a wound was made by scraping a 200-μL pipette tip across the monolayer. Then, the cells were incubated with McCoy’s 5A or RPMI-1640 medium supplemented with 1% FBS until the wound closed. Images were recorded at 0 h, 12 h, 24 h, and 48 h after scratching or until the wound closed to depict cell motility. All experiments were repeated independently at least three times. Migration was expressed as a percentage of the wound area covered by migratory cells.

### 2.11. Animal Studies and In Vivo Assessment of Metastasis in an Orthotopic Lung Adenocarcinoma Mouse Model

Six-week-old male BALB/c nude mice were maintained in a specific pathogen-free facility. Animal studies were conducted in accordance with the Animal Welfare Guidelines of Nankai University. Cisplatin for injection was purchased from Hansoh Pharma (Jiangsu, China). After harvested by trypsinization and washed in PBS for 3 times, cells were resuspended at a density of 10^7^ cells per mL in a 1:1 solution of serum-free RPMI 1640 medium and Matrigel (Corning, New York, NY, USA), and injected into the middle lobe of the right lungs of BALB/c nude mice.

To confirm whether cisplatin induces metastasis, 1 × 10^6^ luciferase-labeled Pc9/cis cells were injected into the right lungs of 16 BALB/c nude mice. Seven days after injection, the mice were divided into two groups (PBS and cisplatin, 8 mice each) and intraperitoneally injected every 7 days with 4.0 mg/kg cisplatin or an equivalent volume of PBS. Thirty-five days after tumor cell inoculation, the mice underwent bioluminescence imaging and were sacrificed. Primary tumors were harvested from the right lung of the mice, while metastatic foci were harvested from the left lung. The lung tissues were fixed with formalin, embedded in paraffin, and subjected to HE staining or IHC staining.

To confirm whether DCBLD2 mediates cisplatin-induced metastasis, 21 BALB/c nude mice were divided into three groups (sc + PBS, sc + cisplatin and shDCBLD2 +cisplatin; 7 mice each), and 1 × 10^6^ luciferase-labeled Pc9/cis cells were injected into the right lung of the mice. Seven days after inoculation, the mice were intraperitoneally injected with 4.0 mg/kg cisplatin or an equivalent volume of PBS every 7 days. Thirty-five days after inoculation, bioluminescence imaging was performed as indicated to monitor metastasis in the left lung. The primary tumors in the right lung and the metastatic foci in the left lung were subjected to HE staining or IHC staining.

To confirm whether DCBLD2 promotes metastasis, 16 BALB/c nude mice were divided into two groups (vector and DCBLD2, 8 mice each), and 1 × 10^6^ A549 cells were injected into the right lung of the mice. Four weeks later, the lungs were removed, fixed with formalin and embedded in paraffin for HE staining or IHC staining, and the number of metastatic foci in the left lung was determined. Similarly, 12 BALB/c nude mice were divided into two groups (sc and shDCBLD2, 6 mice each), and 1 × 10^6^ A549 cells were injected into the right lung of BALB/c nude mice. Six weeks later, the lungs were removed, fixed with formalin and embedded in paraffin for HE staining or IHC staining.

For translational experiments, in vivo-jetPEI nanocarriers was used to provide safe and efficient delivery of siRNAs targeting DCBLD2. Tumor-bearing mice were intravenously injected with jetPEI-si scramble (40 mg), jetPEI-si DCBLD2 (40 mg), or 5% glucose control (200 μL) (6 mice per group) every week for 4 weeks starting on day 14 after tumor inoculation.

### 2.12. Statistical Analysis

The data are presented as the mean ± standard deviation. Shapiro–Wilk test (*p* > 0.05) was used to confirm the quantitative data conform to the normal distribution. A two-tailed Student’s *t*-test was performed to compare the differences between the two groups. A paired Student’s *t*-test was used to compare DCBLD2 expression between LUAD tissues and adjacent normal tissues. Wilcoxon’s rank test was performed to analyze IHC staining intensities of DCBLD2 in paired tumor and nontumor tissues. Spearman’s r test was used to determine the correlation between DCBLD2 and EMT-related markers or TFs. A two-way ANOVA test was used to analyze the continuous variables of the proliferation curve. Categorical data were analyzed by the Chi-square test was used to analyze the difference in clinicopathological categorical data. Kaplan–Meier survival curves and Log-rank test presented the difference in overall survival (OS) and relapse-free survival (RFS) based on different DCBLD2 expression. GraphPad Prism 8 or IBM SPSS 22.0 software was used to perform the statistical analyses.

## 3. Results

### 3.1. Cisplatin Notably Facilitates EMT, Cell Migration, and Metastasis in LUAD

Solid tumors are highly heterogeneous with cell subpopulations exhibiting differential cell viability, migration and invasion abilities, and sensitivity to chemotherapy. Chemotherapy objectively screens resistant cancer cells, leading to the promotion of cell migration and metastasis. More LUAD patients who received chemotherapy were diagnosed with distant metastasis, locoregional recurrence, or new primary tumor than those who did not receive chemotherapy (34.08% and 22.45%, respectively; *p* < 0.01) (Figure S1a, left panel). In particular, more patients were diagnosed with distant metastases after chemotherapy than those who did not receive chemotherapy (9.62% and 18.44%, respectively; *p* < 0.01) (Figure S1a, right panel). Therefore, we focused on a subset of cells that are not sensitive to chemotherapy and selected the cisplatin-resistant human LUAD cell lines A549/cis and Pc9/cis. A significant increase in the IC_50_ concentrations was observed in both cell lines represented by 6.88-fold and 5.69-fold increases in A549/cis and Pc9/cis cells, respectively, compared to their parental cell lines (Figure S1b).

**Figure 1:**
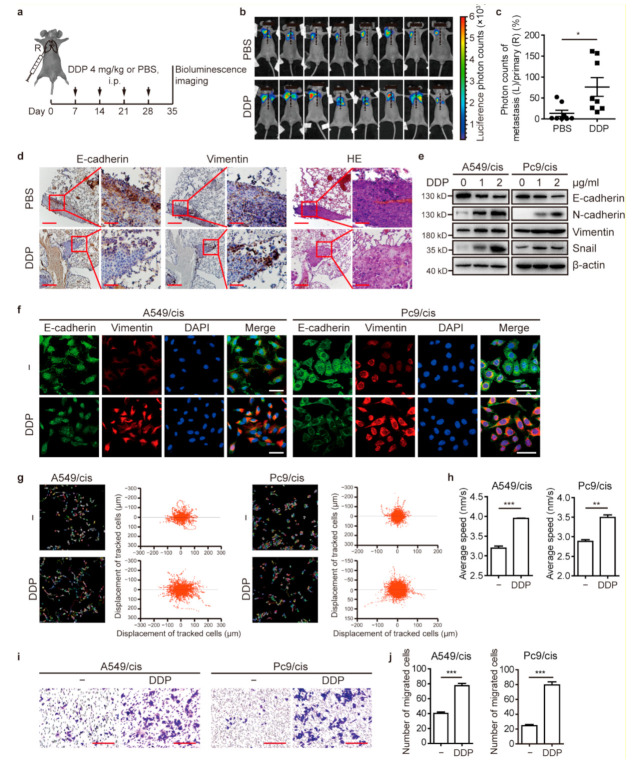
Cisplatin promotes EMT and tumor metastasis in chemotherapy-resistant LUAD. (**a**) Experimental design and chemotherapy administration. Pc9/cis-luciferase cells were inoculated into the right lung of BALB/c nude mice (*n* = 8 per group) to establish an orthotopic tumor xenograft model. On day 7, mice were intraperitoneally injected with 4.0 mg/kg cisplatin or an equivalent volume of PBS every 7 days. Thirty-five days after tumor cell inoculation, mice underwent a bioluminescence test and were sacrificed. The primary tumors in the right lung and metastatic foci in the left lung were subjected to IHC staining. (**b**,**c**) Bioluminescence imaging (**b**) and quantitation (**c**) from mice with orthotopic tumor xenografts. (**d**) Representative IHC staining images of E-cadherin and Vimentin expression in paraffin-embedded serial sections of the primary lesion (right lung) from orthotopically implanted mice from Figure 1a. Scale bar: 200 μm (left) and 50 μm (right). (**e**) Western blot analysis of EMT proteins. Cells were cultured with or without cisplatin for 24 h. (**f**) Representative IF images of EMT markers and DAPI in cells treated with 2 μg/mL cisplatin or vehicle control for 24 h. Scale bar: 50 μm. (**g**,**h**) Dynamic imaging analyses of cell migration. Cells were pretreated with 2 μg/mL cisplatin or vehicle control for 24 h. Cell migration was tracked over 16 h by HCS. Representative images of the single-cell motion track (left panel) and single-cell ultimate displacement (right panel) are shown in (**g**). The average speed was measured in (**h**). (**i**,**j**) Transwell assay confirming the effect of cisplatin on cell migration. Images of the cells on the underside of the membrane are shown in (**i**) after culture for 6 h in the presence of a chemoattractant. Scale bar: 200 μm. Quantification of migrated cells is depicted in (**j**). * *p* < 0.05, ** *p* < 0.01, *** *p* < 0.001, *t*-test. DDP, refers to cis-diamminedichloridoplatinum (II), also known as cisplatin or cisplatinum.

To investigate whether cisplatin promotes EMT, cell migration, and metastasis, we first evaluated the effect of cisplatin on tumor metastasis. Luciferase-transfected Pc9/cis cells were orthotopically injected into the right lungs of nude mice as previously described [23]. After tumor formation, mice were treated with cisplatin or an equivalent volume of vehicle regularly to investigate the effect of cisplatin on metastasis. At 35 days after cell implantation, the primary tumor (right lung) and metastatic foci (left lung) were monitored by in vivo bioluminescence imaging (Figure 1a). The results demonstrated that cisplatin treatment enhanced metastasis to the left lung in the orthotopic xenograft model (Figure 1b,c).

The expression of the epithelial marker E-cadherin and the mesenchymal marker Vimentin was detected in tumor tissues. IHC staining confirmed lower expression of E-cadherin and higher expression of Vimentin in tumor tissues of mice treated with cisplatin compared to those of vehicle-treated mice (Figure 1d). Furthermore, western blot analysis indicated that the expression of epithelial markers E-cadherin significantly decreased, while mesenchymal markers (Vimentin and N-cadherin) and the EMT transcriptional factor Snail increased after cisplatin treatment (Figure 1e). These changes in E-cadherin and Vimentin expression after treatment with cisplatin were confirmed by IF assays (Figure 1f).

Next, we investigated the effect of cisplatin on cell migration by a high-content cell imaging and analysis system (HCS). Compared to control cells, cells treated with cisplatin had more chaotic movement tracks, more dispersive overall displacement of single cells, and a faster average speed (Figure 1g,h). In the Transwell assays, the migration of A549/cis and Pc9/cis cells treated with cisplatin was significantly increased compared with that of untreated cells (Figure 1i,j). Similarly, the results of the wound healing assays showed that cisplatin enhanced migratory ability of the cells (Figure S1c,d). Taken together, our data indicated that cisplatin induced EMT, migration, and metastasis in LUAD.

### 3.2. DCBLD2 Is Essential for Cisplatin-Induced EMT and Metastasis

**Figure 2:**
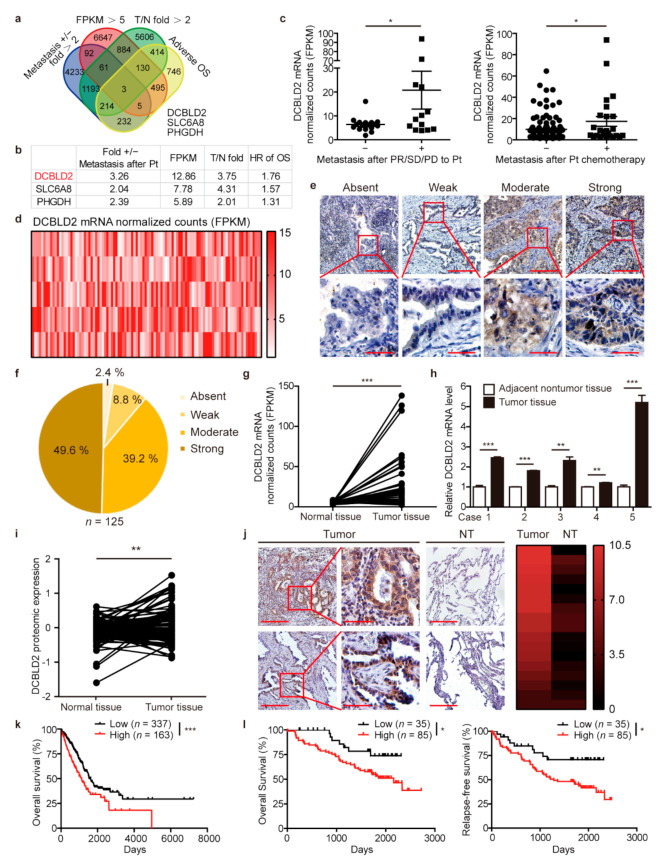
DCBLD2 is a candidate gene involved in cisplatin-induced metastasis. (**a**) Screening strategy for key genes related to distant metastasis after platinum-based chemotherapy. (**b**) Ranking of the 3 identified candidate genes according to the 4 criteria. (**c**) Differential expression of DCBLD2 at the transcriptional level in LUAD patients from the TCGA database with or without distant metastasis when responding to platinum agents with a partial response, stable disease, or progressive disease (left). DCBLD2 expression in LUAD patients with distant metastasis who received platinum agents (right). * *p* < 0.05, *t*-test. (**d**) The heat map of DCBLD2 expression in mRNA normalized count (log 2) in LUAD tissues from the TCGA database (*n* = 515). (**e**,**f**) Representative images (**e**) and distribution (**f**) of DCBLD2 expression in LUAD tissues by IHC assay (*n* = 125). Scale bar: 200 μm (top), 50 μm (bottom). (**g**) Transcriptional analysis of the differential expression of DCBLD2 in paired LUAD tissues and normal tissues from the TCGA database (*n* = 57), *p* = 0.0003, paired *t*-test. (**h**) DCBLD2 expression at the mRNA level in LUAD tissues and adjacent nontumor tissues by RT-PCR (*n* = 5). ** *p* < 0.01, *** *p* < 0.001, *t*-test. (**i**) Differential expression of DCBLD2 at the protein level in LUAD tissues and paired normal tissues from the CPTAC database (*n* = 102), ** *p* < 0.01, paired *t*-test. (**j**) Representative images of DCBLD2 expression in LUAD tissues and paired nontumor tissues (*n* = 17) by IHC assay. Scale bar: 200 μm (left), 50 μm (middle), 200 μm (right). Wilcoxon’s rank tests, Z = −3.524, *p* < 0.001. (**k**) Kaplan-Meier analysis of OS of 500 LUAD patients from the TCGA database stratified by DCBLD2 levels. The cutoff for DCBLD2 expression was 9.5 FPKM. Log-rank tests, *p* = 0.0002. (**l**) Kaplan-Meier analysis of OS and RFS of 120 LUAD patients according to different DCBLD2 levels. The cutoff line for H score was 5. Log-rank tests, *p* = 0.0418 and *p* = 0.0140, respectively.

To identify the genes that mediate cisplatin-induced metastasis, the following four conditions were set to screen for potential candidates: (1) The expression of the gene was more than two times higher in patients diagnosed with distant metastasis after achieving a partial response (PR), presenting stable disease (SD) or presenting clinical progressive disease (PD) upon platinum-based chemotherapy treatment than in those without metastasis. (2) The average mRNA expression was more than five FPKM among LUAD patients in the TCGA database. (3) The expression of the gene was more than two times higher in LUAD tissues than in normal tissues. (4) The gene was associated with poor prognosis. For conditions (1), (3), and (4), *P* value less than 0.05 is considered statistically significant. Among 60483 genes, there were 6033 genes notably upregulated in patients diagnosed with distant metastasis after platinum chemotherapy, 8317 genes whose expression was more than five FPKM, 8505 genes significantly upregulated in LUAD tissues compared to normal tissues, and 2239 genes negatively correlated with prognosis. Three genes met all four conditions (Figure 2a). Since DCBLD2 ranked at the top of the 3 identified genes, we chose DCBLD2 for further investigation (Figure 2b).

DCBLD2 mRNA level in patients diagnosed with metastasis after PR, SD or PD to platinum chemotherapy was 3.26 times higher than that of nonmetastatic patients (Figure 2c, left panel). Upon expanding the clinical samples to patients who received platinum chemotherapy, we found that DCBLD2 expression in metastatic patients after receiving platinum chemotherapy was 1.76 times more than that in nonmetastatic patients (Figure 2c, right panel).

### 3.3. DCBLD2 Is Characterized as an Oncogene in Human LUAD

To further characterize DCBLD2 expression in LUAD, we analyzed the mRNA expression of DCBLD2 in 515 LUAD tissues from the TCGA database. The heat map revealed that DCBLD2 was widely upregulated in LUAD tissues (Figure 2d). Similarly, we performed IHC staining in a tissue microarray (TMA) containing 125 clinical samples of LUAD obtained from Tianjin Medical University Cancer Institute and Hospital. We found that 49.60% of the samples showed strong positive (+++) staining, 39.20% showed moderately positive (++) staining, 8.80% showed weakly positive (+) staining, and 2.40% showed no (-) staining. These data indicate that DCBLD2 is widely and notably overexpressed in LUAD at the protein level (Figure 2e,f).

To investigate the differential expression of DCBLD2 mRNA, we compared DCBLD2 mRNA expression in 57 LUAD specimens, including tumor tissues and paired normal tissues, from the TCGA database. The results showed that the mRNA expression of DCBLD2 in LUAD tumor tissues was higher than that in normal tissues (*p* = 0.0003) (Figure 2g). Next, we examined DCBLD2 mRNA expression in five pairs of LUAD and adjacent nontumor tissues from patients in Tianjin Medical University Cancer Institute and Hospital by RT-PCR. Our data revealed that DCBLD2 mRNA expression was remarkably increased in tumor tissues compared with normal lung tissues (Figure 2h).

To detect DCBLD2 protein expression in LUAD, we evaluated 102 paired LUAD tissues and normal tissues from the CPTAC database and found that DCBLD2 expression was higher in tumor tissues than in normal tissues (*p* = 0.0049) (Figure 2i). In addition, we examined DCBLD2 protein level by IHC staining in 17 pairs of LUAD tissues and adjacent nontumor tissues obtained from Tianjin Medical University Cancer Institute and Hospital. The results showed that DCBLD2 expression was significantly higher in tumor tissues than that in adjacent tissues (Z = −3.524, *p* < 0.001) (Figure 2j).

Next, we analyzed the correlation between DCBLD2 mRNA expression and clinicopathological features in LUAD patients from the TCGA database (*n* = 513, excluding 2 patients without clinical data). There was no correlation between DCBLD2 expression and either sex or age among LUAD patients. However, higher DCBLD2 expression was associated with lymph node metastasis (χ^2^ = 7.360, *p* < 0.01) and advanced TNM staging (χ^2^ = 6.063, *p* < 0.05) (Table 1). Similarly, we found the same conclusions in the patient cohort of Tianjin Medical University Cancer Institute and Hospital. The expression of DCBLD2 in protein level is positively correlated with lymph node metastasis (χ^2^ =7.119, *p* < 0.01) and TNM staging (χ^2^ = 6.406, *p* < 0.05) (Table 2). Importantly, Kaplan–Meier analysis of 500 LUAD patients from the TCGA database (Excluding 15 patients with missing prognostic information) indicated that higher DCBLD2 mRNA expression was associated with adverse overall survival (OS) (cut off = 9.5 FPKM, *p* = 0.0002, 1135 and 1622 days, respectively) (Figure 2k). We then performed a Kaplan-Meier analysis of the patients from Tianjin Medical University Cancer Institute and Hospital (*n* = 120, excluding five patients who lost follow-up) to verify the correlation between DCBLD2 protein expression and clinical prognosis. LUAD patients with strong (+++) or moderate (++) DCBLD2 protein expression had significantly worse OS and relapse-free survival (RFS) than did patients with weak (+) or absent (−) DCBLD2 protein expression (*p* = 0.0418 and *p* = 0.0140, respectively) (Figure 2l).

These findings support that DCBLD2 is a potential mediator in cisplatin-induced metastasis.

### 3.4. DCBLD2 Markedly Promotes Migration and Metastasis

**Figure 3:**
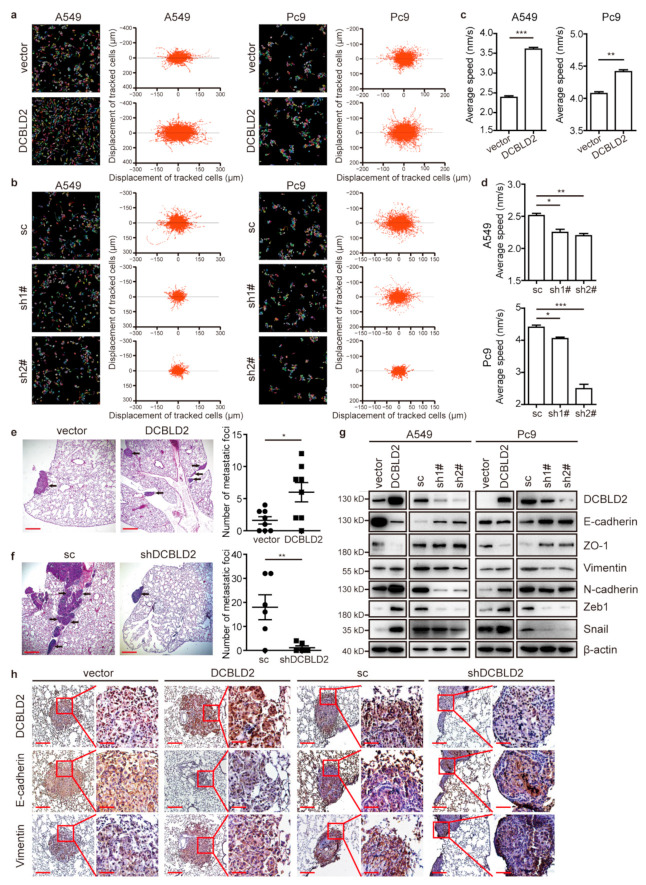
DCBLD2 induces migration and metastasis in LUAD. (**a**–**d**) Dynamic imaging analyses of cell migration in A549 and Pc9 cells. Cell migration was measured over 16 h by HCS. Representative images (left panel) and single-cell ultimate displacement (right panel) of A549 and Pc9 cells are shown in (**a**,**b**). The average speed was measured by Harmony software in (**c**,**d**). (**e**) A total of 1 × 10^6^ empty vector or DCBLD2-overexpressing cells were inoculated into the right lungs of BALB/c nude mice (*n* = 8 per group) to establish an orthotopic tumor xenograft model. Six weeks after inoculation, mice were sacrificed. The metastatic foci in the left lung were subjected to HE staining (left panel), and statistical analysis of the number of metastatic foci in the left lung is shown in the right panel. Scale bar: 400 μm. (**f**) A total of 1 × 10^6^ scramble control or shDCBLD2 A549 cells were inoculated in the right lung of BALB/c nude mice (*n* = 6 per group) to establish an orthotopic tumor xenograft model. Eight weeks after inoculation, mice were sacrificed. The metastatic foci in the left lung were subjected to HE staining (left panel), and statistical analysis of the number of metastatic foci in the left lung is shown in the right panel. Scale bar: 400 μm. (**g**) Western blot analysis of EMT markers in A549 and Pc9 cells. DCBLD2 was overexpressed or knocked down in cells, and western blot analysis of the expression of EMT-related proteins was performed. (**h**) Representative IHC images of the expression of E-cadherin and Vimentin in the primary lesion (right lung) of mice with orthotopic tumors derived from empty vector and DCBLD2-transfected cells or scramble control and shDCBLD2 cells. Scale bar: 200 μm (left), 50 μm (right). * *p* < 0.05, ** *p* < 0.01, *** *p* < 0.001, *t*-test.

To determine the role of DCBLD2 in LUAD, we established cell lines in which DCBLD2 was stably overexpressed or knocked down. Cell migration analysis by HCS showed that the migration tracks of A549 and Pc9 cells stably overexpressing DCBLD2 were more disorganized and that the distribution of dots was more dispersive, indicating that DCBLD2 significantly promoted cell migration (Figure 3a,c). By contrast, knocking down DCBLD2 in A549 and Pc9 cells evidently reduced cell migration (Figure 3b,d). In the Transwell assays, the migration of A549 and Pc9 cells was also enhanced by DCBLD2 overexpression (Figure S2a–d), and the wound healing assays indicated the same results (Figure S2e–h). To exclude the influence of cell proliferation and apoptosis, we showed that whenever DCBLD2 was overexpressed or silenced, the cell viability and apoptosis were not affected, as shown by CCK-8 and FACS assays (Figure S2i–k). Thus, we confirmed that DCBLD2 was able to promote cell migration in vitro.

The in vivo efficacy of DCBLD2 was evaluated in orthotopic LUAD xenograft models established using A549 cell lines. Overexpression of DCBLD2 significantly increased the number of metastatic foci in the left lung (Figure 3e). By contrast, DCBLD2 knockdown markedly reduced tumor metastasis (Figure 3f). Taken together, these data demonstrate that DCBLD2 prominently facilitates metastasis in vivo.

### 3.5. DCBLD2 Dramatically Enhances EMT in LUAD

Next, we explored the mechanism by which DCBLD2 promotes migration and metastasis in LUAD. RNA sequencing was performed in A549 cells transfected with DCBLD2 overexpression vector and empty vector. We assessed the RNA-seq findings using Hallmark analysis and found positive enrichment of EMT signatures (Figure S3a,b). Moreover, gene set enrichment analysis (GSEA) showed that genes associated with EMT, which is closely related to tumor metastasis, were significantly enriched in the DCBLD2-high samples among the LUAD specimens from the TCGA database (Figure S3c).

Therefore, we postulated that DCBLD2 might regulate EMT. A549 and Pc9 cells transfected with the DCBLD2 overexpression vector lost the expression of the epithelial markers E-cadherin and ZO-1 and acquired expression of the mesenchymal markers N-cadherin and Vimentin, with enhanced expression of the core EMT TFs Zeb1 and Snail. However, after transfection with shDCBLD2, the transition from epithelial to mesenchymal markers was attenuated significantly (Figure 3g). Similarly, IF analysis confirmed the regulation of E-cadherin and Vimentin expression by DCBLD2 (Figure S3d).

We further examined E-cadherin and Vimentin expression in an orthotopic xenograft model. DCBLD2 significantly reduced E-cadherin expression and increased Vimentin expression in tumor tissues in situ, as shown by IHC staining (Figure 3h). We also mixed DCBLD2-overexpessing A549 cells and empty vector-transfected cells at a ratio of 1:1 before injecting them subcutaneously into mice to generate the tumor xenografts composed of cells with heterogeneous expression of DCBLD2. IHC analysis of the relationship between DCBLD2 and EMT markers showed that DCBLD2 expression was negatively correlated with that of E-cadherin but positively correlated with that of Vimentin, suggesting that DCBLD2 potentiates EMT at both the cellular level and in mice models (Figure S3e).

Next, we verified the relationship between the expression of DCBLD2 and that of the epithelial marker E-cadherin and the mesenchymal marker Vimentin in 122 LUAD tissues (Excluding three incomplete specimens). DCBLD2 expression was negatively correlated with E-cadherin expression (Spearman’s r = −0.536, *p* < 0.0001) and positively correlated with Vimentin expression (Spearman’s r = 0.674, *p* < 0.0001) (Figure S3f,g). Consistently, correlation analyses of the mRNA expression of TWIST1, SNAI1, SNAI2, and ZEB1 with that of DCBLD2 were performed in 515 LUAD tissues from the TCGA database. The data revealed that DCBLD2 expression was positively correlated with the expression of all 4 core EMT TFs (Spearman’s r = 0.287, 0.214, 0.349, and 0.229, respectively; *p* < 0.0001) (Figure S3h). Collectively, these observations indicated that DCBLD2 enhanced EMT in vitro and in vivo.

### 3.6. Cisplatin Promotes EMT, Cell Migration, and Metastasis through DCBLD2

To explore whether cisplatin promoted EMT, cell migration, and metastasis via DCBLD2, we transfected A549/cis and Pc9/cis cell lines with shDCBLD2 to stably knock down DCBLD2 (Figure S4a).

**Figure 4:**
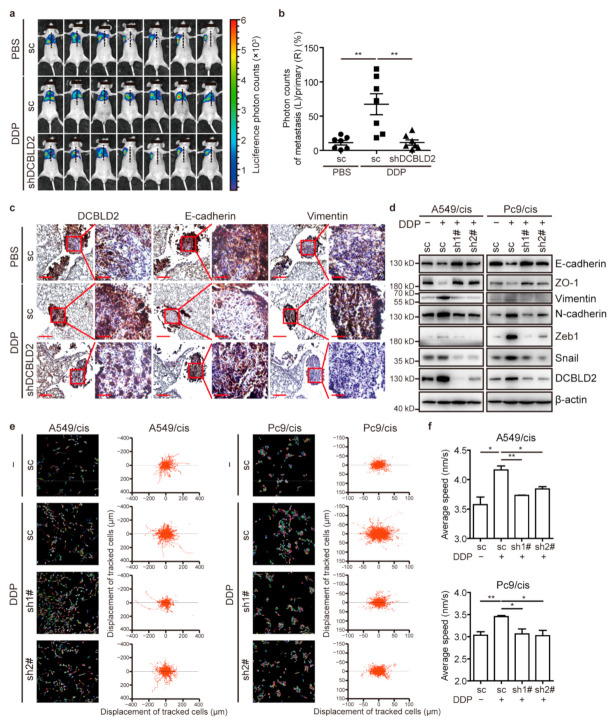
DCBLD2 mediates cisplatin-induced EMT and tumor metastasis. (**a**,**b**) A total of 1 × 10^6^ Pc9/cis-luciferase cells transfected with shDCBLD2 or scramble control were inoculated into the right lungs of BALB/c nude mice (*n* = 7 per group). On day 7, mice were intraperitoneally injected with 4.0 mg/kg cisplatin or an equivalent volume of PBS every 7 days. Thirty-five days after inoculation, mice underwent a bioluminescence test and were sacrificed. The primary tumors in the right lung and the metastatic foci in the left lung were subjected to HE staining or IHC staining. Bioluminescence imaging is shown in (**a**), and quantitation of bioluminescence signals is shown in (**b**). (**c**) Representative IHC images of E-cadherin and Vimentin expression in paraffin-embedded serial sections of the primary lesion (right lung) of orthotopically implanted mice from Figure 3A. Scale bar: 200 μm (left) and 50 μm (right). (**d**) Western blot assay to confirm the effect of DCBLD2 on EMT in cisplatin treatment. Cells with stable DCBLD2 knockdown or scramble control were cultured in the presence or absence of 2 μg/mL cisplatin for 24 h. (**e**,**f**) Dynamic imaging analyses of cell migration in stable DCBLD2 knockdown cells and scramble control cells pretreated with 2 μg/mL cisplatin or vehicle control for 24 h. Cell migration was measured over 16 h by HCS. Representative images recording the single-cell motion track (left panel) and single-cell ultimate displacement (right panel) are shown in (**e**), and the average speed was measured by Harmony in (**f**). * *p* < 0.05, ** *p* < 0.01, *t*-test. DDP, refers to cis-diamminedichloridoplatinum (II), also known as cisplatin or cisplatinum.

The effect of DCBLD2 on cisplatin-induced metastasis was evaluated in the orthotopic xenograft model. The number of metastatic foci in nude mice orthotopically injected with cells transduced with scramble control was dramatically increased in tumor-bearing mice regularly treated with cisplatin. However, nude mice injected with shDCBLD2 cells had fewer metastatic foci after cisplatin treatment, indicating that knocking down DCBLD2 reversed the cisplatin-induced increase in metastasis in vivo (Figure 4a,b).

Furthermore, we detected the expression of E-cadherin and Vimentin in primary tumor tissues from orthotopic xenograft model mice. Tumor-bearing mice treated with cisplatin showed decreased level of E-cadherin and elevated level of Vimentin, while nude mice inoculated with shDCBLD2 cells showed a reduction in cisplatin-induced EMT (Figure 4c and Figure S4b). In vitro, western blot assays indicated that knockdown of DCBLD2 in A549/cis and Pc9/cis cells significantly inhibited the cisplatin-mediated increase in the expression mesenchymal markers (Vimentin and N-cadherin) and core EMT TFs (Zeb1 and Snail) while restoring the expression of epithelial markers (E-cadherin and ZO-1) (Figure 4d). Consistent with the western blot results, the IF assays exhibited the same tendencies (Figure S4c).

Regarding the role of DCBLD2 in cisplatin-induced cell migration, the HCS results showed that knockdown of DCBLD2 prominently ameliorated the chaotic pattern of cell movement tracks and significantly reduced the migration speed of cells treated with cisplatin (Figure 4e,f). The same phenomena were also observed in Transwell and wound healing assays (Figure S4d–g). We also showed that knockdown of DCBLD2 had no effect on the response of resistant cells to cisplatin, precluding the possibility of reduced migratory ability and suppressed tumor metastasis due to tumor cell death (Figure S4h). Collectively, we demonstrated that DCBLD2 is essential for cisplatin-induced EMT, migration, and metastasis in LUAD.

### 3.7. DCBLD2 Potentiates EMT and Cell Migration via GSK3β/β-Catenin

**Figure 5:**
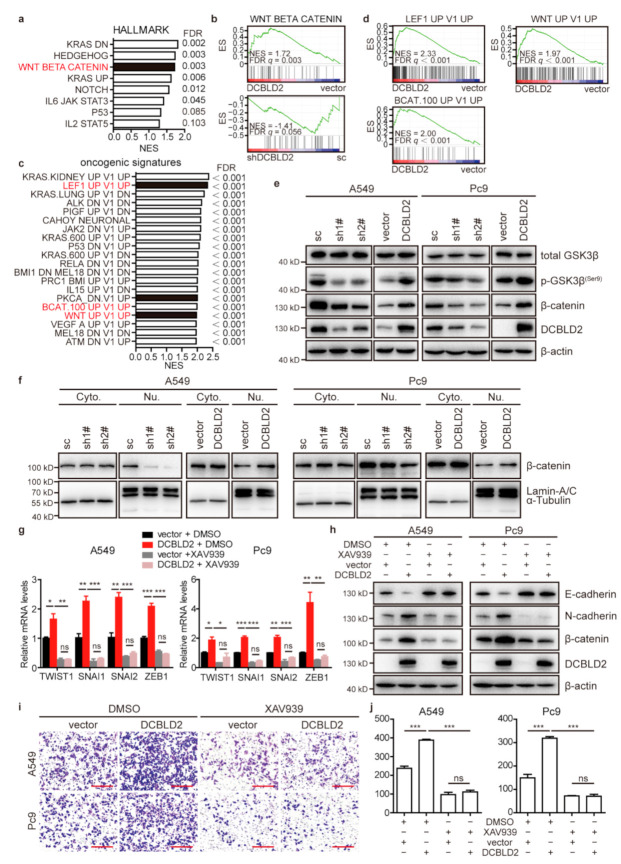
The Wnt/β-catenin signaling pathway mediates DCBLD2-induced metastasis in LUAD. (**a**,**b**) Hallmark enrichment classification of signaling pathways of differentially expressed genes in empty vector and DCBLD2-overxpressing A549 cells by GSEA. (**c**,**d**) Oncogenic signature enrichment classification of differential genes by GSEA. (**e**) The expression of various Wnt/β-catenin signaling pathway proteins was evaluated by western blot assay in A549 and Pc9 cells. (**f**) Western blot assay of β-catenin expression in A549 and Pc9 cells after nuclear-cytoplasmic fractionation. α-Tubulin and Lamin A/C were used as cytoplasmic and nuclear loading controls, respectively. (**g**) RT-PCR assays of the expression of EMT TFs in empty vector and DCBLD2-transfected cells after treatment with 20 μM XAV939 or DMSO for 48 h. (**h**) Western blot assay of the expression of E-cadherin and N-cadherin in empty vector and DCBLD2-transfected cells treated with 20 μM XAV939 or DMSO for 48 h. (**i**,**j**) Transwell assay showing the effect of β-catenin on DCBLD2-induced cell migration. Empty vector and DCBLD2-transfected cells were pretreated with 20 μM XAV939 or DMSO for 48 h and then subjected to Transwell assays. Images of the cells on the underside of the membrane of wells are shown in (i) after culture for 8 h in the presence of a chemoattractant. Scale bar: 200 μm. Quantification of migrated cells is depicted in (j). * *p* < 0.05, ** *p* < 0.01, *** *p* < 0.001, *t*-test.

To investigate the mechanism underlying DCBLD2-driven EMT and metastatic events, a transcriptome analysis and GSEA were performed in A549 cells transfected with DCBLD2 or empty vector. Hallmark or oncogenic signature analysis revealed multiple cancerous signaling pathways, especially signature genes enriched in the Wnt/β-catenin signaling pathway (Figure 5a–d).

To confirm whether DCBLD2 can influence the Wnt/β-catenin signaling pathway, we measured the phosphorylation of GSK3β and β-catenin in A549 and Pc9 cells by western blot. The results showed that GSK3β was phosphorylated at the serine 9 residue by DCBLD2, resulting in the inactivation of GSK3β and the accumulation of β-catenin (Figure 5e). Furthermore, DCBLD2 enhanced the nuclear localization of β-catenin, as determined by nuclear-cytoplasmic fractionation assay (Figure 5f), which was further confirmed by IF staining results (Figure S5a,b).

We further confirmed that DCBLD2 promotes EMT and migration via GSK3β/β-catenin. Upon transfection of LUAD cell lines with DCBLD2 and treatment with XAV-939 (CAS No. 284028-89-3), a β-catenin inhibitor, we showed that DCBLD2 increased the expression of the core EMT-related TFs TWIST1, SNAI1, SNAI2, and ZEB1 and that this elevated expression was robustly inhibited by XAV-939, as shown in the RT-PCR assay (Figure 5g). Likewise, western blot analysis showed that the expression of β-catenin and N-cadherin was profoundly blocked and E-cadherin was increased in DCBLD2-transfected cells when treated with XAV-939 (Figure 5h). XAV-939 also sufficiently inhibited DCBLD2-promoted cell migration (Figure 5i,j). In the complementation test, we treated cells with CHIR-99021 (CAS No. 252917-06-9, a GSK3β inhibitor) to serve as a potent agonist of β-catenin. The data revealed that knockdown of DCBLD2 significantly suppressed the expression of the core EMT TFs, but their expression was notably increased in cells treated with CHIR-99021 (Figure S5c). The expression of N-cadherin was prominently enhanced while that of E-cadherin was markedly inhibited in DCBLD2-knockdown cells treated with CHIR-99021 compared to those without CHIR-99021 treatment (Figure S5d). Additionally, CHIR-99021 sufficiently restored cell migration in cells with DCBLD2 knockdown (Figure S5e,f).

Similarly, in cisplatin-resistant A549 and Pc9 cells, knocking down DCBLD2 reduced the phosphorylation of GSK3β and the expression of β-catenin (Figure S6a). In addition, knock down of DCBLD2 inhibited the translocation of β-catenin to the nucleus, as shown by western blot and IF staining (Figure S6b–d). CHIR-99021 significantly rescued the expression of EMT TFs and the mesenchymal phenotype impaired by DCBLD2 knockdown (Figure S6e,f). The suppressed migration mediated by DCBLD2 knockdown was also markedly restored by CHIR-99021 treatment (Figure S6g,h).

Therefore, we proved that DCBLD2 mediated GSK3β phosphorylation, which inactivated GSK3β and led to the disassembly of the destruction complex and the stabilization of β-catenin. Accumulated β-catenin was translocated to the nucleus, where it activated the transcription of EMT genes, such as TWIST1, SNAI1, SNAI2 and ZEB1, and consequently promoted metastasis.

### 3.8. Cisplatin Upregulates DCBLD2 Expression via the ERK/AP-1 Axis

**Figure 6:**
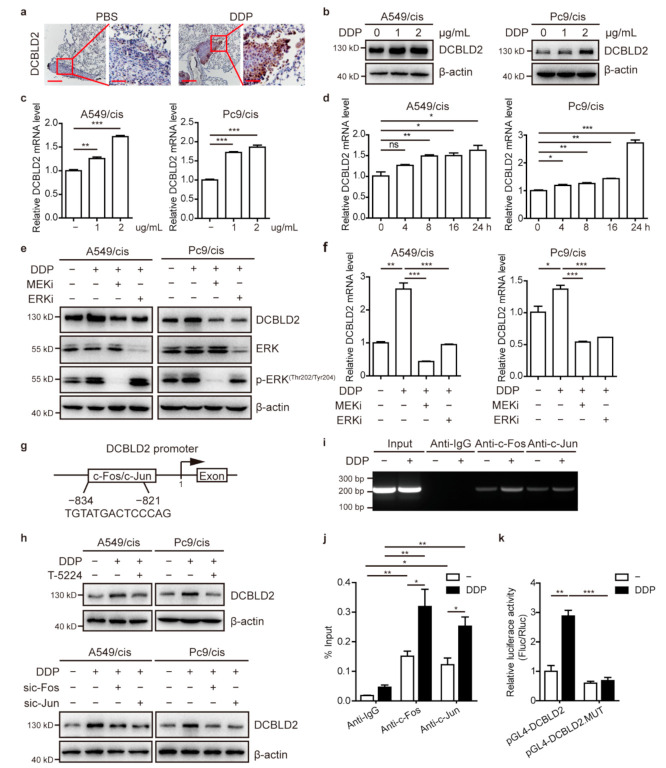
Cisplatin upregulates DCBLD2 expression by the ERK/AP-1 signaling pathway. (**a**) Representative IHC images of DCBLD2 expression in primary tumor tissues from Figure 1a. Scale bar: 200 μm (left), 50 μm (right). (**b**,**c**) Western blot assays (**b**) and RT-PCR assays (**c**) of DCBLD2 expression in A549/cis and Pc9/cis cells cultured in the absence or presence of either 1 or 2 μg/mL cisplatin for 24 h. (**d**) RT-PCR assays of DCBLD2 expression in A549/cis and Pc9/cis cells treated with 2 μg/mL cisplatin for 4, 8, 16, or 24 h. (**e**,**f**) Western blot assays (**e**) and RT-PCR assays (**f**) were performed to detect the expression of DCBLD2 in A549/cis and Pc9/cis cells with or without pretreatment with MEK/ERK inhibitors for 12 h and cultured in 2 μg/mL cisplatin for another 24 h. (**g**) Schematic of the binding sites of the TF AP-1 (c-Fos/c-Jun heterodimer) in the DCBLD2 promoter. (**h**) Western blot assay was performed to detect the effect of AP-1 on DCBLD2 expression in A549/cis and Pc9/cis cells. Upper panel: A549/cis and Pc9/cis cells were cultured in the presence or absence of T-5224 for 12 h before they were treated with 2 μg/mL cisplatin for another 24 h and then subjected to western blot assay. Lower panel: cells with or without c-Fos or c-Jun siRNA treatment for 8 h were then cultured with 2 μg/mL cisplatin for another 24 h and subjected to western blot assay. (**i**,**j**) The regulation of DCBLD2 expression by AP-1 was analyzed by ChIP PCR (**i**) and qPCR (**j**) in A549/cis cells after stimulation with cisplatin for 24 h. Chromatin was immunoprecipitated with anti-c-Fos and anti-c-Jun antibodies and then subjected to PCR/qPCR analysis. (**k**) Luciferase analysis of A549/cis cells. A549/cis cells were transfected with pGL4-DCBLD2-promoter or pGL4-DCBLD2-promoter-mutation. After transfection for 48 h and treatment with cisplatin or vehicle control for 24 h, cells were subjected to dual-luciferase analysis. Results were expressed as fold induction relative to the cells transfected with pGL4-DCBLD2-promoter and treated with vehicle after normalization to Renilla activity. * *p* < 0.05, ** *p* < 0.01, *** *p* < 0.001, *t*-test. DDP, refers to cis-diamminedichloridoplatinum (II), also known as cisplatin or cisplatinum.

Interestingly, we found that DCBLD2 expression in primary LUAD tissues was significantly elevated in mice treated with cisplatin compared to those without cisplatin treatment (Figure 6a). To confirm the upregulated expression of DCBLD2 by cisplatin, we used western blot and RT-PCR to measure DCBLD2 expression after cisplatin treatment. The results showed that DCBLD2 expression was notably increased in a dose- and time-dependent manner (Figure 6b–d).

Next, we explored the mechanism underlying cisplatin-mediated DCBLD2 overexpression in resistant LUAD cells. It has been reported that cisplatin causes prolonged induction of ERK1/2 activity in a dose-dependent manner [24,25]; therefore, we pretreated cisplatin-resistant cell lines with Trametinib (a MEK inhibitor, CAS No. 871700-17-3) or SCH772984 (a ERK inhibitor, CAS No. 942183-80-4) for 12 h followed by treatment with 2 μg/mL cisplatin for another 24 h. Our western blot and RT-PCR data showed that DCBLD2 expression was reduced when cells were pretreated with MEK or ERK inhibitor (Figure 6e,f).

Then, we checked the promoter region of the human DCBLD2 gene and found that activator protein-1 (AP-1, c-Fos/c-Jun heterodimer), which is a TF downstream of ERK, could bind to the DCBLD2 promoter through TF binding site prediction (Figure 6g). To determine whether c-Fos and c-Jun transcriptionally regulate DCBLD2 expression, cisplatin-resistant LUAD cells were either pretreated with T-5224 (CAS No. 530141-72-1), an AP-1 inhibitor (Figure 6h, top panel), or transfected with siRNA targeting c-Fos or c-Jun (Figure 6h, bottom panel). Cisplatin significantly increased DCBLD2 expression, while pretreatment with AP-1 inhibitor or knockdown of c-Fos or c-Jun greatly inhibited the cisplatin-mediated increase of DCBLD2 expression. ChIP assays were performed in A549/cis cells cultured in the presence or absence of cisplatin. In DNA fractions pulled down with an anti-c-Fos and anti-c-Jun antibody, a binding element located at −834 to −821 was detected when treated with cisplatin, suggesting that AP-1 can bind to the promoter of DCBLD2 (Figure 6i,j).

To determine whether cisplatin activates the transcription of DCBLD2, we constructed a full-length DCBLD2 luciferase promoter vector and transfected this reporter into A549/cis cells. Luciferase analysis showed that cisplatin substantially increased the transcriptional activity of the DCBLD2 promoter. To determine whether the binding site located at −834 to −821 is necessary for AP-1 to transactivate the DCBLD2 promoter, we transformed this binding site by deletion mutation. The mutation of the binding site almost abrogated the cisplatin-induced activation of the DCCLD2 promoter (Figure 6k).

Taken together, these data suggest that ERK1/2 can be activated by cisplatin, leading to binding of AP-1 to the promoter of DCBLD2 and the subsequent upregulation of DCBLD2 expression.

### 3.9. DCBLD2-Targeted Therapy Strikingly Inhibits Tumor Metastasis in vivo

**Figure 7:**
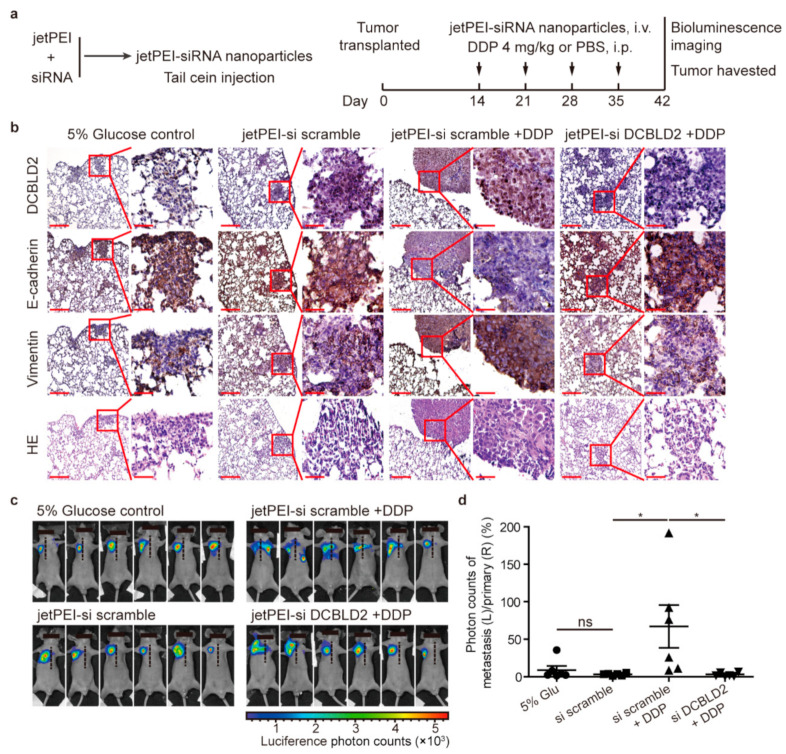
jetPEI nanocarrier-delivered anti-DCBLD2 siRNAs significantly inhibit tumor metastasis in a resistant LUAD mouse model. (**a**) jetPEI nanocarrier was used as the vehicle for DCBLD2-targeted siRNAs to inhibit DCBLD2 expression in vivo. Schematic of the nanoparticles (left panel); the timeline of drug administration (right panel). Pc9/cis-luciferase cells were orthotopically transplanted into nude mice to develop tumors (*n* = 6 for each group). Fourteen days after inoculation, jetPEI-nanoparticles containing scramble or DCBLD2-targeted siRNA were intravenously injected. Mice injected with 5% glucose served as a negative control group. Mice were intraperitoneally injected with 4.0 mg/kg cisplatin or an equivalent volume of PBS every 7 days. (**b**) The primary tumors in the right lung and the metastatic foci in the left lung were serially sectioned and then subjected to HE staining. The expression of DCBLD2, E-cadherin, and Vimentin was detected by IHC staining. Scale bar: 200 μm (left), 50 μm (right). (**c**,**d**) Bioluminescence imaging (**c**) was performed to detect metastasis, and quantitation of bioluminescence signals is shown in (**d**). * *p* < 0.05, *t*-test. DDP, refers to cis-diamminedichloridoplatinum (II), also known as cisplatin or cisplatinum.

To further investigate the anti-metastatic effects of DCBLD2-targeted therapy *in vivo*, we used a jetPEI nanocarrier as the vehicle for DCBLD2-specific siRNAs to inhibit DCBLD2 expression in tumor-bearing mice (Figure 7a). The IHC staining data showed that the jetPEI-delivered DCBLD2 siRNA efficiently inhibited DCBLD2 expression, which blocked EMT when mice were treated with cisplatin, leading to increased E-cadherin and decreased Vimentin expression in tumor tissues (Figure 7b). Therefore, jetPEI-siDCBLD2 nanoparticles resulted in a remarkable decrease in the number of metastatic foci during cisplatin therapy (Figure 7c,d), suggesting that DCBLD2 is a promising therapeutic target for anti-metastatic therapy.

## 4. Discussion

Chemotherapy is currently one of the most effective treatments for solid tumors [26,27,28]. Although chemotherapy is commonly used as the only treatment for advanced LUAD, many patients also receive cisplatin-based chemotherapy in conjunction with surgery, radiotherapy, or biological therapy as an adjuvant or neoadjuvant chemotherapy [29]. As a systemic treatment, chemotherapy effectively suppresses tumor growth via cytotoxicity, but it is also possible that chemotherapy can either objectively screen resistant and highly aggressive cells or enhance the metastatic potential of surviving cells, thereby promoting migration and increasing the risk of metastatic disease [30]. Wang [31] reported that chemotherapy promotes tumor invasion and metastasis in a human lung cancer xenograft model. Other studies have also shown that chemotherapy can promote the accelerated regrowth and metastasis of non-small cell lung cancer by promoting M2 polarization of tumor-associated macrophages or stimulating mesenchymal stem cells [32,33,34]. However, previous studies and clinical treatments have paid less attention to patients with chemotherapy-resistant and metastatic tumors. The chemotherapy-induced metastasis models used in previous studies, including intravenous injection of tumor cells to mimic tumor metastasis, treatment of tumor cell lines ex vivo, the use of chemotherapy-sensitive cell lines that ignore tumor heterogeneity, and even the selection of tumor models that are not suitable for chemotherapy, cannot effectively simulate the disease state of such patients. Here, we used an orthotopic transplantation model with cisplatin-resistant LUAD cells to simulate the disease status of these refractory patients. Based on our results, exposure of cisplatin-resistant cells to chemotherapeutic agents enhanced the migratory and metastatic potential of surviving cells in vitro and in vivo.

Therefore, we conducted a bioinformatics analysis to screen candidate genes that might mediate chemotherapy-induced metastasis in LUAD and identified DCBLD2 as a critical regulator in cisplatin-induced EMT and metastasis. We chose DCBLD2 rather than the other two genes, not only because DCBLD2 ranked first in the short list of candidate genes. However, SLC6A8 is a plasma membrane protein involved in the creatine metabolic process and transmembrane transport [35]. No report supported SLC6A8 was related to tumor or metastasis, and SLC6A8 is not significantly enriched in gene sets related to cell motility, adhesion or EMT by GSEA. Although some evidence supported that PHGDH enhanced proliferation, migration and invasion of pancreatic cancer [36], and contributed to the resistance of sorafenib and other tyrosine kinase inhibitors in hepatocellular carcinoma [37]. However, PHGDH is not particularly highly expressed in LUAD cells and tissues. We demonstrated that DCBLD2 was widely and highly expressed in LUAD and that DCBLD2 expression was significantly higher in LUAD tissues than in adjacent nontumorous tissues. DCBLD2 overexpression has also been reported in glioblastomas, hypopharyngeal squamous cell carcinoma, and invasive myxofibrosarcoma; however, its expression is inconsistent in different tumors. DCBLD2 expression is downregulated in colorectal cancer [38], gastric cancer [39], melanomas [40] and neuroendocrine cancer [41]. Clinically, in patients with LUAD, high levels of DCBLD2 correlated with advanced tumor staging, nodal involvement, and poor survival and were shown to be a prognostic predictor.

Moreover, we proved that DCBLD2 promotes metastasis by EMT through various lines of evidence, including stably transfected cell lines, orthotopic xenograft models, and clinical specimens. Our data suggested that cell migration was observably promoted by DCBLD2 in vitro based on cell migration analyses, including HCS, Transwell, and wound healing assays. DCBLD2 prominently facilitated metastasis in vivo, as others have described before [16,18,42]. EMT has been shown to play critical roles in promoting metastasis and invasion as well as to contribute to drug resistance in carcinoma. In this study, western blots and IF assays showed that DCBLD2 facilitates EMT progression in LUAD cells. IHC staining of consecutive sections of orthotopically transplanted tumors in mice and clinical LUAD specimens indicated a strong correlation between DCBLD2 expression and epithelial/mesenchymal marker levels.

Furthermore, we confirmed that DCBLD2 is critical for EMT, cell migration, and metastasis under cisplatin treatment. There is ample evidence that chemotherapy induces tissue repair procedures, causing changes in the phenotype and active signaling pathways of tumor cells and other types of cells. These changes include the mobilization of bone marrow progenitor cells, enhancement of EMT and proinflammatory response, acquisition of chemoresistance, and increased prometastatic properties of circulating tumor cells and play major roles in tumorigenic activities [43,44]. Our data indicated that enhanced EMT and cell migration of cells exposed to cisplatin chemotherapy were significantly blocked by knocking down DCBLD2; this knockdown additionally inhibited EMT of the primary tumor and reduced the cisplatin-mediated formation of metastatic foci in vivo, which proved that cisplatin promotes EMT and metastasis through DCBLD2.

Mechanistically, we demonstrated that DCBLD2 promotes EMT and migration via the Wnt/β-catenin signaling pathway. Numerous investigations have proposed the Wnt/β-catenin pathway as one of the most crucial signaling pathways in the progression and metastasis of various tumors with stemness and EMT phenotypes [45,46,47]. In the Wnt/β-catenin pathway, β-catenin is phosphorylated by CK1 and GSK3β in the cytoplasmic β-catenin destruction complex, leading to its ubiquitination by E3 ligase and consequent degradation in the proteasome. When the Wnt/β-catenin signaling is activated, the destruction complex is recruited to the membrane, resulting in the stabilization of β-catenin [48,49]. In this study, we found that DCBLD2 phosphorylates serine 9 of GSK3β, thereby inhibiting its activity and protecting β-catenin from degradation. Accumulated β-catenin is translocated to the nucleus and activates the transcription of EMT-related genes. These observations strongly support the notion that DCBLD2 promotes EMT and metastasis via the Wnt/β-catenin signaling pathway in LUAD.

Our findings suggested that cisplatin upregulates DCBLD2 expression via the ERK/AP-1 axis. Numerous studies have indicated that cisplatin activates the MAPK/ERK signaling pathway in liver cancer, breast cancer, cervical cancer and osteosarcoma [50,51,52,53]. DNA damage induced by cisplatin causes activation of the Ras signaling cascade. One of the key downstream steps after activation of ERK is activation of AP-1, which regulates the activity of genes directly involved in or related to DNA repair and contributes to promoting cell survival [54,55,56]. Our mechanistic studies revealed that MEK or ERK inhibitors notably blocked cisplatin-mediated increases in DCBLD2 expression, and the significantly elevated DCBLD2 expression under cisplatin treatment was mainly mediated by AP-1, which directly binds to the promoter of DCBLD2. Importantly, as AP-1 is a TF in ERK signaling, blocking AP-1 with siRNAs or inhibitors dramatically reduced DCBLD2 protein levels in cisplatin-resistant cell lines. Therefore, the expression levels of DCBLD2 or activation of ERK in LUAD patients during adjuvant chemotherapy or neoadjuvant chemotherapy might be a checkpoint in preventing chemotherapy-induced metastasis.

## 5. Conclusions

In clinical practice, patients often receive consolidation chemotherapy to reduce metastasis or recurrence caused by residual tumor cells after responding to sequential induction chemotherapy. In this work, we revealed that the continuous exposure of resistant cells to chemotherapy agents might induce EMT and increase the metastatic potential of remaining cells. DCBLD2 promotes EMT and metastasis by phosphorylating GSK3β and stabilizing β-catenin. The application of cisplatin further upregulates the expression of DCBLD2 via ERK/AP-1 axis and facilitates tumor metastasis. Moreover, DCBLD2-targeted siRNA encapsulated by jetPEI nanocarriers significantly inhibited DCBLD2 expression and tumor metastasis in vivo during cisplatin treatment. Our results suggested that blocking DCBLD2 might play a preventive role in platinum chemotherapy-induced metastasis.

## Figures and Tables

**Table 1:** Association between DCBLD2 mRNA expression and clinicopathological features of patients with LUAD from the TCGA database. Cut off: 12.9 FPKM, * *p* < 0.05, ** *p* < 0.01, Chi-square test.

Characteristics	Total (*n* = 513)	DCBLD2 Expression		χ^2^	*p* Value
		Low (*n* = 339)	High (*n* = 114)		
Gender				1.475	0.225
Male	222	167	55		
Female	291	232	59		
Age(year)				0.323	0.570
<65	237	187	50		
≥65	276	212	64		
T staging				1.679	0.195
T1–T2	446	351	95		
T3–T4	67	48	19		
Nodal staging				7.360	0.007 **
N0	338	275	63		
N1–N3	175	124	51		
M staging				0.589	0.443
M0	488	378	110		
M1	25	21	4		
TNM				6.063	0.014 *
I	277	227	50		
II,III,IV	236	172	64		

**Table 2:** Association between DCBLD2 expression and clinicopathological features of patients with LUAD from Tianjin Medical University Cancer Institute and Hospital. Cut off: H score = 5, * *p* < 0.05, ** *p* < 0.01, Chi-square test, except for #: Fisher test.

Characteristics	Total (*n* = 125)	DCBLD2 Expression		χ^2^	*p* Value
		Low (*n* = 37)	High (*n* = 88)		
Gender				1.926	0.165
Male	59	21	38		
Female	66	16	50		
Age (year)				0.826	0.363
<65	91	29	62		
≥65	34	8	26		
T staging				0.409	0.523
T1	52	17	35		
T2–T4	73	20	53		
Nodal staging				7.119	0.008 **
N0–N1	83	31	52		
N2–N3	42	6	36		
M staging					1.000 #
M0	124	37	87		
M1	1	0	1		
TNM				6.406	0.011 *
I	56	23	33		
II,III,IV	69	14	55		

## Data Availability

The TCGA data referenced in the study are available in a public repository from TCGA website (http://cancergenome.nih.gov/, accessed on 2 December 2017). RNA-Seq data are available at the Gene Expression Omnibus (GEO) repository, the accession number is GSE168498.

## Supplementary Materials

The following are available online at https://www.mdpi.com/2072-6694/13/6/1403/s1, Figure S1: Cisplatin promotes cell migration and distant metastasis in chemotherapy-resistant LUAD, Figure S2: DCBLD2 induces cell migration in LUAD, Figure S3: DCBLD2 facilitates EMT in LUAD, Figure S4: DCBLD2 mediates cisplatin-induced EMT and migration, Figure S5: DCBLD2 modulates EMT via the Wnt/β-catenin signaling pathway in LUAD, Figure S6: DCBLD2 modulates EMT via the Wnt/β-catenin signaling pathway in cisplatin-resistant LUAD. Figure S7: Uncropped Western blot images.
