# Supplementary material for: DCBLD2 Mediates Epithelial-Mesenchymal Transition-Induced Metastasis by Cisplatin in Lung Adenocarcinoma
Source: Cancers (Basel). 2021 Mar 19;13(6):1403. doi: 10.3390/cancers13061403 (PMC8003509; doi:10.3390/cancers13061403)

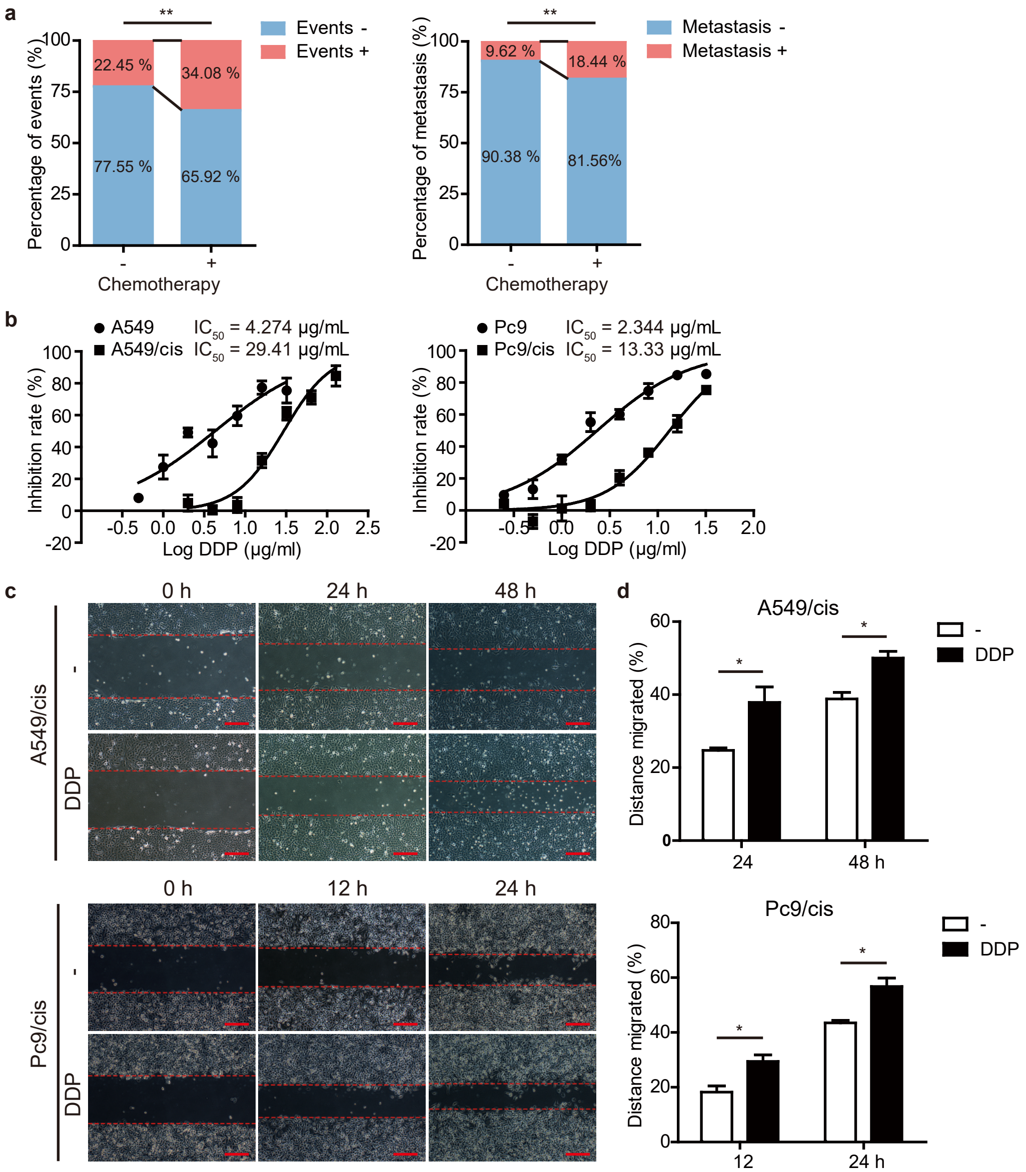

Figure S1. Cisplatin promotes cell migration and distant metastasis in chemotherapy-resistant LUAD. **(a)** The proportion of long-term events and distant metastasis after chemotherapy in LUAD patients in the TCGA database. Events include distant metastasis, locoregional recurrence, and new primary tumors. ** $P < 0.01$, chi-square test. **(b)** A549/cis and Pc9/cis cells were treated with cisplatin for 24 hours to analyze their sensitivity to cisplatin. **(c-d)** Wound healing assay was performed to detect the effect of cisplatin on cell migration in A549/cis and Pc9/cis cells. Cells were pretreated with 2 μg/mL cisplatin or vehicle control for 24 hours and then subjected to wound healing assays. Images recording cell distribution at 0, 12, and 24 hours after the formation of the wound are shown in (c). Scale bar: 200 μm. Quantification of migrated distance is depicted in (d), * $P < 0.05$, t-test.

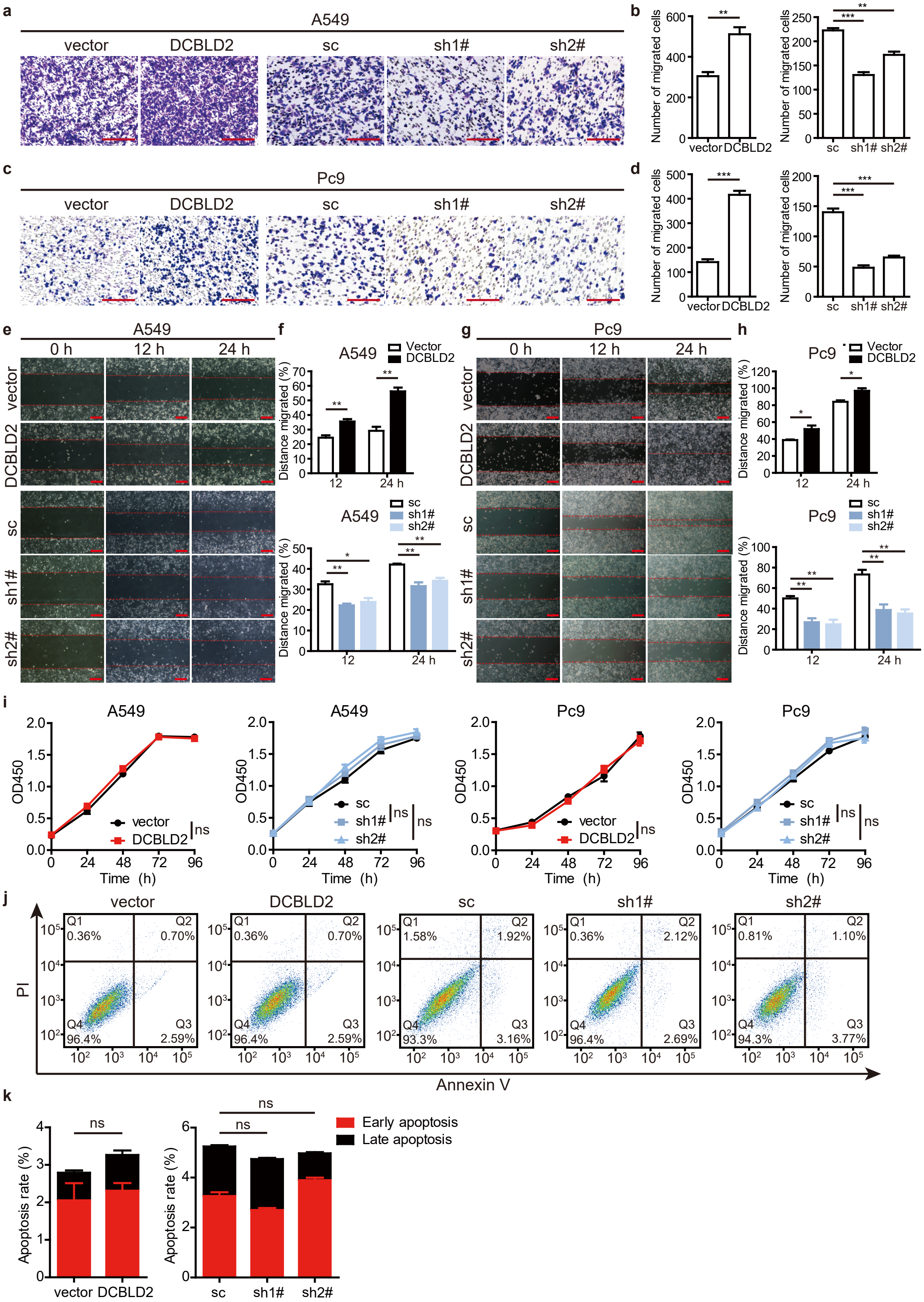

Figure S2. DCBLD2 induces cell migration in LUAD. **(a-d)** Migration of A549 (a-b) and Pc9 (c-d) cells by Transwell assay. Images of the cells on the underside of the membrane of wells are shown in (a) and (c) after incubation for 8 hours in the presence of a chemoattractant. Quantification of migrated cells is depicted in (b) and (d), ** $P < 0.01$, *** $P < 0.001$, t-test. **(e-h)** Migration of A549 (e-f) and Pc9 (g-h) cells by wound healing assay. Images recording cell distribution immediately after the formation of the wound and 12 hours or 24 hours after scratching are shown in (e) and (g). Scale bar: 200 μm. Quantification of migrated distance is depicted in (f) and (h), * $P < 0.05$, ** $P < 0.01$, t-test. **(i)** Monolayer growth rates of DCBLD2-overexpressing or shDCBLD2-transfected A549 and Pc9 cells were determined by the CCK-8 assay. ns, $P > 0.05$, two-way ANOVA test. **(j-k)** The apoptosis rate of DCBLD2-overexpressing or shDCBLD2-transfected A549 cells as measured by FACS. Quantification of the early and late apoptosis rates is depicted in (k), ns, $P > 0.05$, two-way ANOVA.

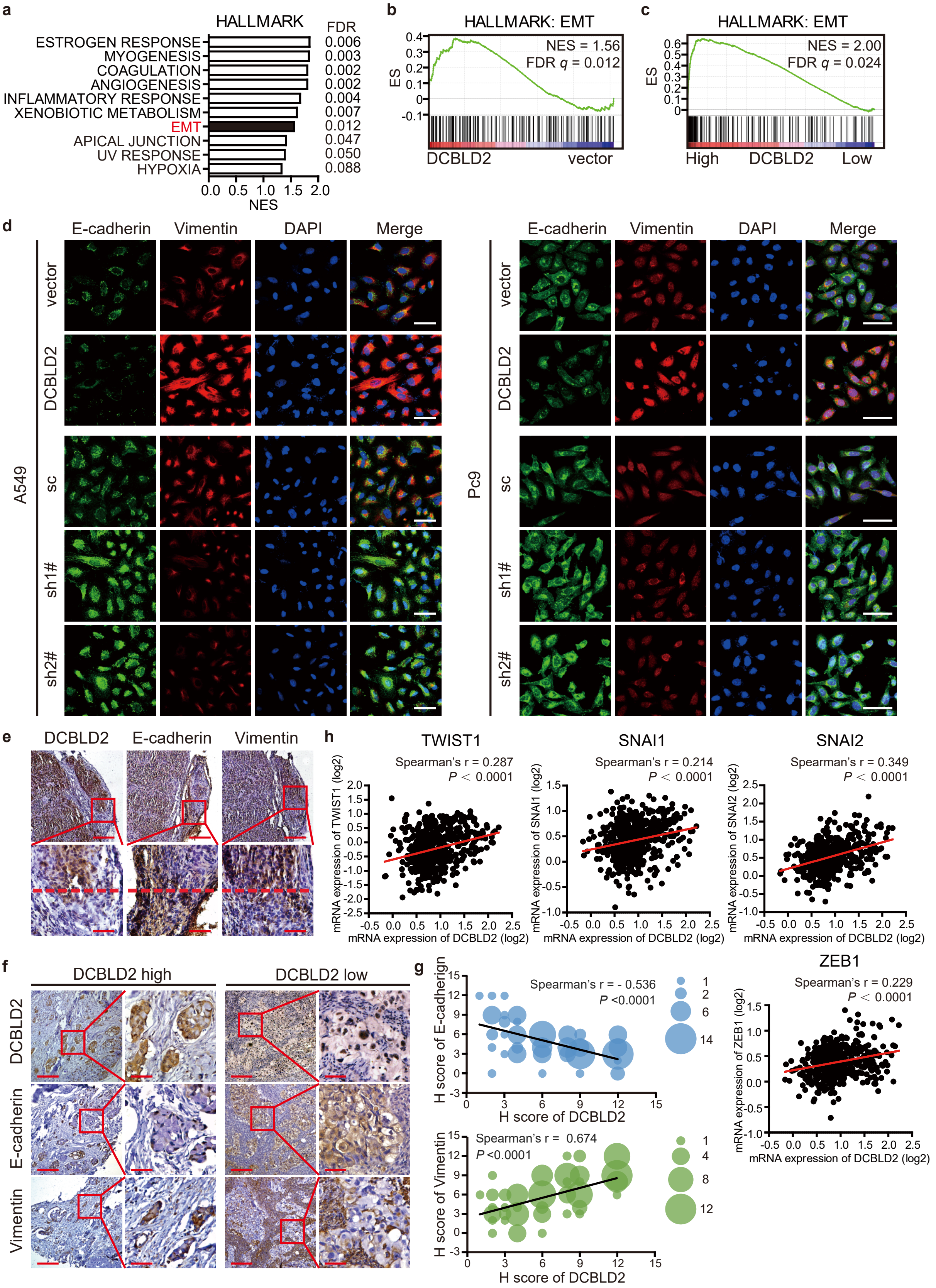

Figure S3. DCBLD2 facilitates EMT in LUAD. **(a-b)** Hallmark enrichment classification of phenotypes of differentially expressed genes in empty vector and DCBLD2-transfected A549 cells by GSEA. **(c)** GSEA of LUAD patients from the TCGA database according to different DCBLD2 levels. The cutoff value of DCBLD2 was 12.86 RPKM. NES = 2.00, FDR q = 0.024. **(d)** IF images of EMT markers and DAPI in A549 and Pc9 cells. Scale bar: 50 μm. **(e)** Representative IHC images of the expression of E-cadherin and Vimentin in subcutaneous LUAD tissues with differential DCBLD2 expression. A549 cells transfected with empty vector or DCBLD2-overexpressing vector were mixed at a ratio of 1:1 and subcutaneously implanted in BALB/c nude mice to form heterogeneous tumor tissues with different expression levels of DCBLD2. **(f-g)** Representative IHC images (f) and statistical analysis (g) of EMT marker expression in 122 LUAD tumor tissues. Serial paraffin-embedded sections of a tissue array from 122 LUAD patients were stained for EMT markers. Then, the expression of E-cadherin or Vimentin was evaluated by H scores ranging from 0 to 12. Scale bar: 200 μm (left), 50 μm (right). (g) shows the correlation analysis of E-cadherin (upper panel) or Vimentin (lower panel) expression with DCBLD2 by Spearman's r test, $P < 0.0001$. **(h)** Correlation analysis of the mRNA expression of TWIST1, SNAI1, SNAI2, and ZEB1 with that of DCBLD2 by Spearman's r test in 515 LUAD tissues from the TCGA database.

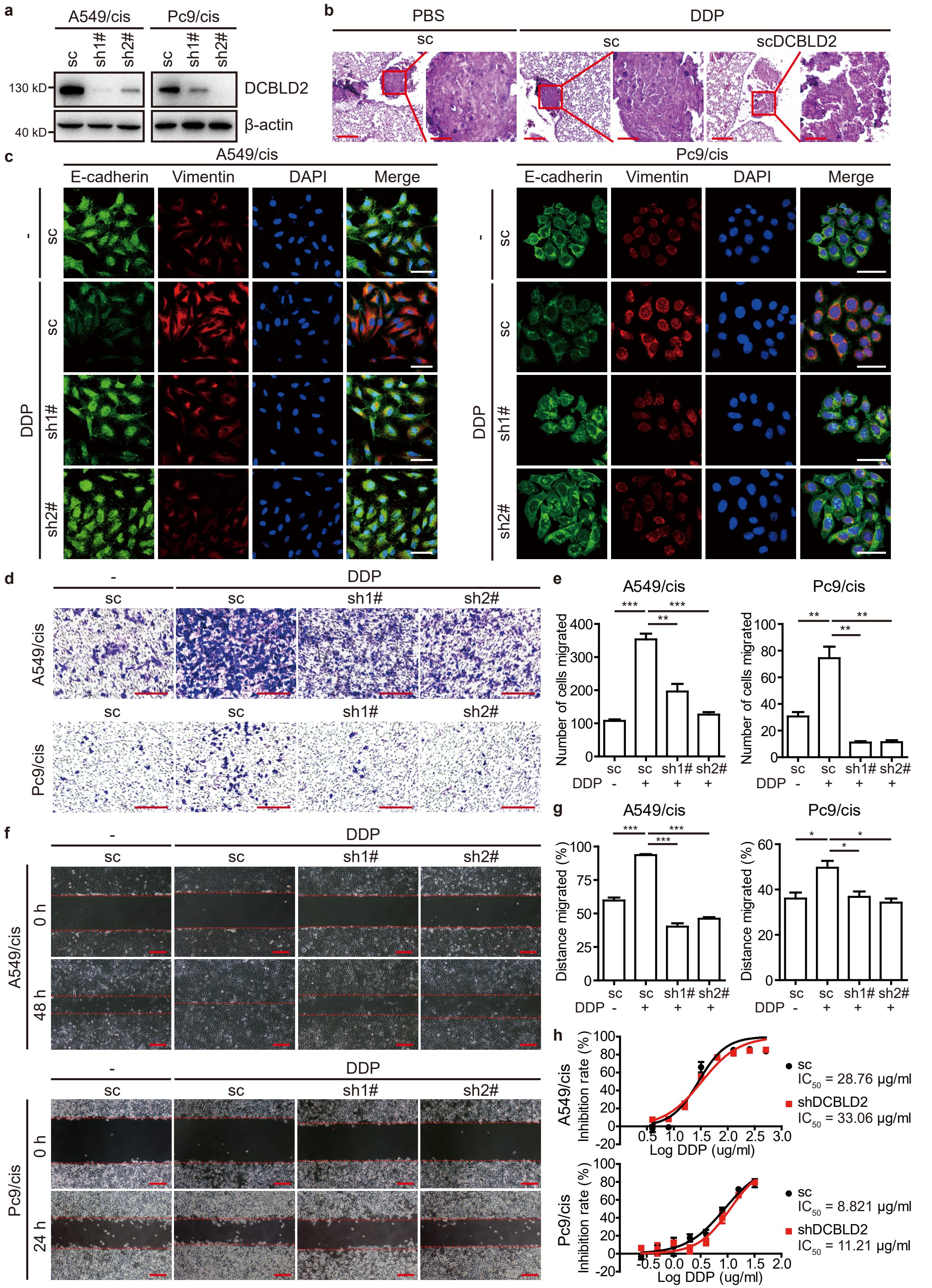

Figure S4. DCBLD2 mediates cisplatin-induced EMT and migration. **(a)** Cells were transfected with lentiviruses to downregulate DCBLD2 expression. Western blot assays were performed to verify the regulatory effect of DCBLD2 in A549/cis and Pc9/cis cells. **(b)** Representative HE images of paraffin-embedded sections of primary tumor tissues from the right lung of mice receiving orthotopic implantation from Figure 4a. Scale bar: 200 μm (left) and 50 μm (right). **(c)** Representative IF images of EMT markers and DAPI staining. Stable DCBLD2-knockdown and scramble control A549/cis and Pc9/cis cells were treated with 2 μg/mL cisplatin or vehicle control for 24 hours and then subjected to IF assays. Scale bar: 50 μm. **(d-e)** Transwell assay showing the effect of DCBLD2 on cell migration after cisplatin treatment. Stable DCBLD2-knockdown cells and scramble control cells were pretreated with 2 μg/mL cisplatin or vehicle control for 24 hours and then subjected to Transwell assays. Images of the cells on the underside of the membrane of wells are shown in (d) after incubation for 6 hours in the presence of a chemoattractant. Scale bar: 200 μm. Quantification of migrated cells is depicted in (e), ** $P < 0.01$, *** $P < 0.001$, t-test. **(f-g)** Stable DCBLD2-knockdown cells and scramble control cells were cultured in 2 μg/mL cisplatin or vehicle control for 24 hours and then subjected to wound healing assays to confirm the effect of DCBLD2 on cell migration under cisplatin treatment. Images of the cell distribution at 0, 24, or 48 hours after scratching are shown in (f). Scale bar: 200 μm. Quantification of the migrated distance is depicted in (g), * $P < 0.05$, *** $P < 0.001$, t-test. **(h)** Stable DCBLD2-knockdown cells and scramble control cells were treated with cisplatin for 24 hours to analyze their sensitivity to cisplatin.

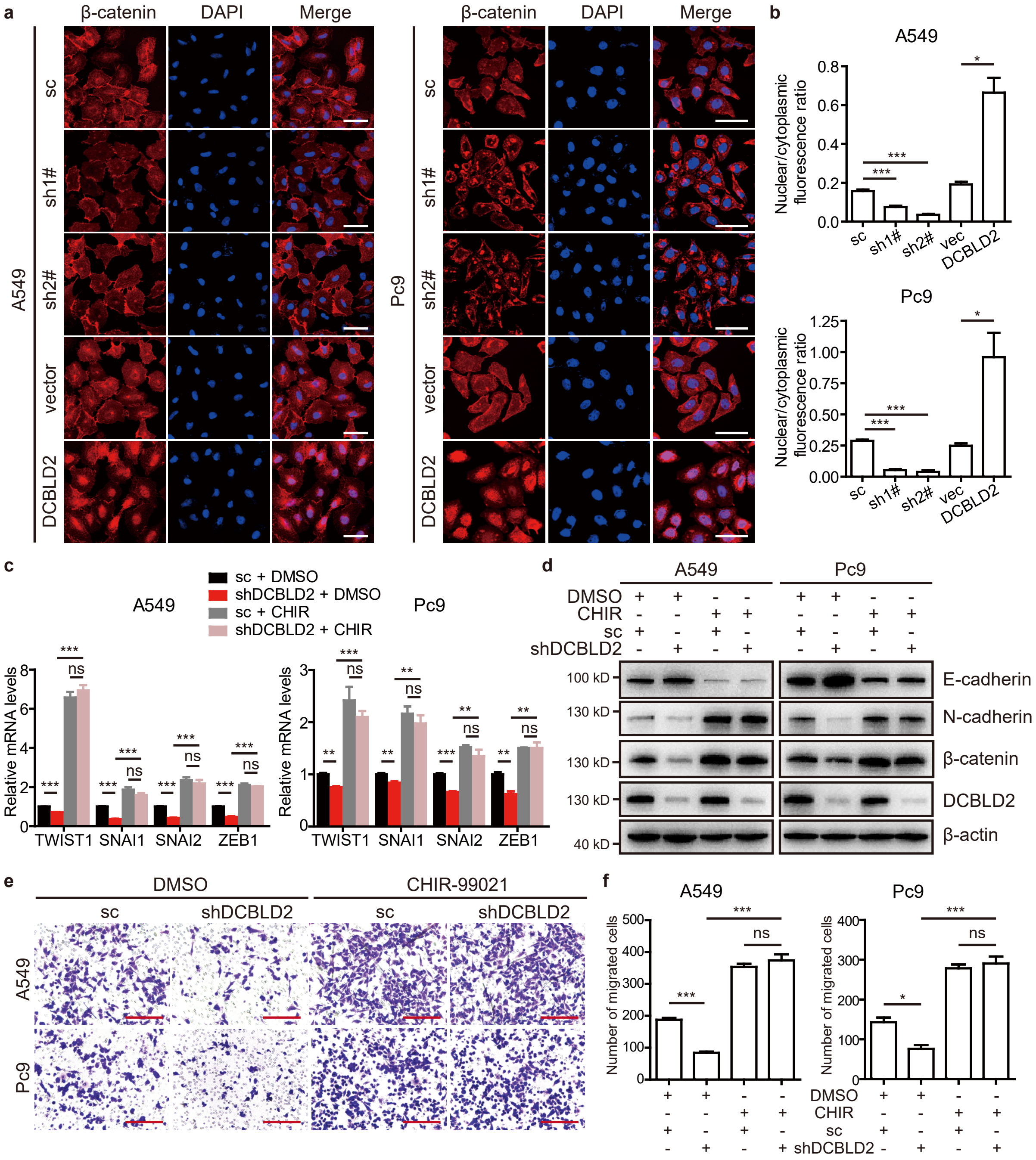

Figure S5. DCBLD2 modulates EMT via the Wnt/β-catenin signaling pathway in LUAD. **(a-b)** IF assay of the nuclear localization of β-catenin in A549 and Pc9 cells. DAPI was used for nuclear staining. Scale bar: 50 μm. The nuclear/cytoplasmic fluorescence ratio is shown in (b), * $P < 0.05$, *** $P < 0.001$, t-test. **(c)** RT-PCR assays of the expression of EMT-related TFs in cells transfected with scramble control or shDCBLD2 after treatment with 2 μM CHIR-99021 or DMSO for 24 hours. ** $P < 0.01$, *** $P < 0.001$, t-test. **(d)** Western blot assay of the expression of E-cadherin and N-cadherin in cells transfected with scramble control or shDCBLD2 after treatment with 2 μM CHIR-99021 or DMSO for 24 hours. **(e-f)** Transwell assay showing the effect of β-catenin on DCBLD2-induced cell migration. Scramble control and shDCBLD2-transfected cells were pretreated with 2 μM CHIR-99021 or DMSO for 24 hours and then subjected to Transwell assays. Images of the cells on the underside of the membrane of wells are shown in (e) after culture for 6 hours in the presence of a chemoattractant. Scale bar: 200 μm. Quantification of migrated cells is depicted in (f), * $P < 0.05$, *** $P < 0.001$, t-test.

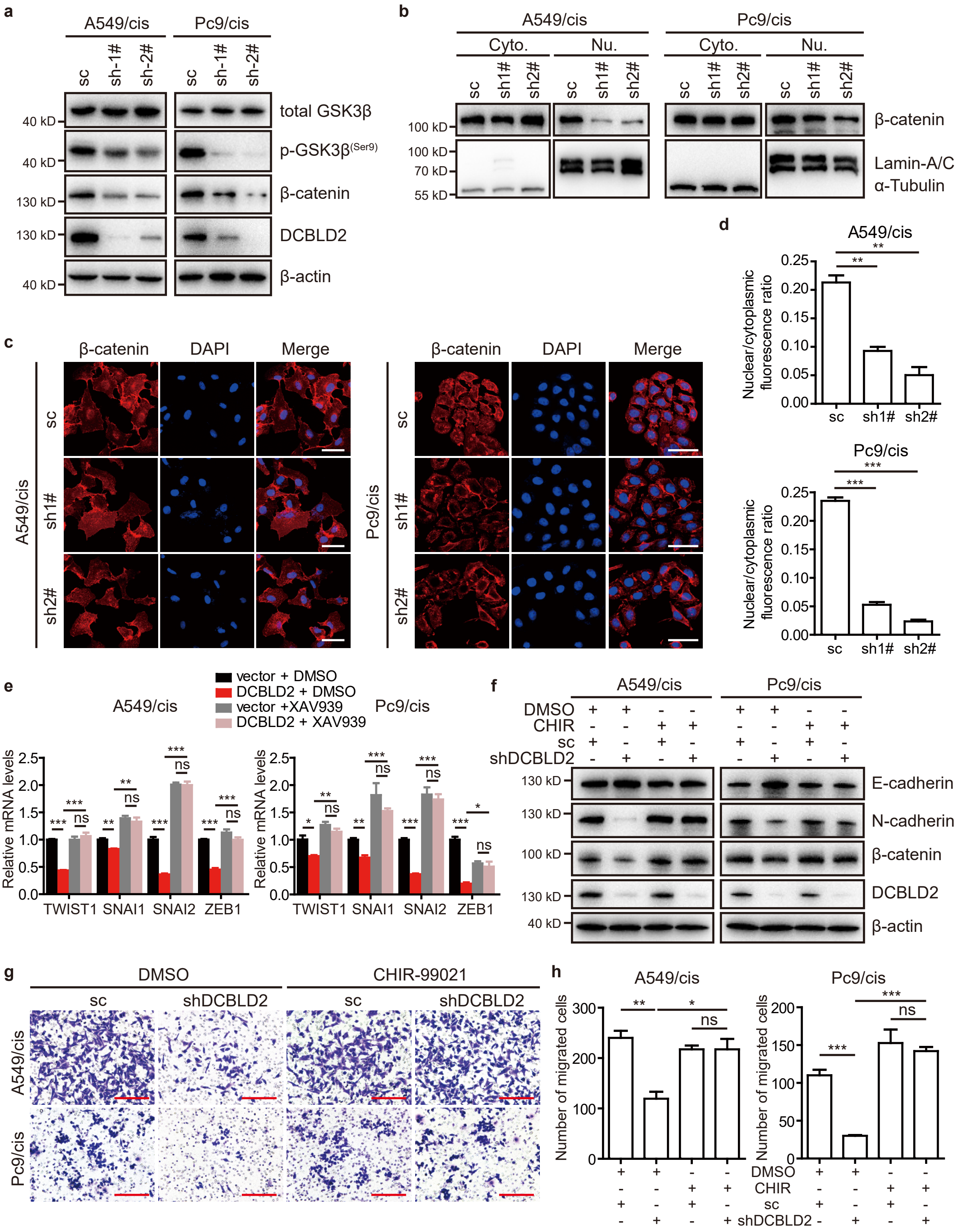

Figure S6. DCBLD2 modulates EMT via the Wnt/β-catenin signaling pathway in cisplatin-resistant LUAD. **(a)** Western blot assay of β-catenin and p-GSK3β levels in A549/cis and Pc9/cis cells. **(b)** Western blot assay of β-catenin expression after nuclear-cytoplasmic fractionation in A549/cis and Pc9/cis cells. α-Tubulin and Lamin A/C were used as cytoplasmic and nuclear loading controls, respectively. **(c-d)** IF assay of the nuclear localization of β-catenin in A549/cis and Pc9/cis cells. DAPI was used for nuclear staining. Scale bar: 50 μm. The nuclear/cytoplasmic fluorescence ratio is shown in (d), ** $P < 0.01$, *** $P < 0.001$, t-test. **(e)** RT-PCR assays of the expression of EMT-related TFs in cells transfected with scramble control or shDCBLD2 after treatment with 2 μM CHIR-99021 or DMSO for 24 hours. ** $P < 0.01$, *** $P < 0.001$, t-test. **(f)** Western blot assay of the expression of E-cadherin and N-cadherin in cells transfected with scramble control or shDCBLD2 after treatment with 2 μM CHIR-99021 or DMSO for 24 hours. **(g-h)** Transwell assay showing the effect of β-catenin on DCBLD2-induced cell migration. Scramble control and shDCBLD2-transfected cells were pretreated with 2 μM CHIR-99021 or DMSO for 24 hours and then subjected to Transwell assays. Images of the cells on the underside of the membrane of wells are shown in (g) after culture for 9 hours in the presence of a chemoattractant. Scale bar: 200 μm. Quantification of migrated cells is depicted in (h), * $P < 0.05$, *** $P < 0.001$, t-test.

Figure S7. Uncropped western blot images

Uncropped western blot images for Figure 1e

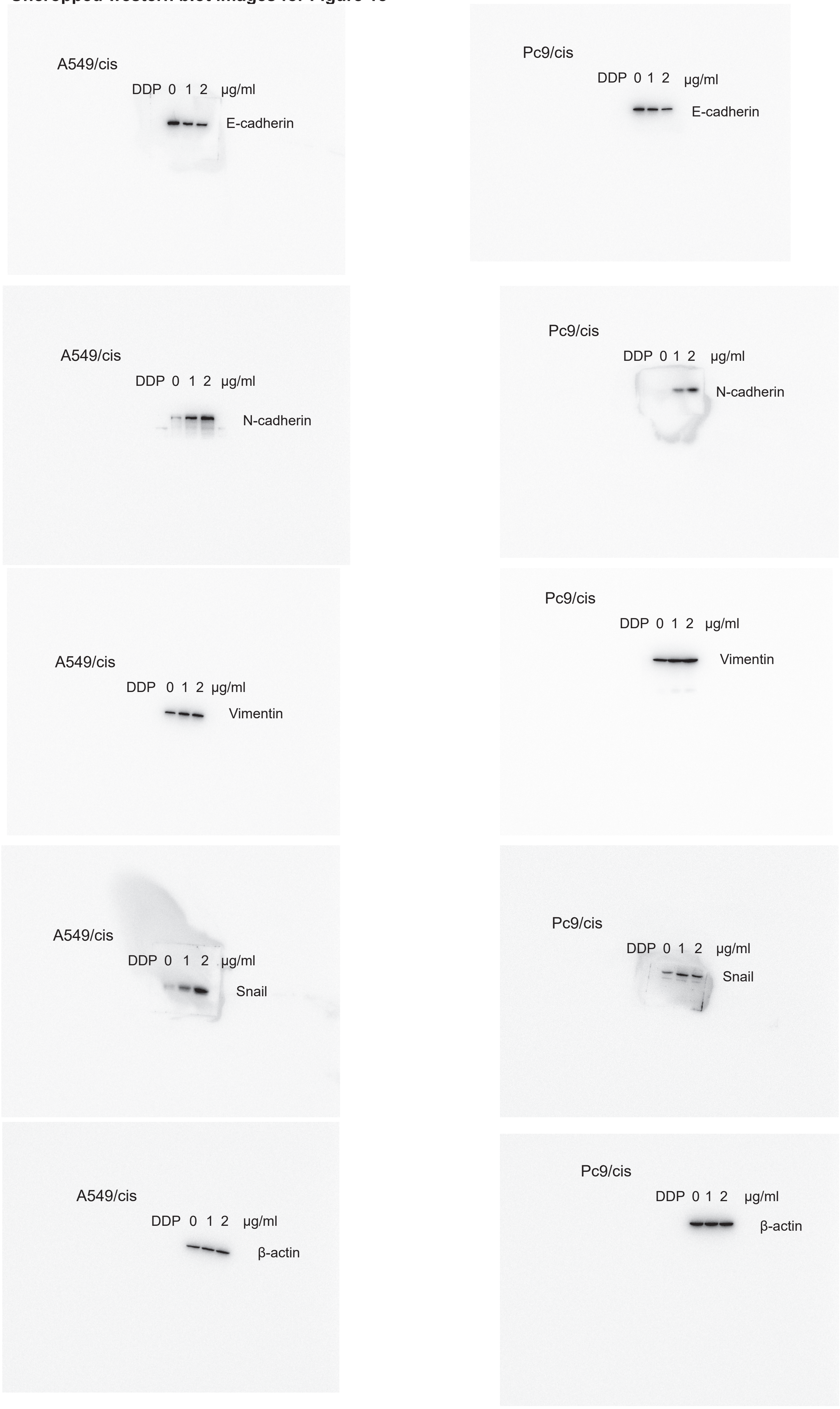

Uncropped western blot images for Figure 3g

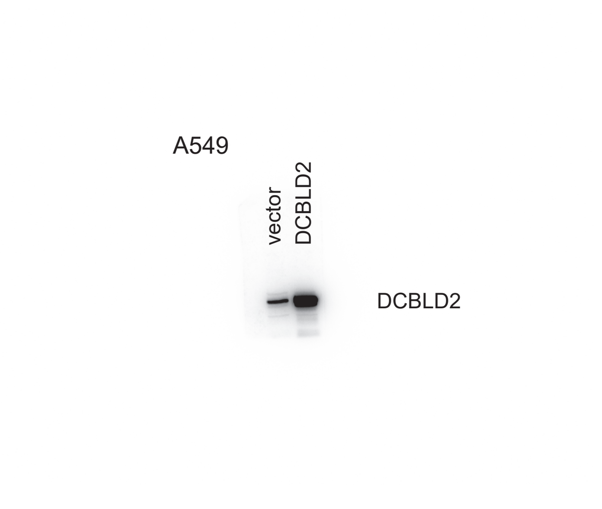

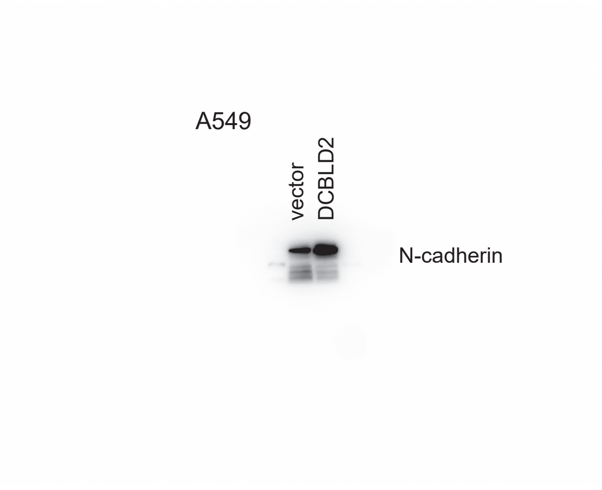

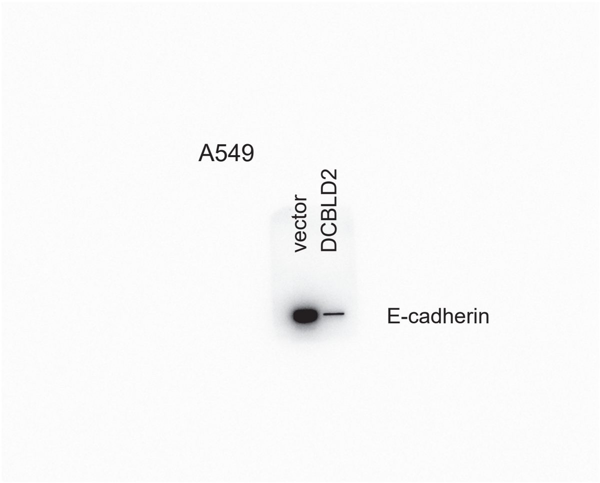

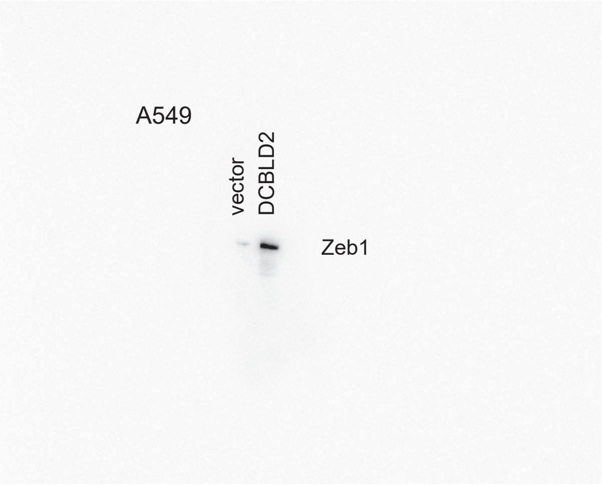

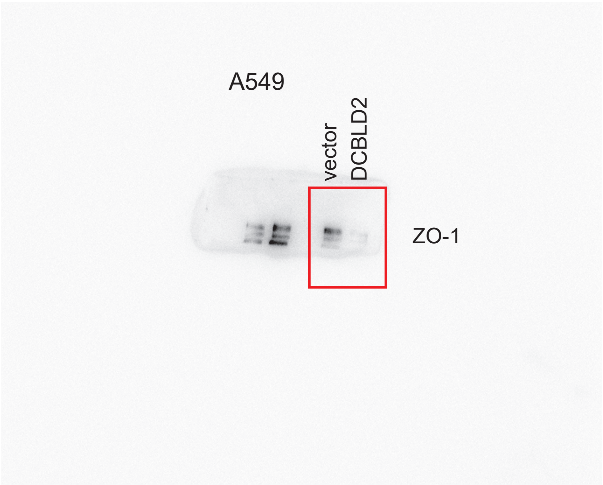

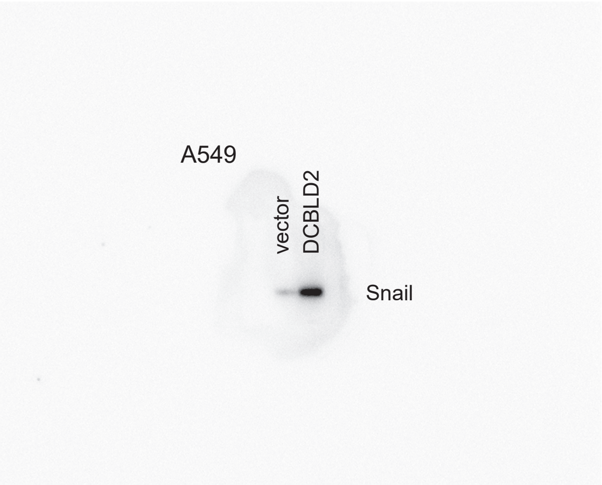

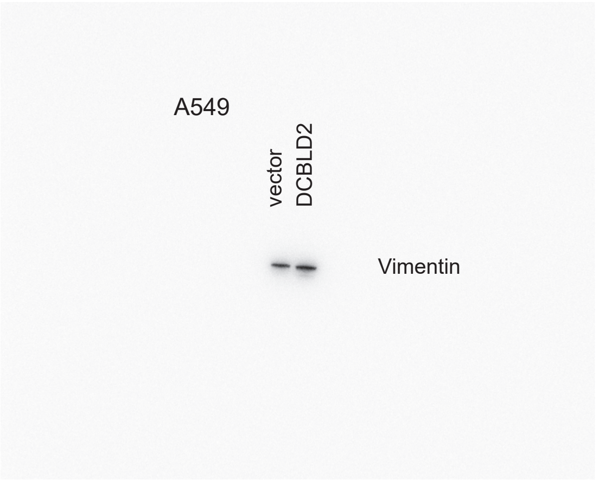

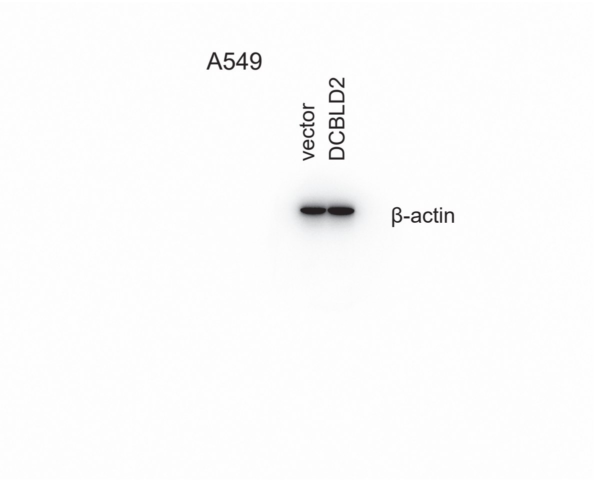

Uncropped western blot images for Figure 3g

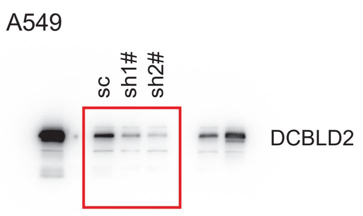

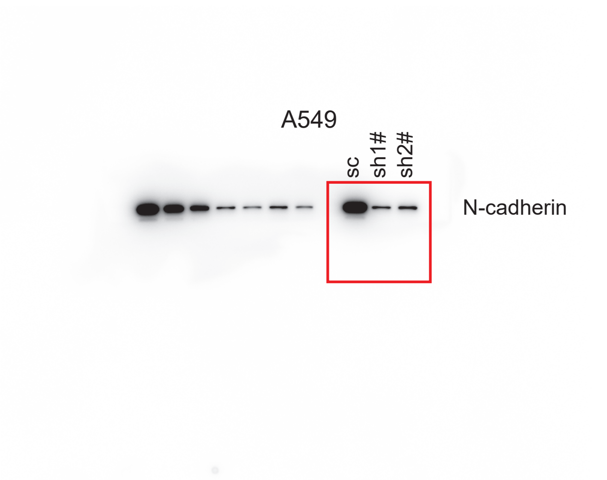

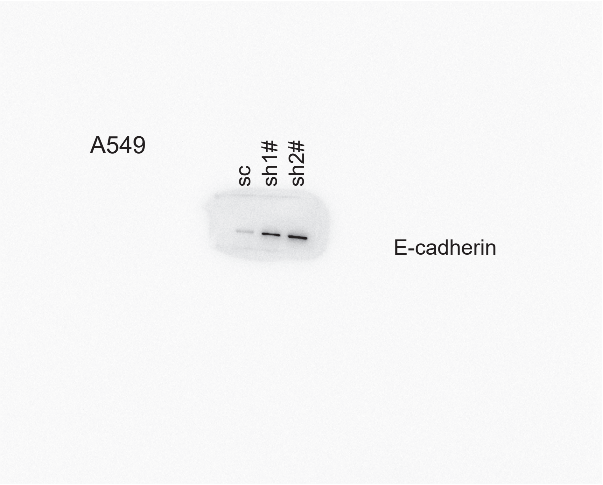

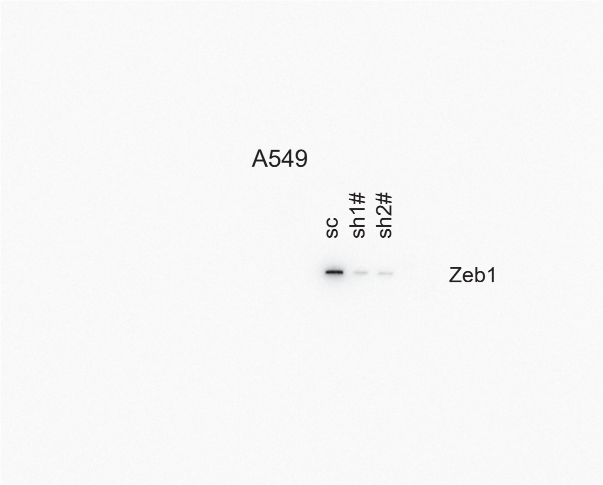

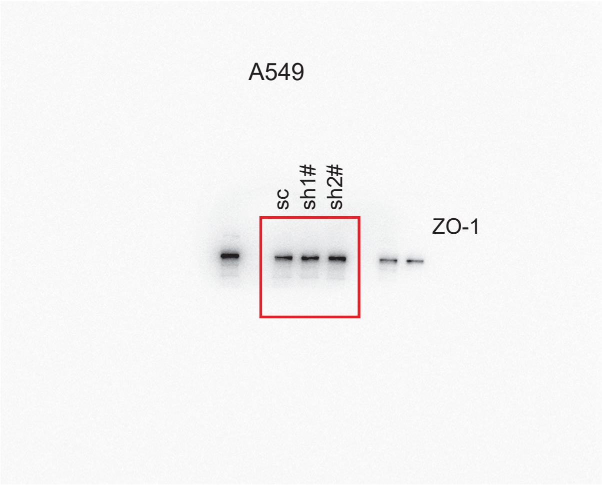

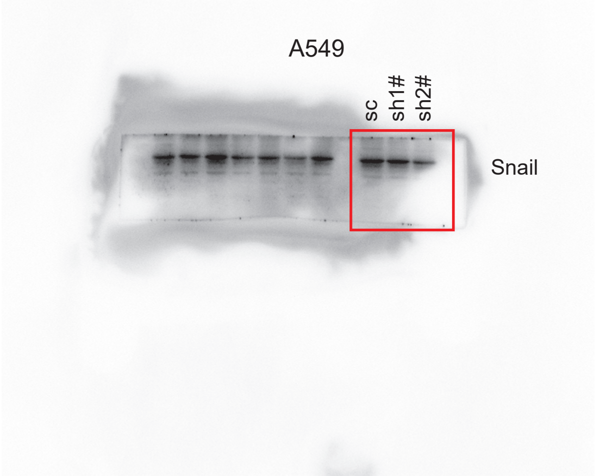

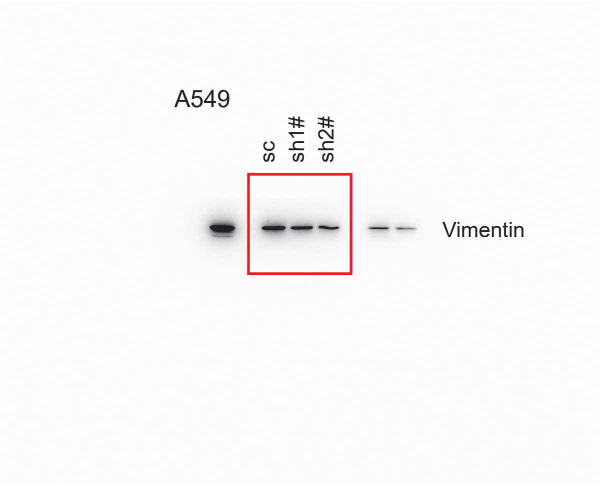

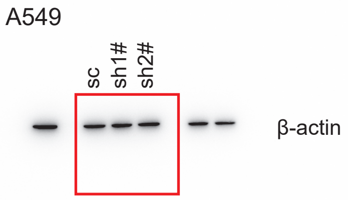

Uncropped western blot images for Figure 3g

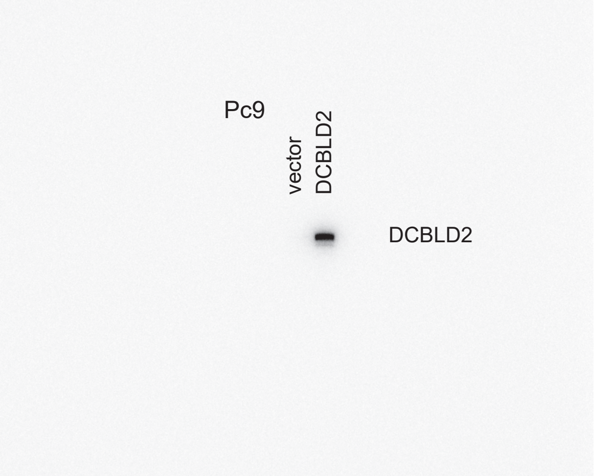

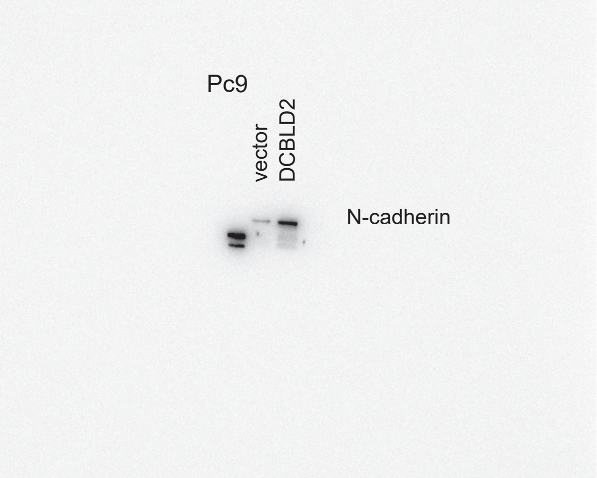

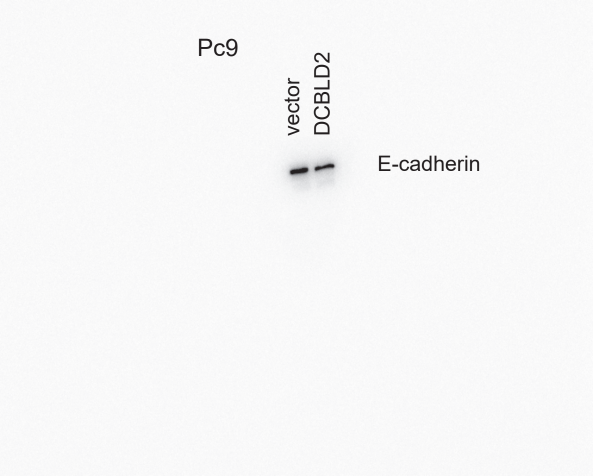

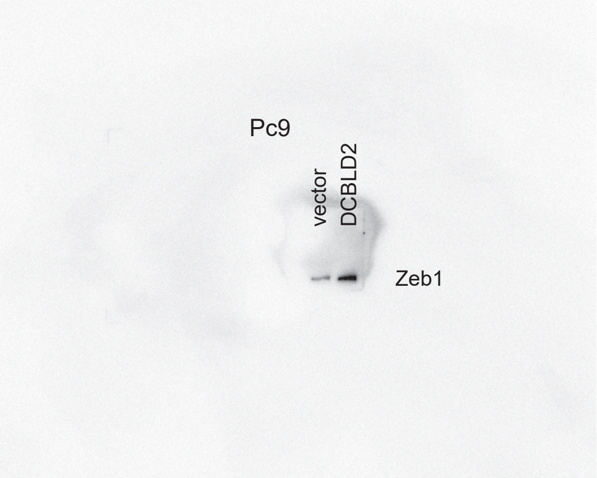

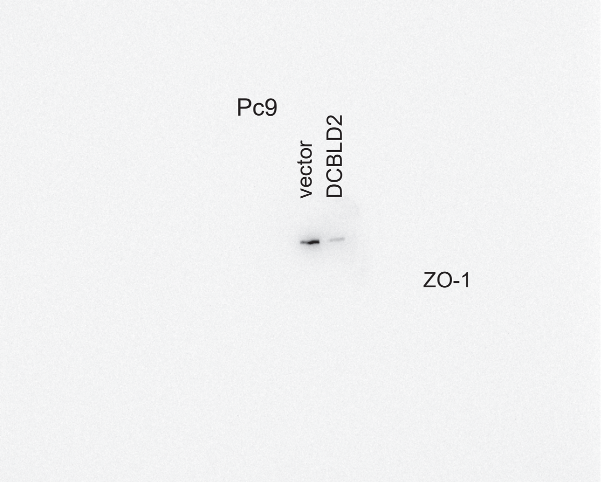

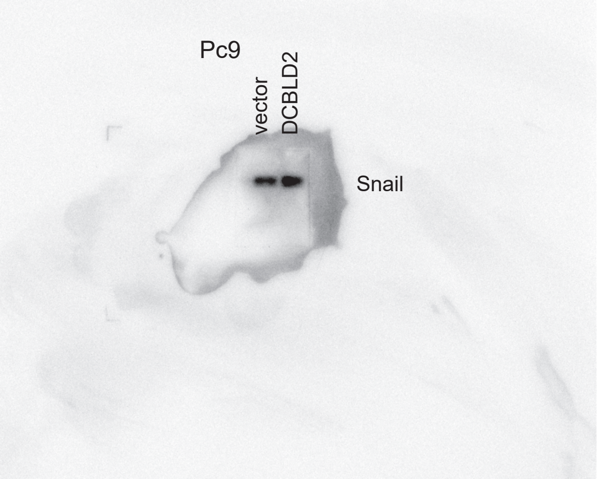

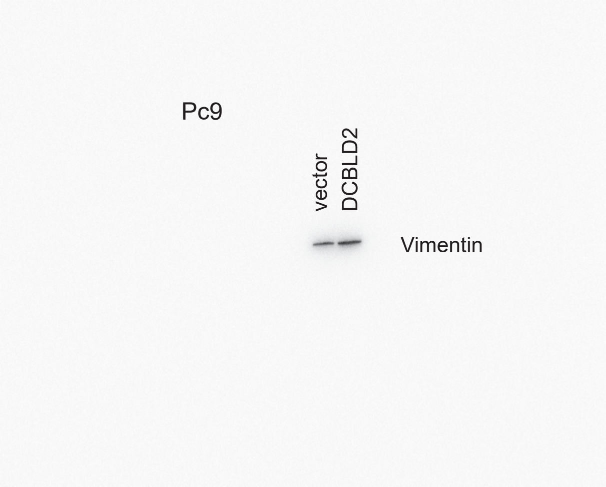

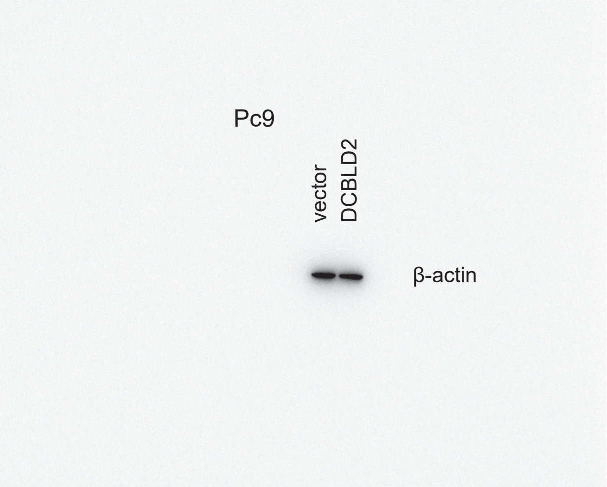

Uncropped western blot images for Figure 3g

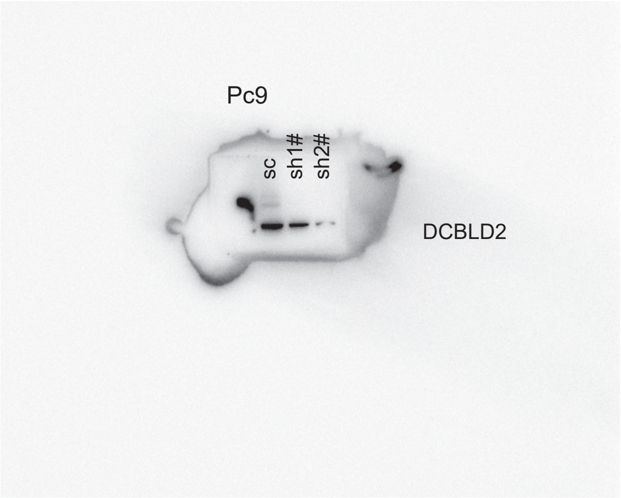

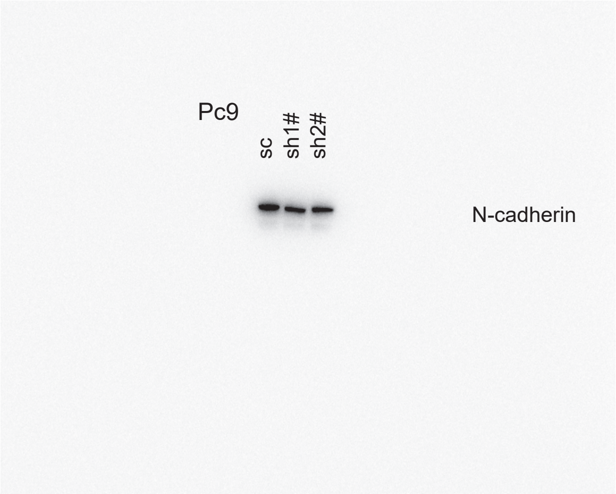

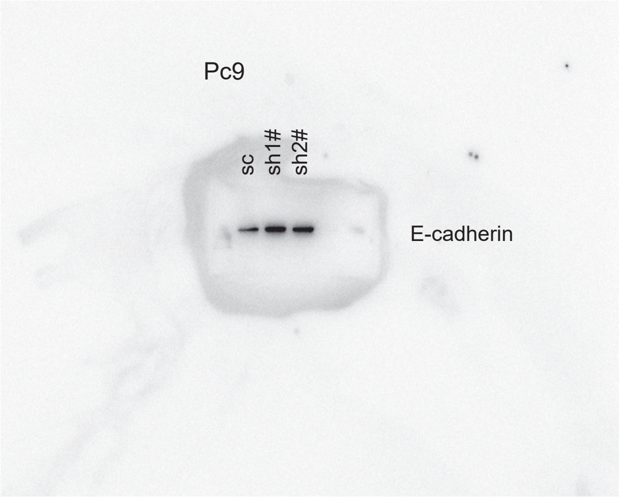

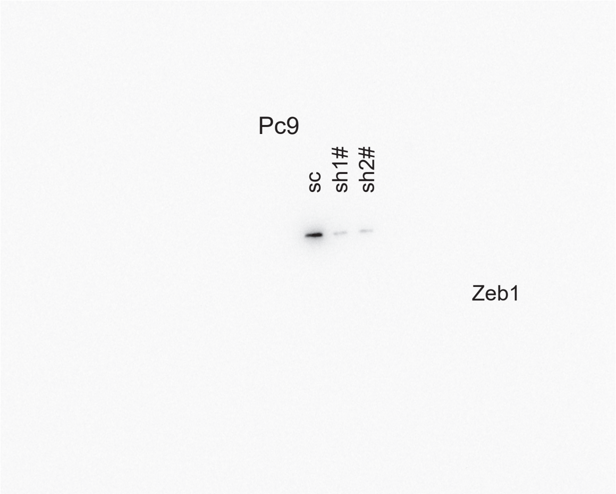

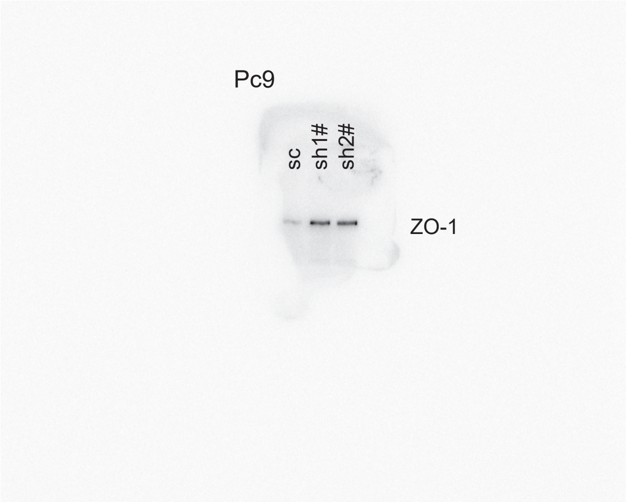

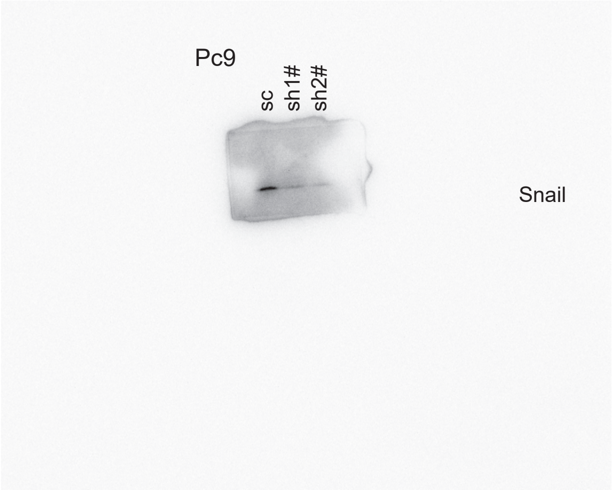

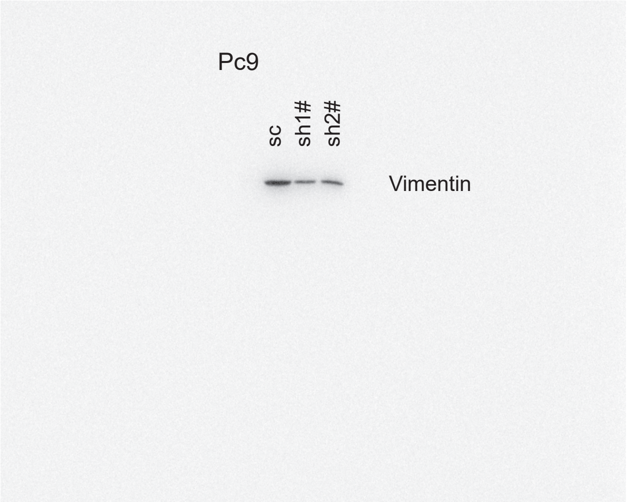

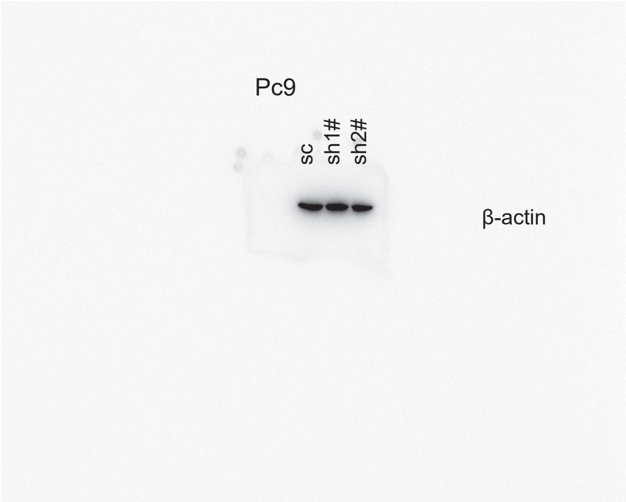

Uncropped western blot images for Figure 4d

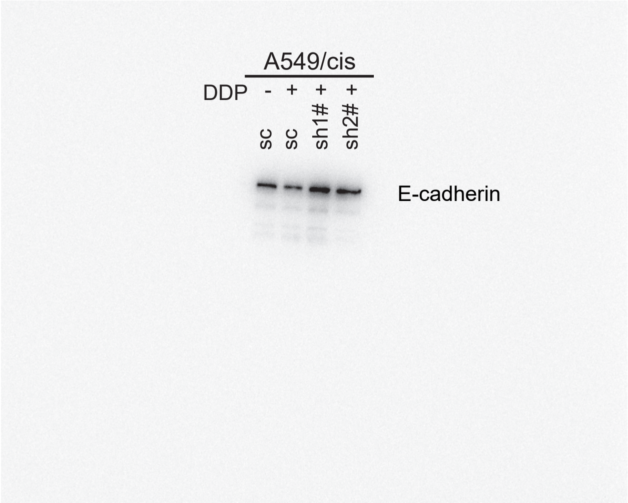

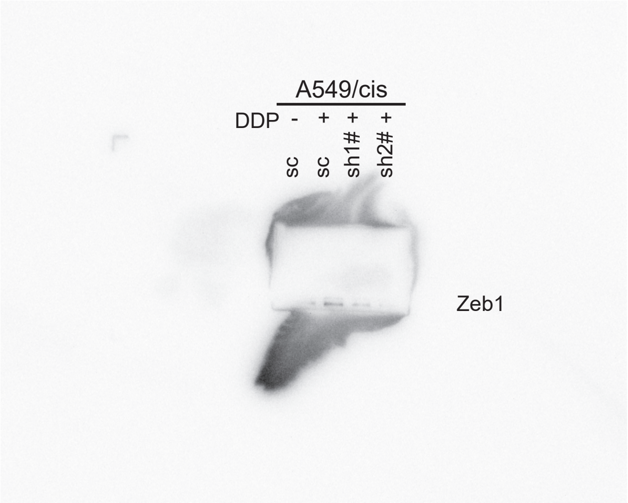

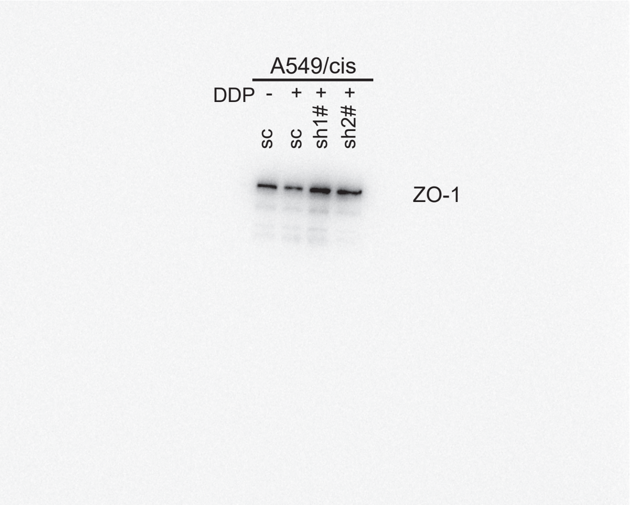

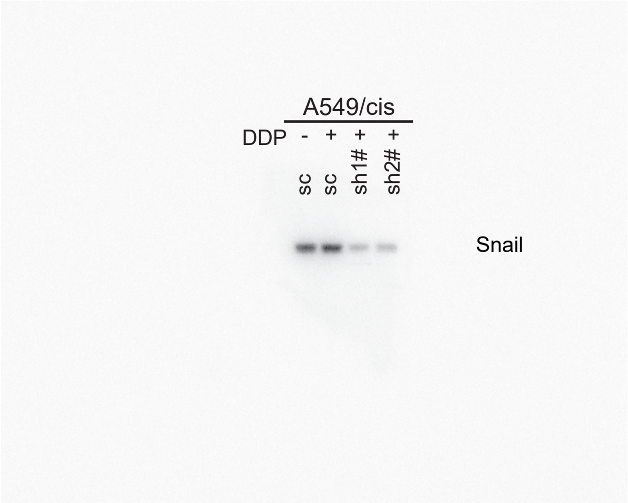

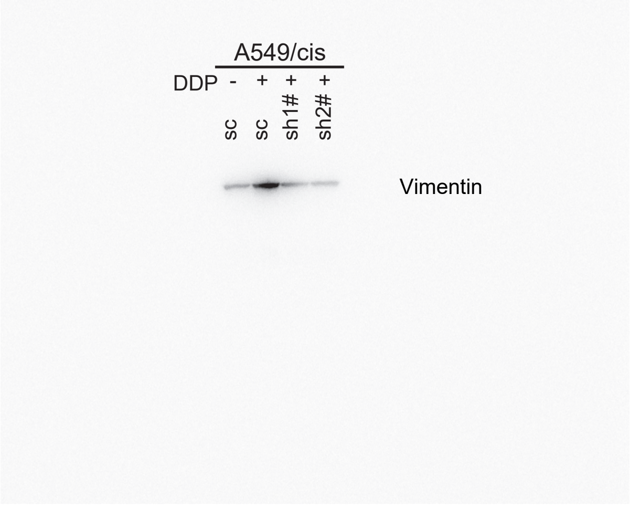

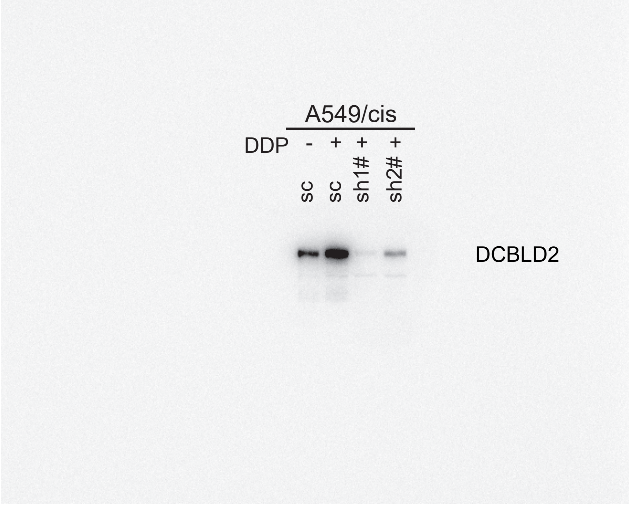

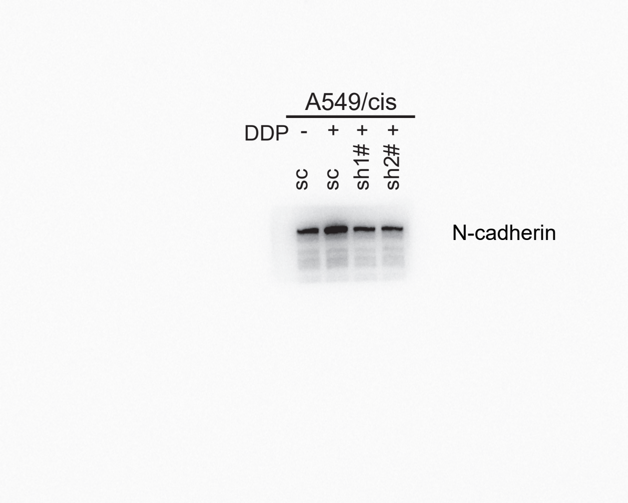

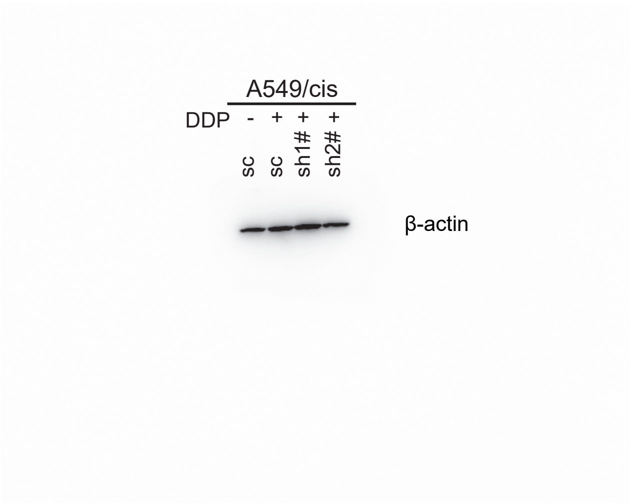

Uncropped western blot images for Figure 4d

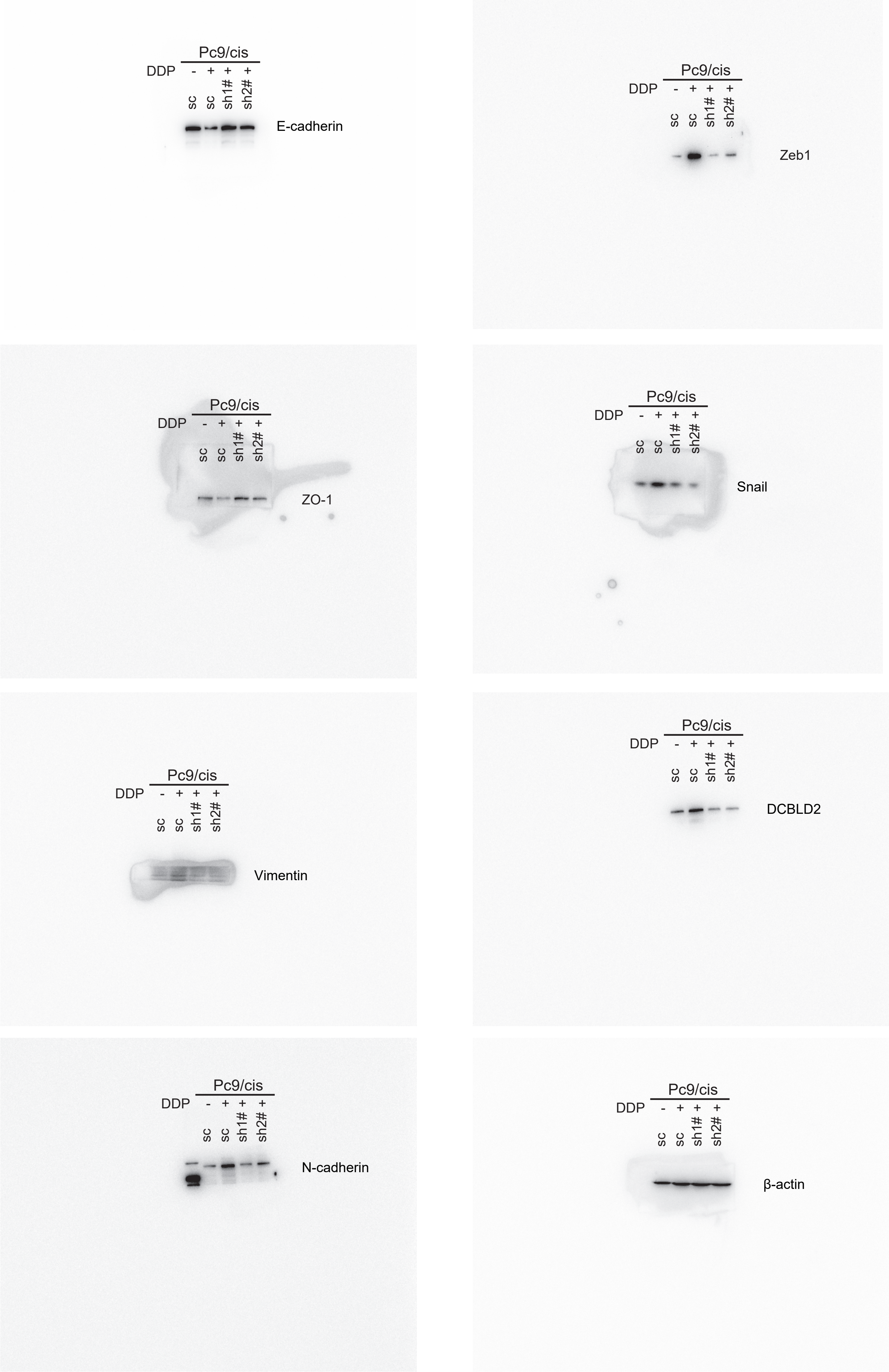

Uncropped western blot images for Figure 5e

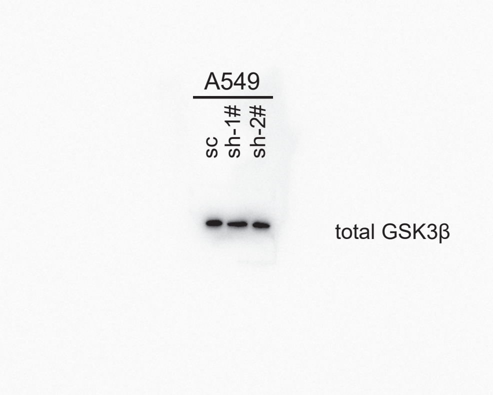

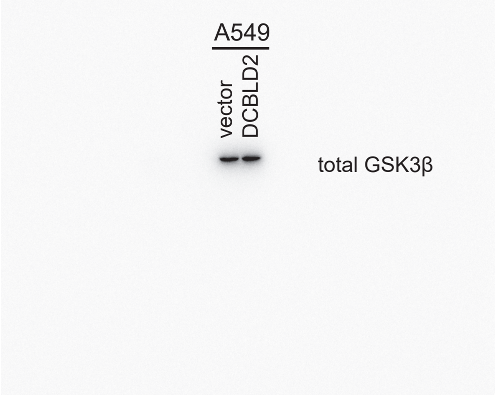

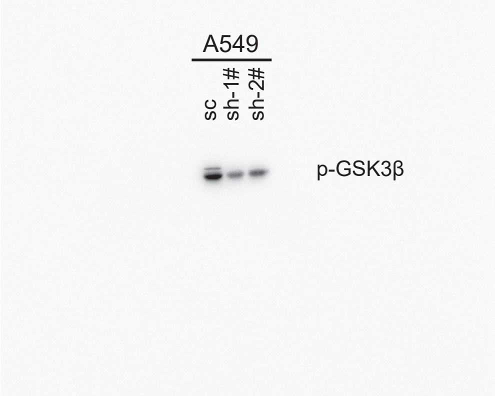

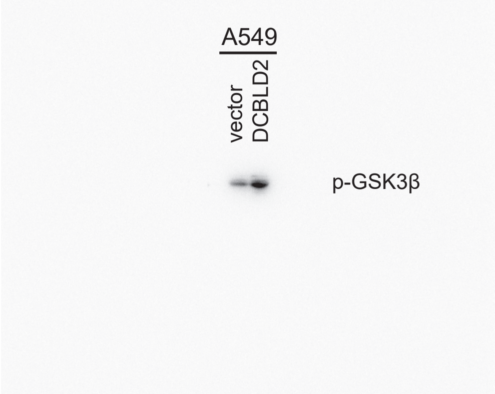

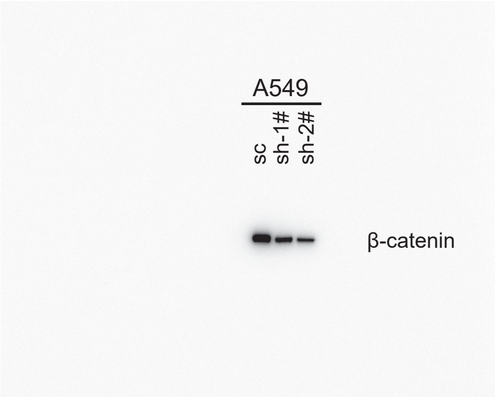

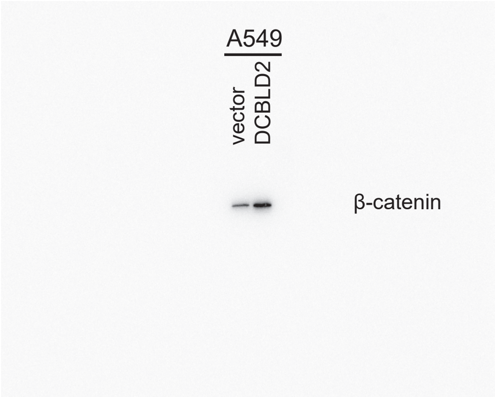

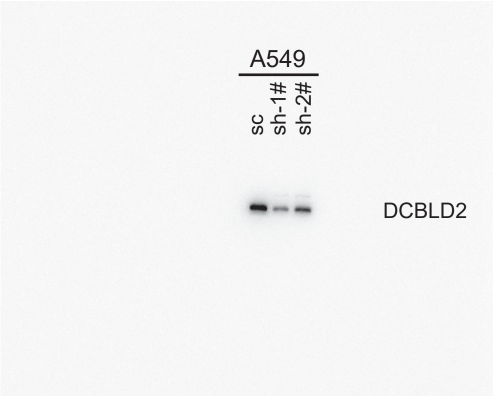

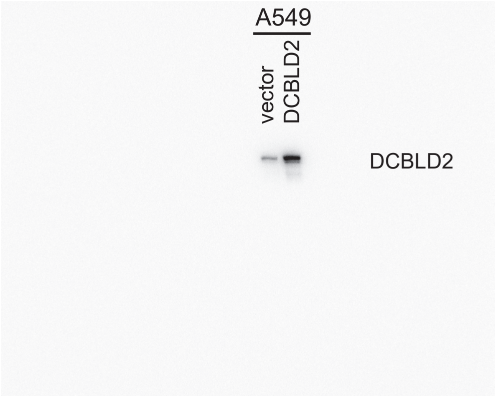

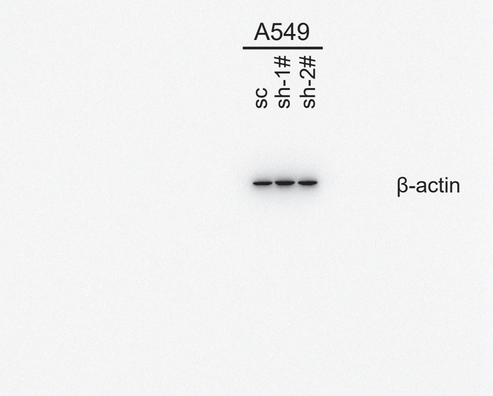

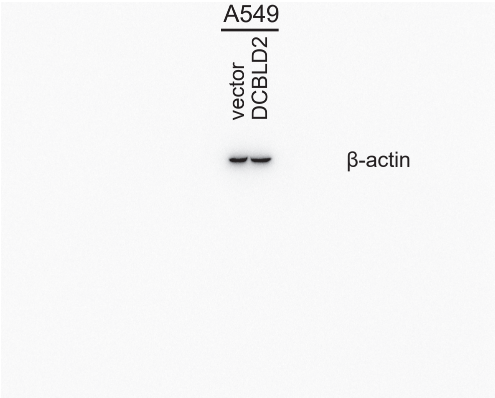

Uncropped Western blot images for Figure 5e

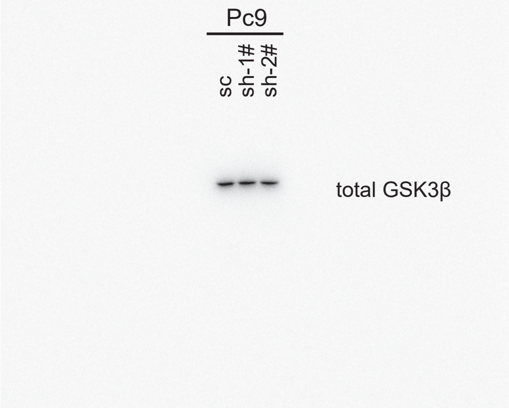

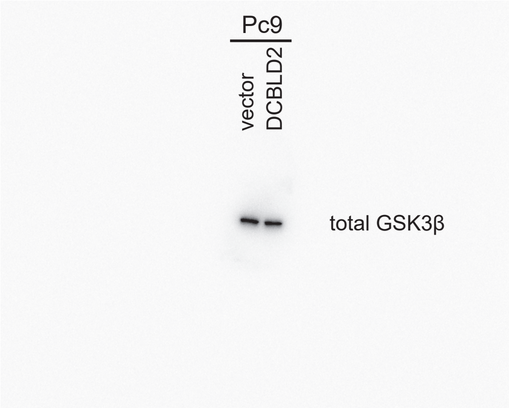

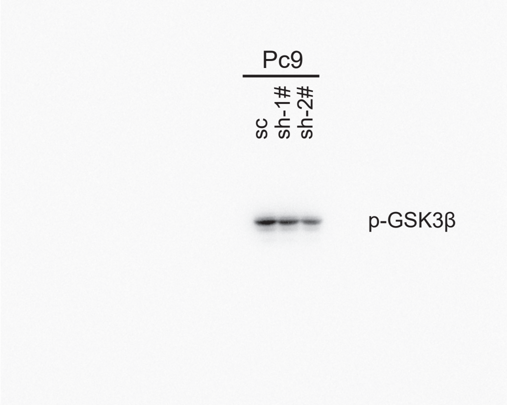

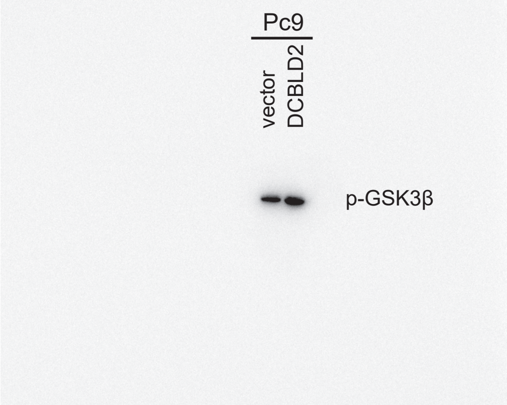

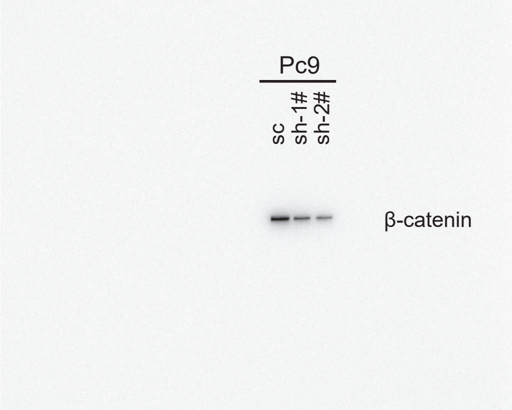

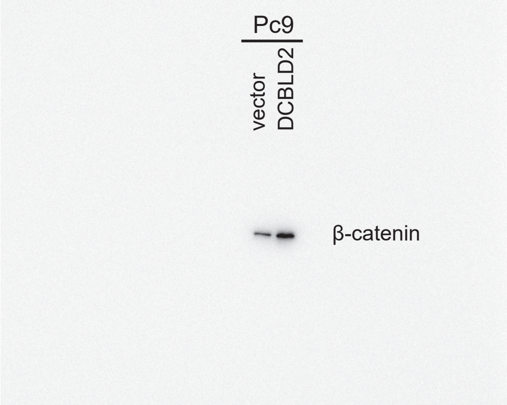

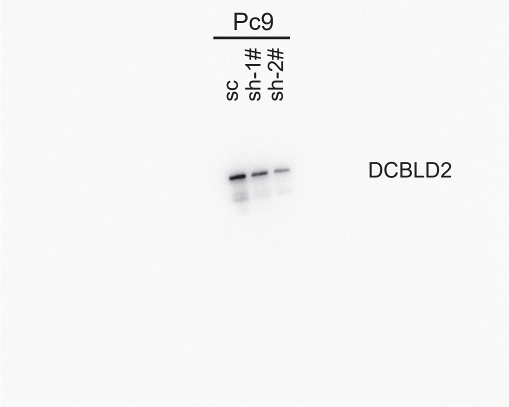

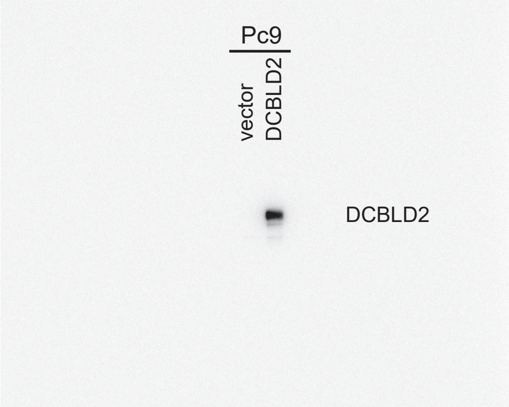

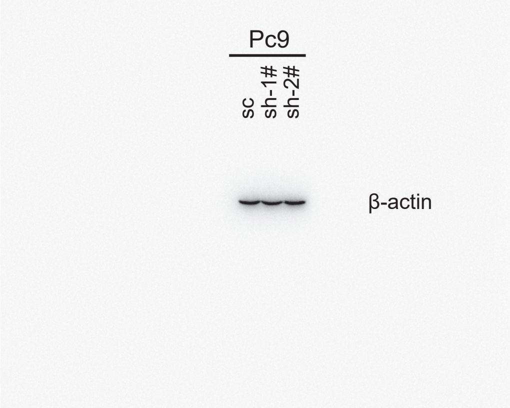

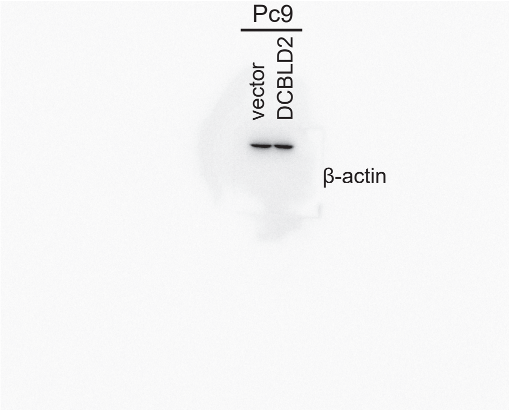

Uncropped Western blot images for Figure 5f

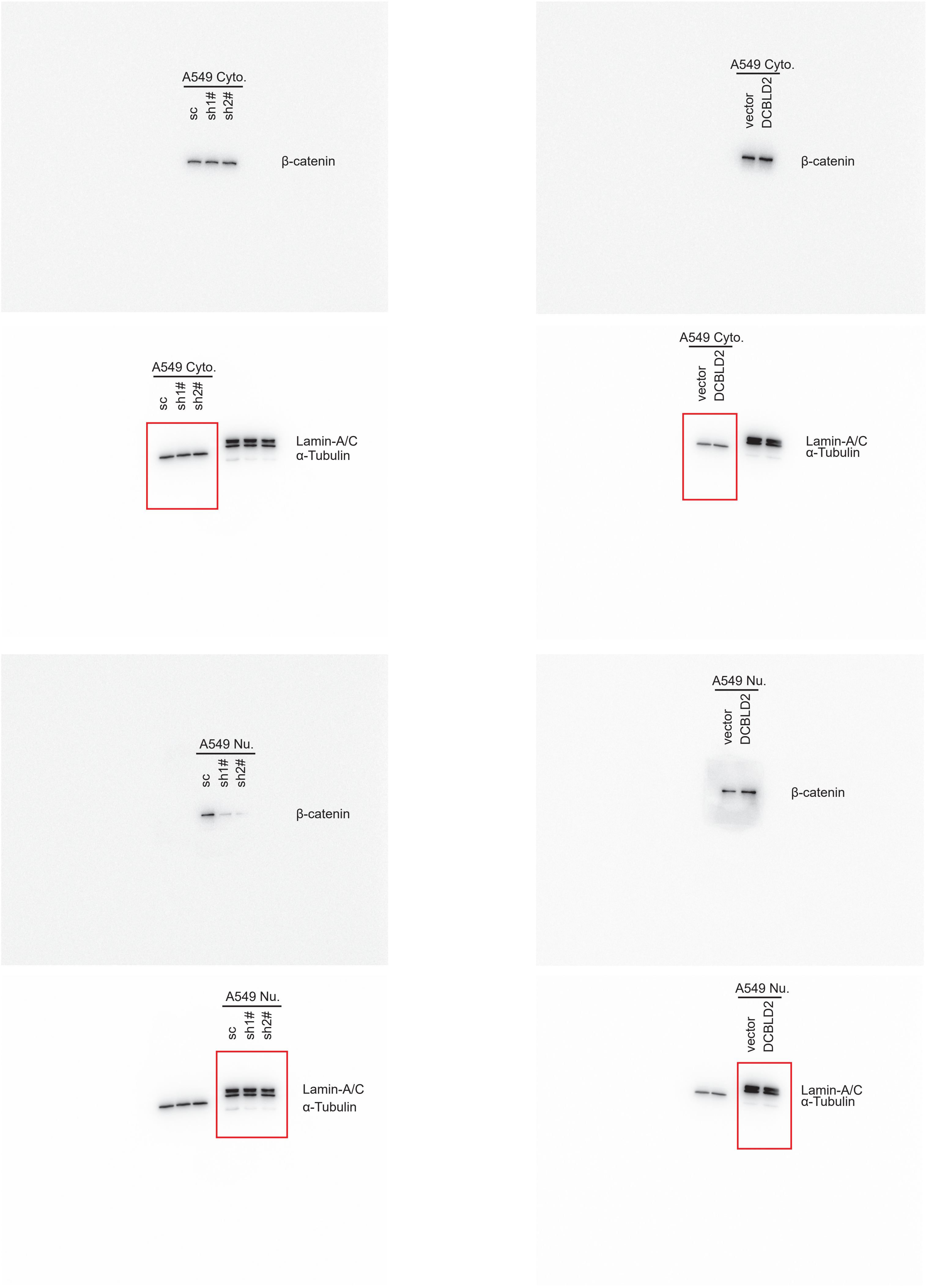

Uncropped Western blot images for Figure 5f

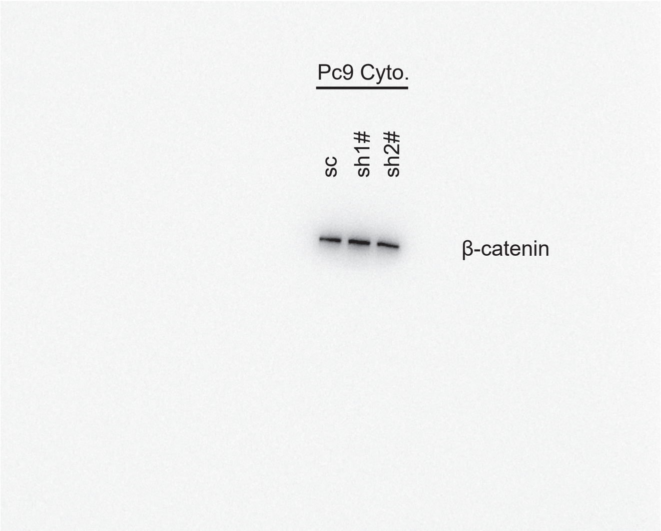

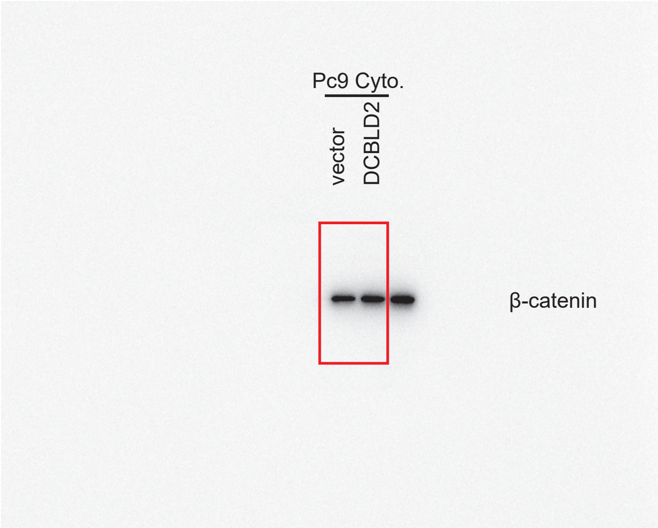

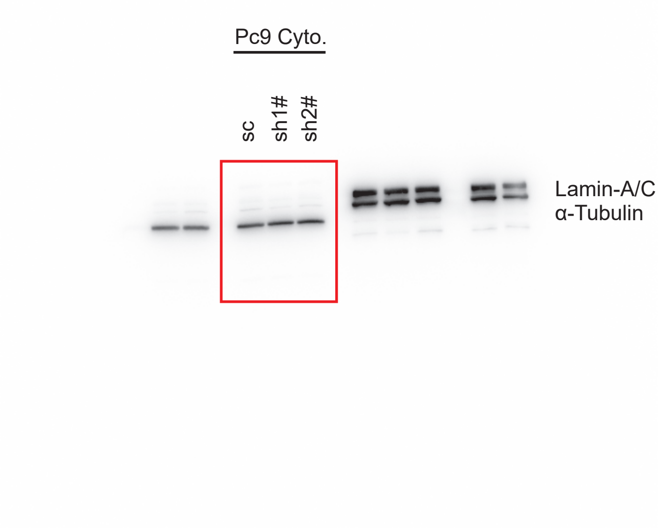

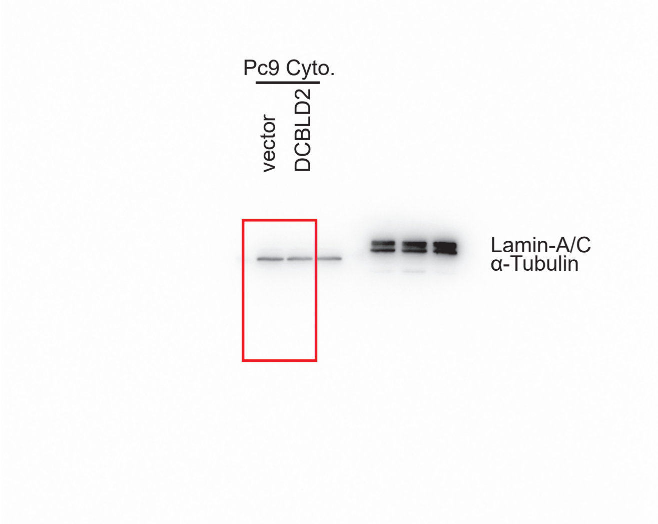

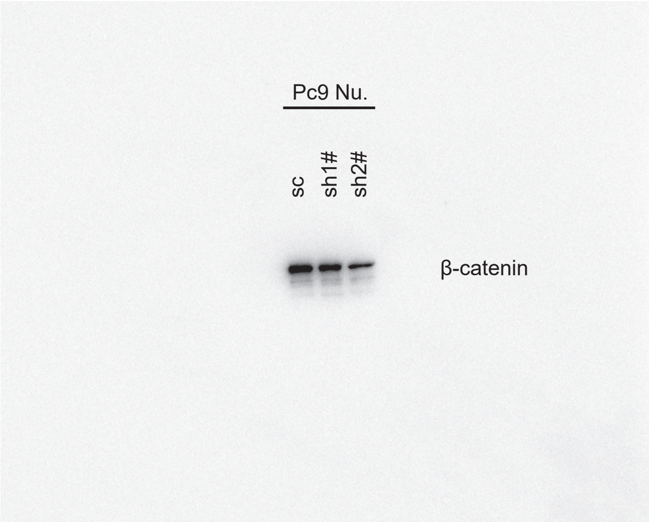

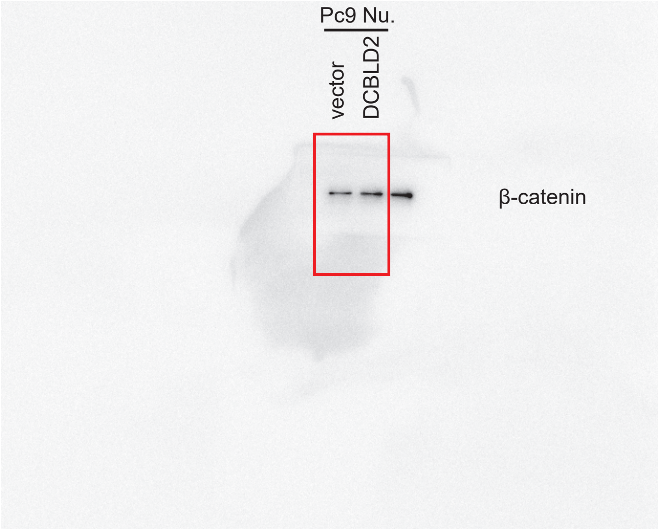

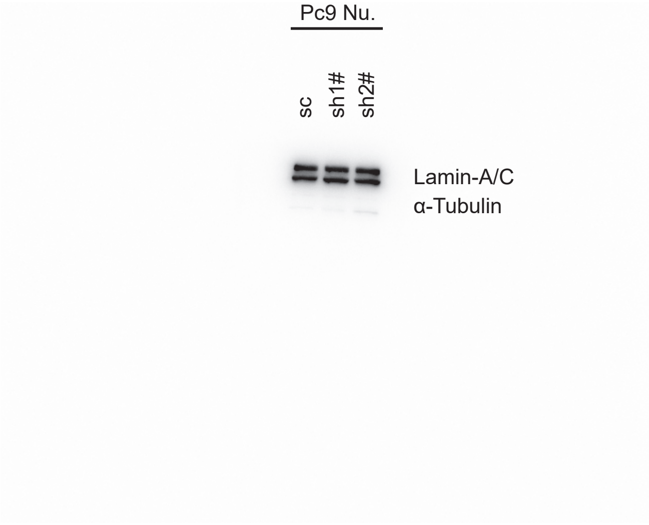

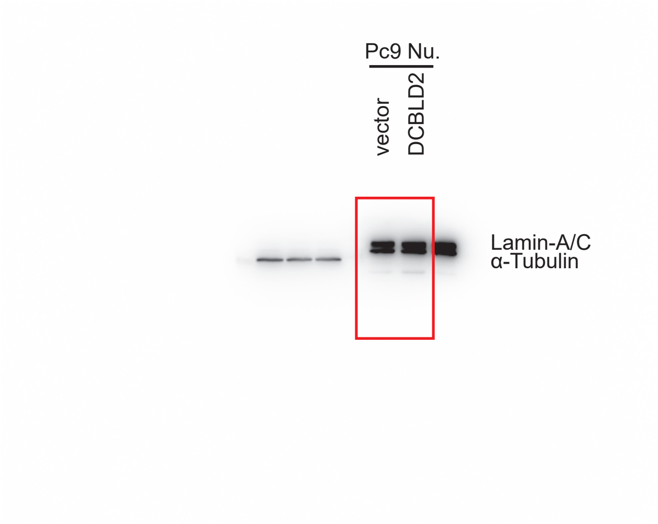

Uncropped Western blot images for Figure 5h

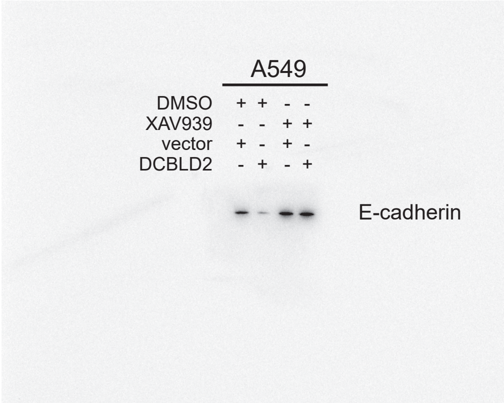

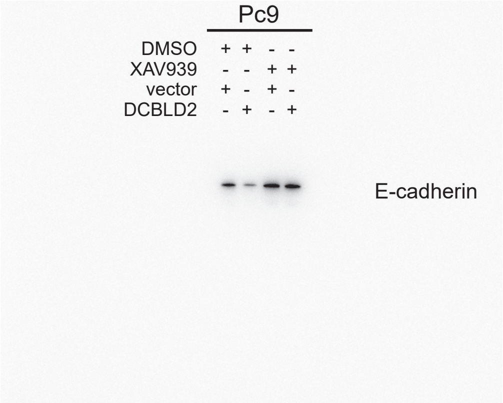

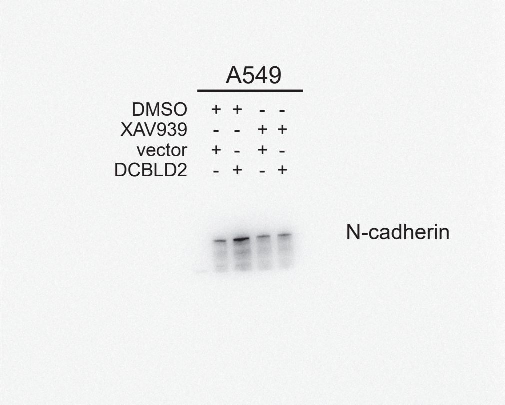

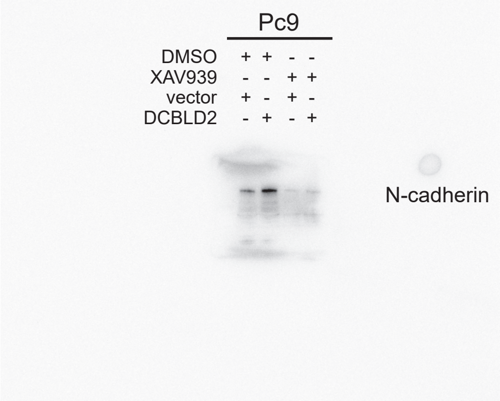

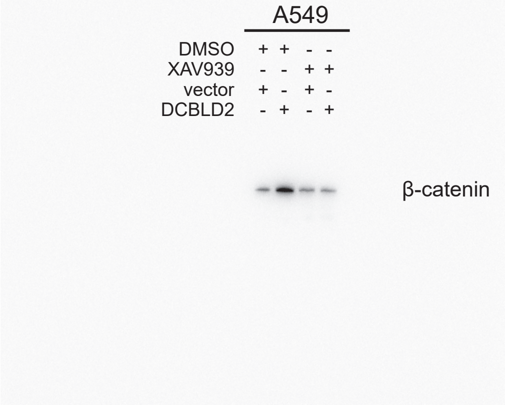

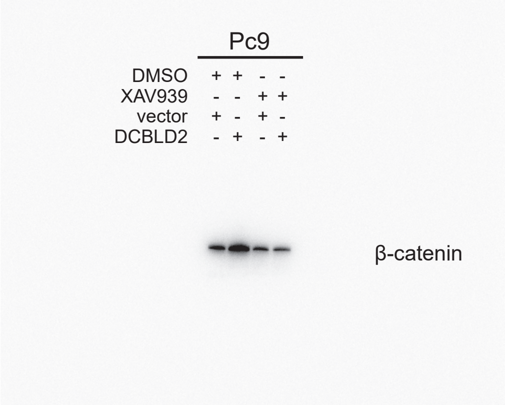

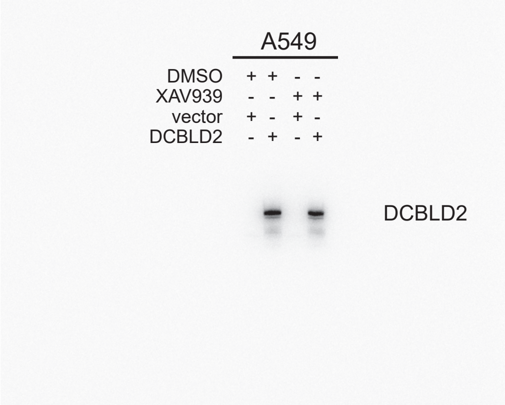

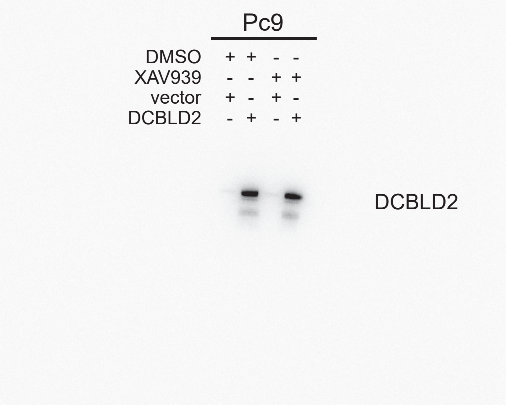

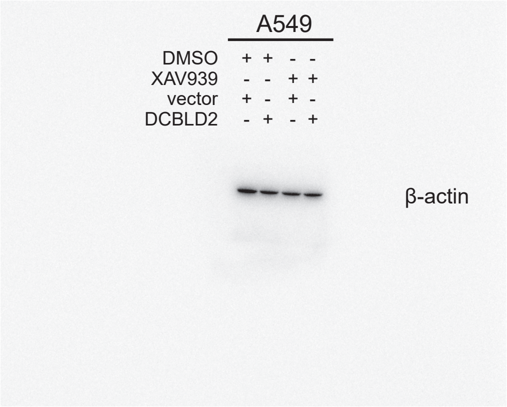

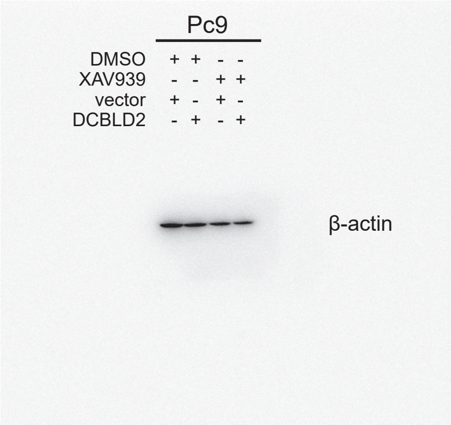

Uncropped Western blot images for Figure 6b

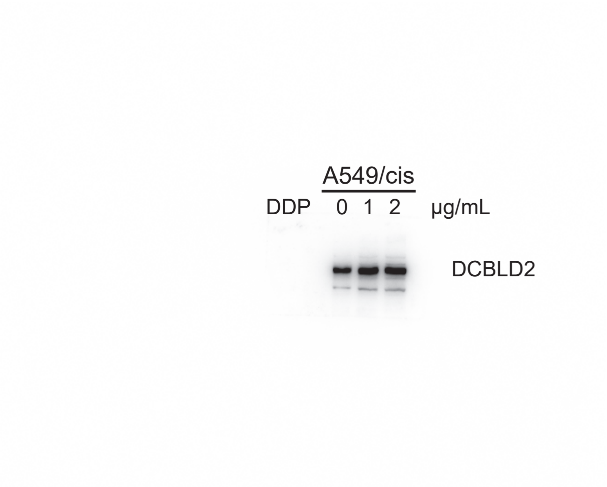

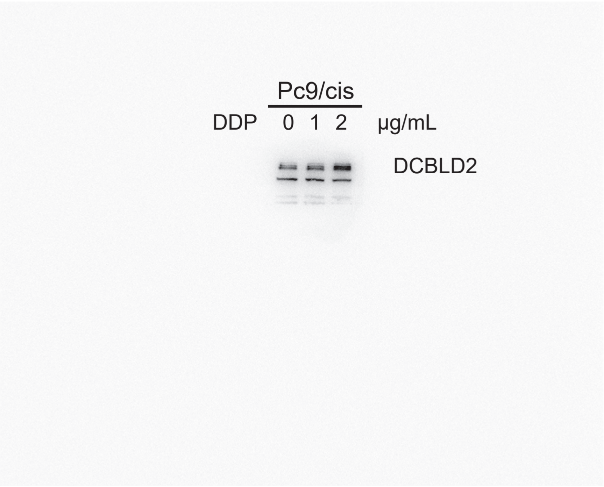

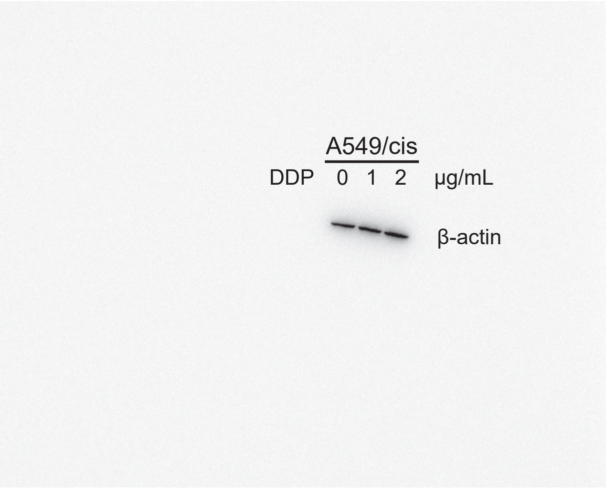

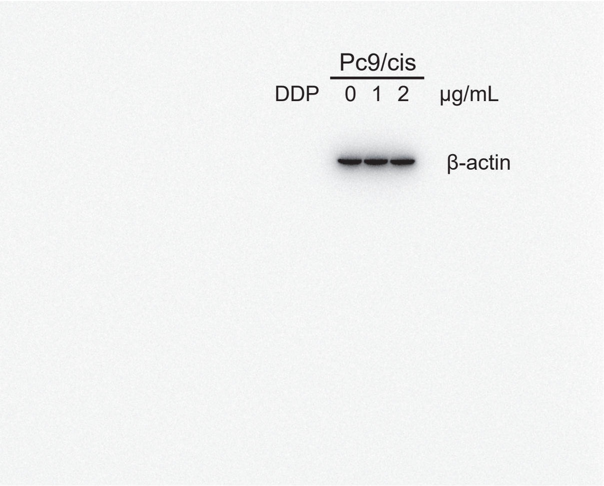

Uncropped Western blot images for Figure 6e

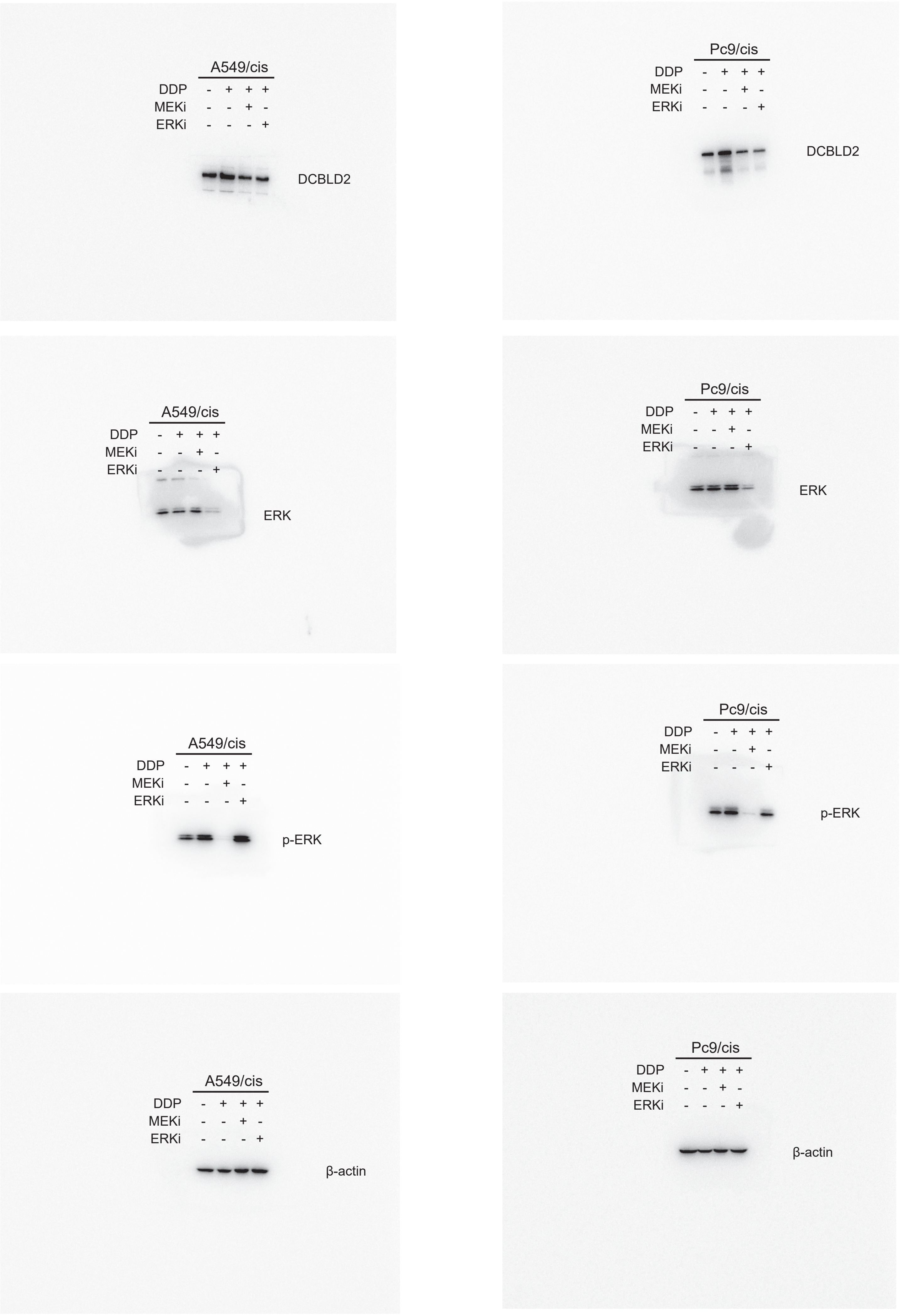

Uncropped Western blot images for Figure 6h

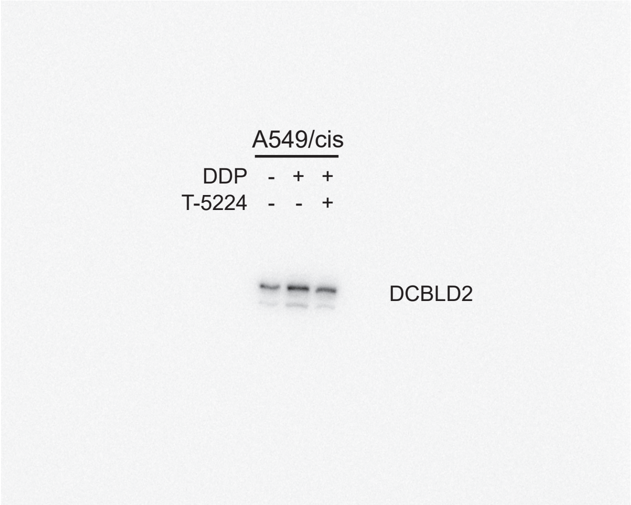

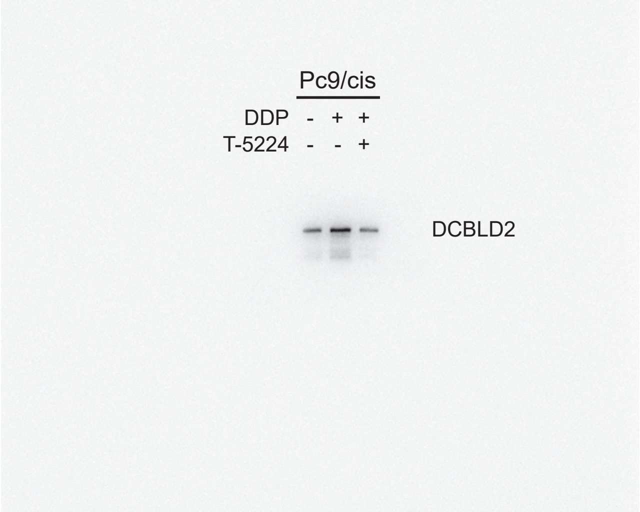

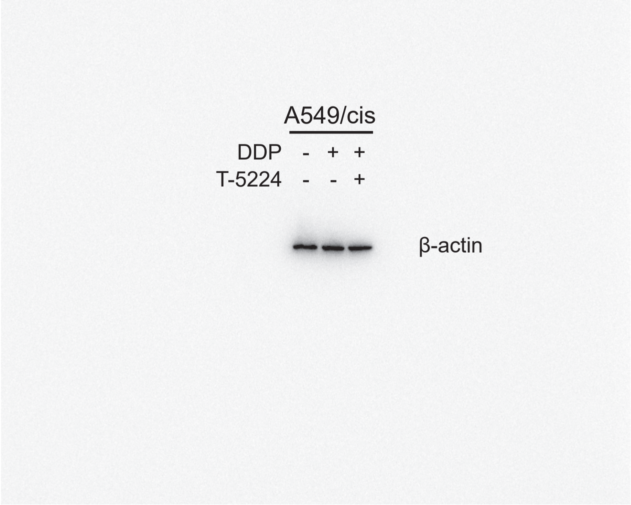

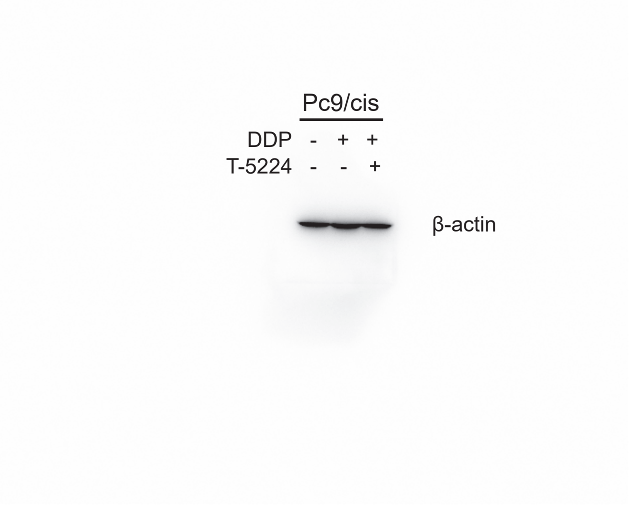

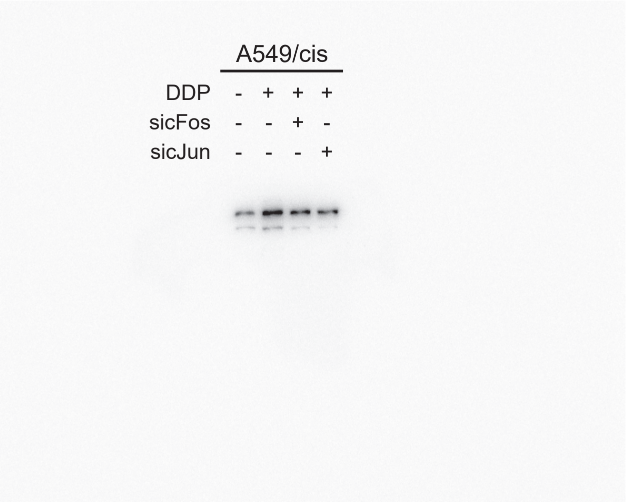

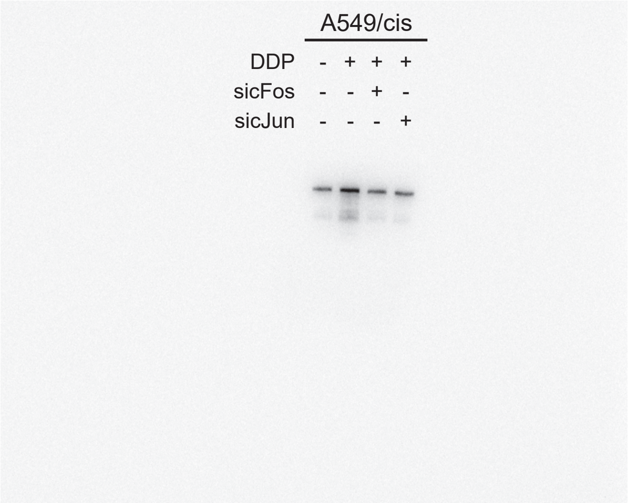

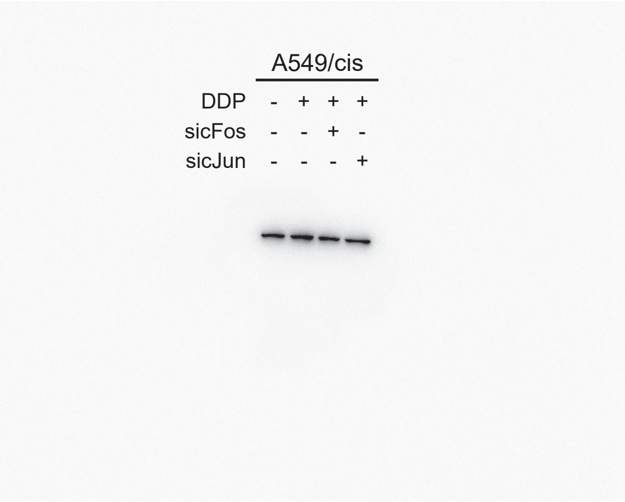

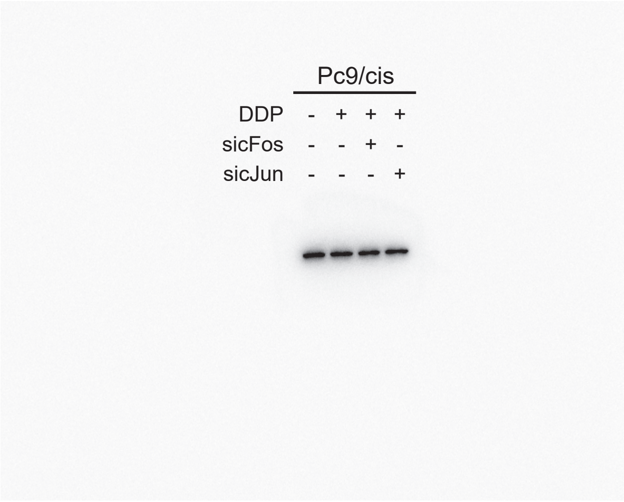

Uncropped Western blot images for Figure S4a

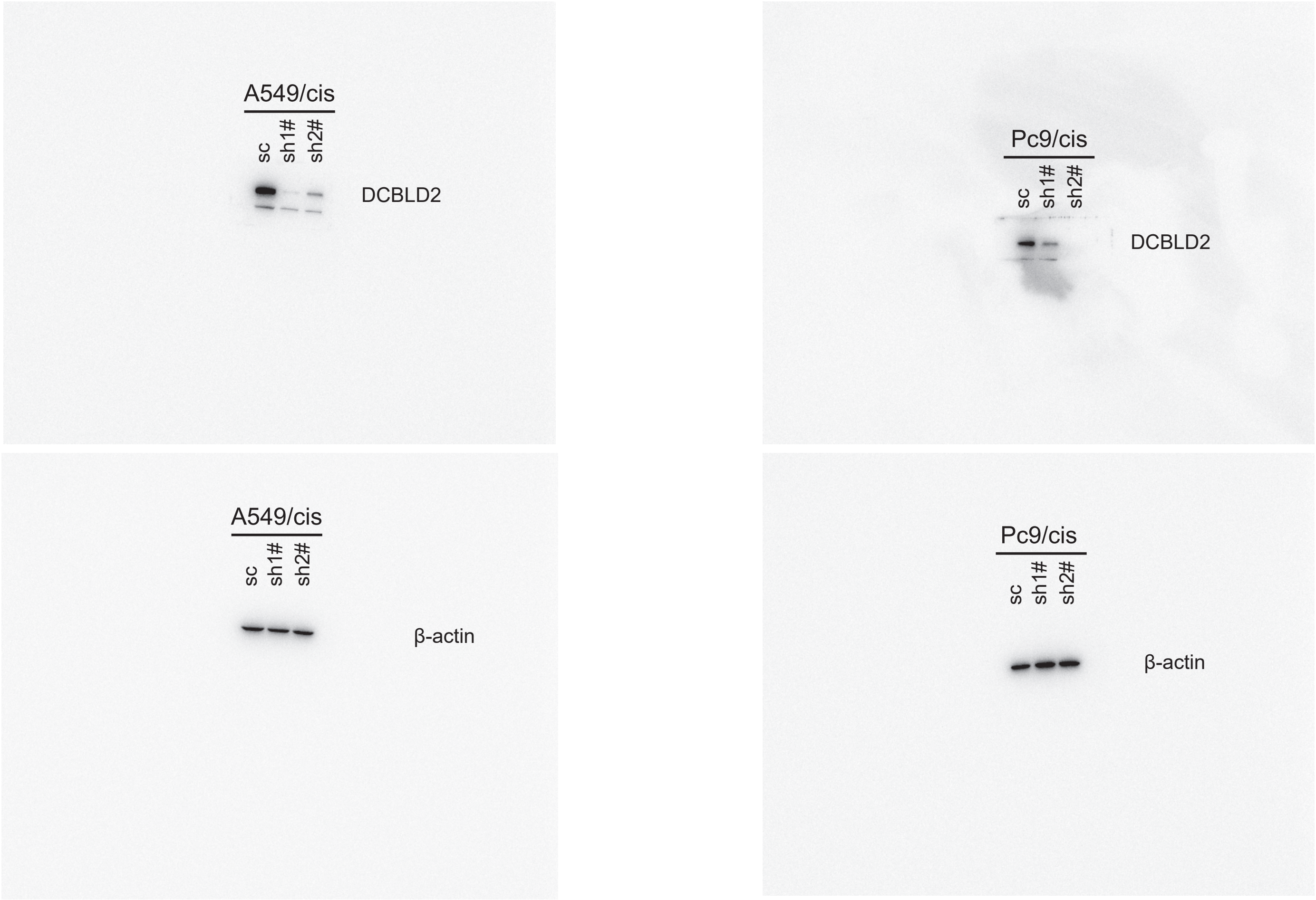

Uncropped Western blot images for Figure S5d

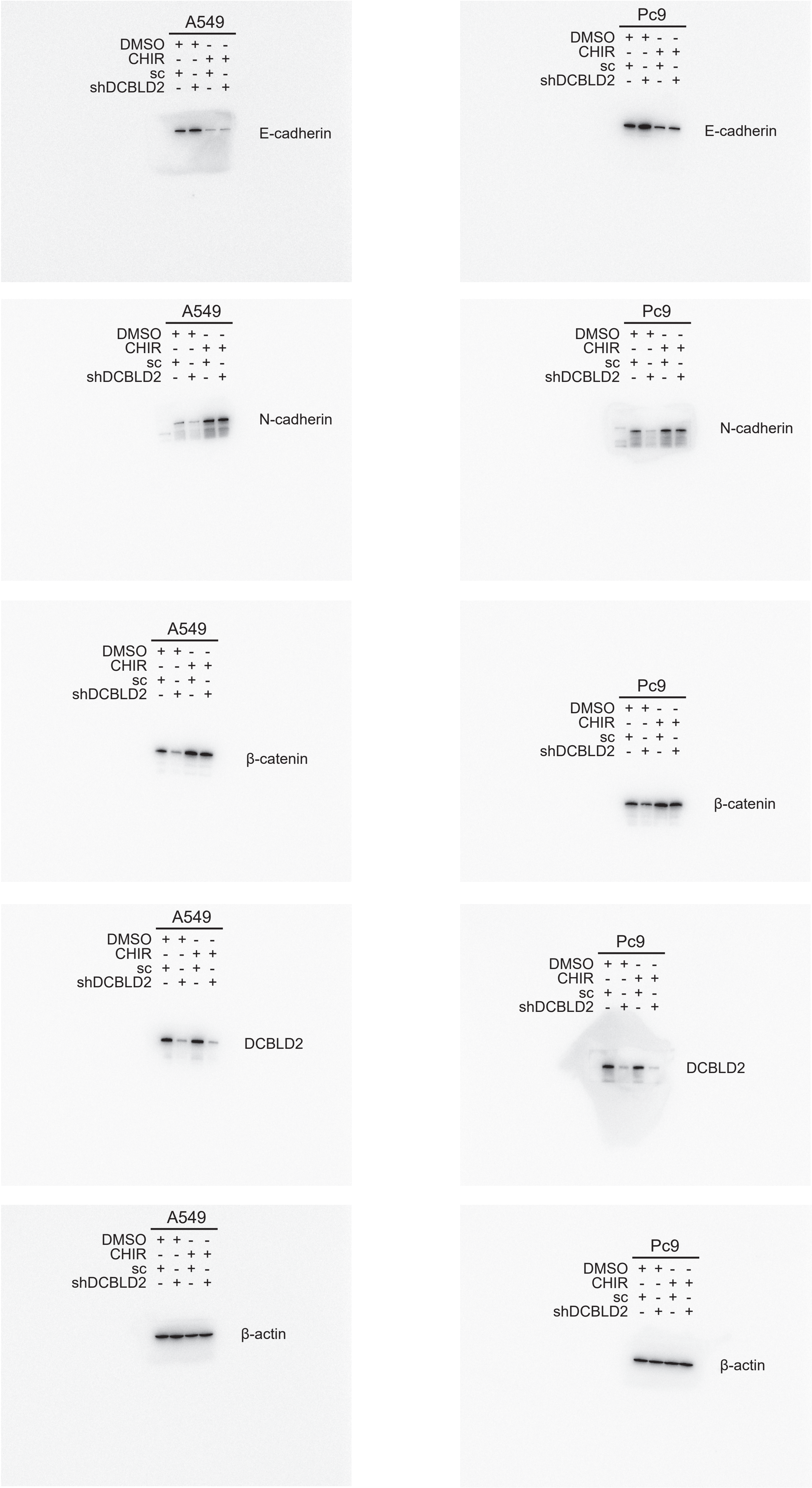

Uncropped Western blot images for Figure S6a

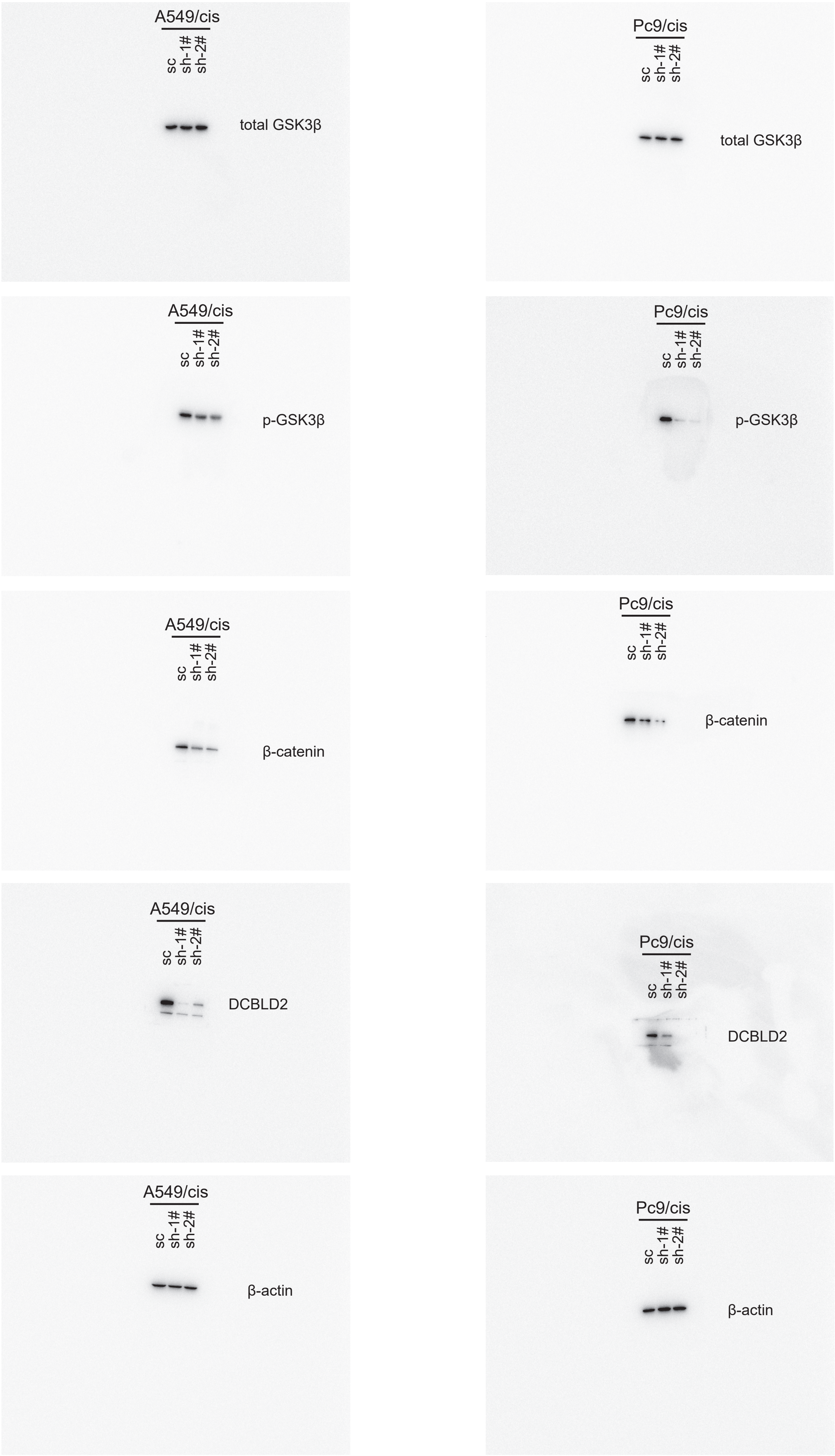

Uncropped Western blot images for Figure S6b

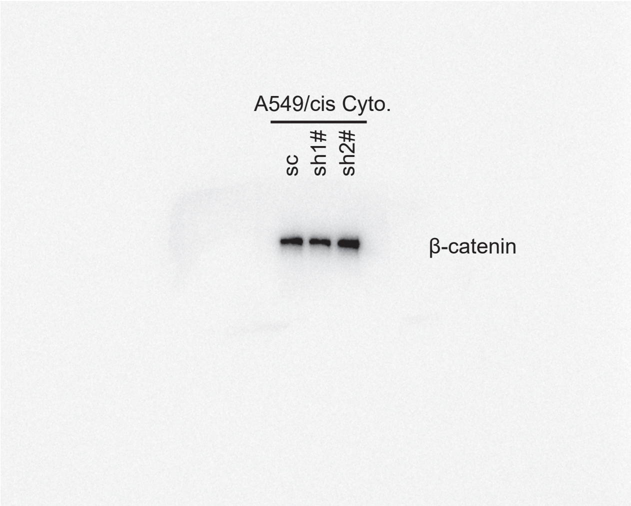

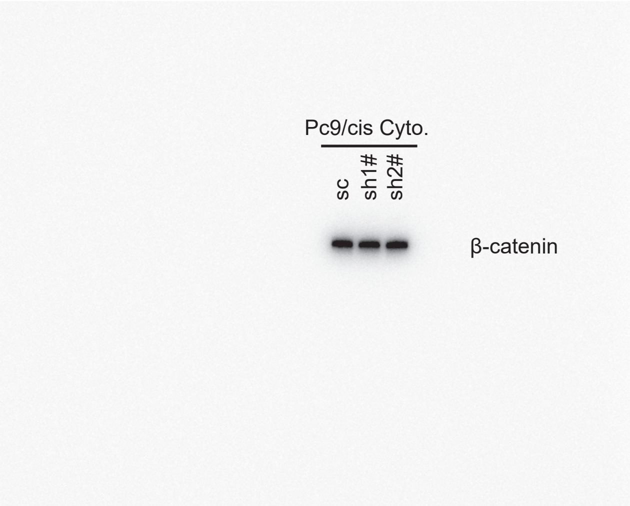

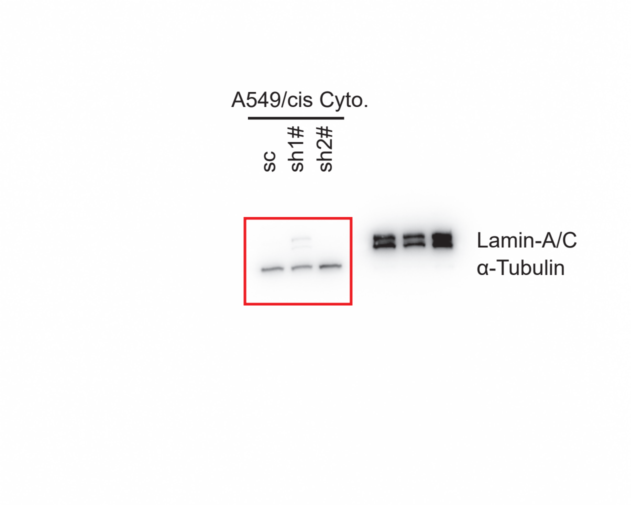

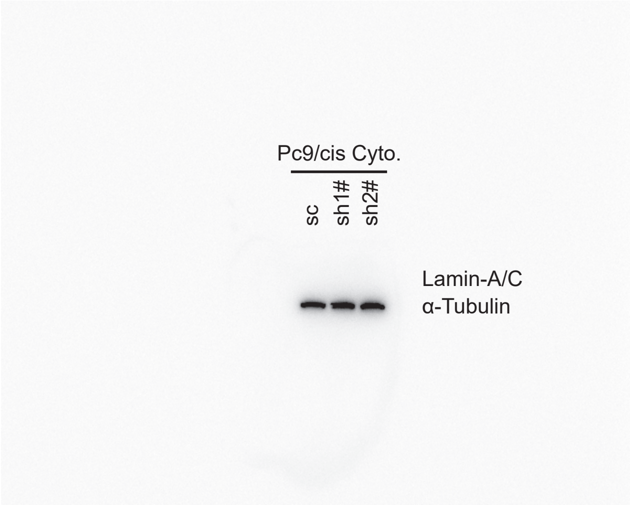

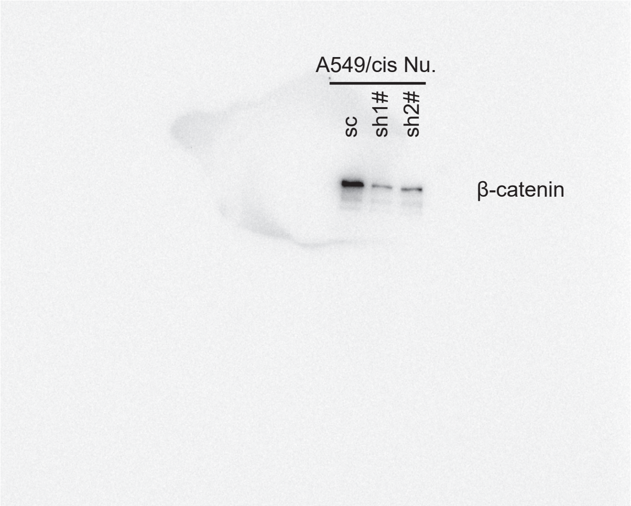

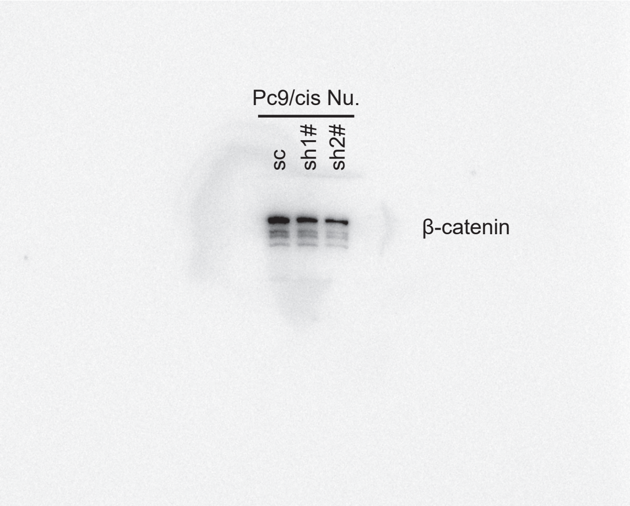

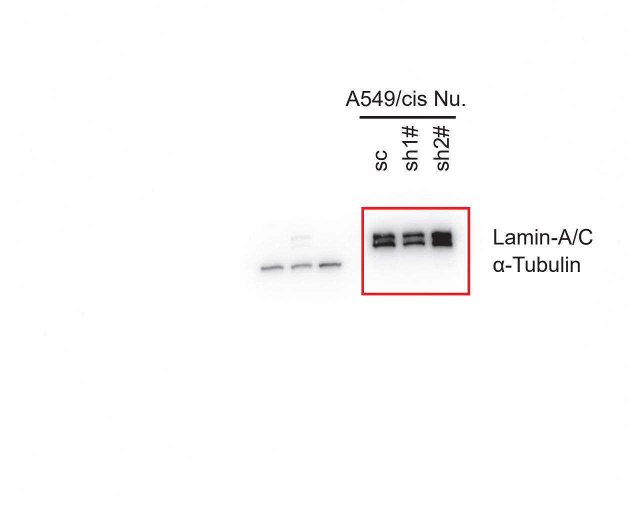

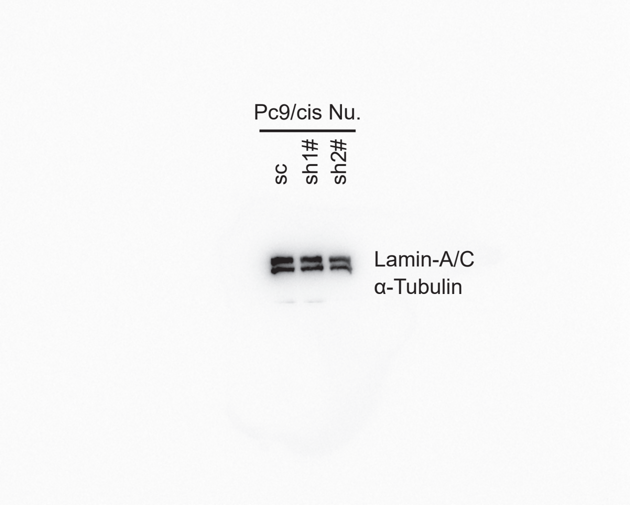

Uncropped Western blot images for Figure S6f

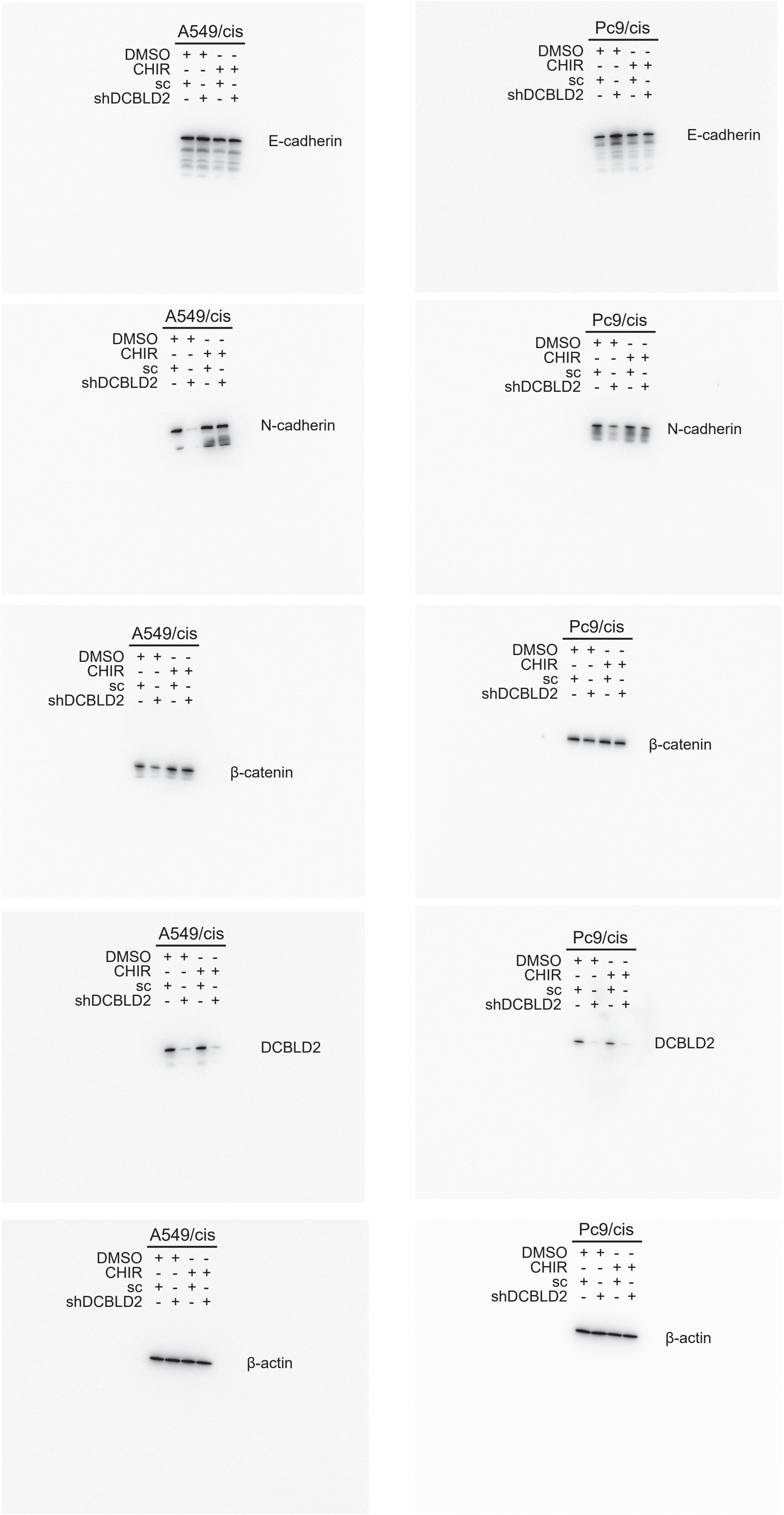

Supplement: Supplementary file 1 [file cancers-13-01403-s001.pdf]
